# Modified Guilu Erxian Glue restores immune tolerance in aplastic anemia by reprogramming T cell differentiation via the miR-146a/STAT1/SOCS1 axis

**DOI:** 10.3389/fimmu.2026.1826665

**Published:** 2026-05-29

**Authors:** Yingkai Zhang, Dongtian Bai, Song Sun, Wei Liu, Pingxin Zhang, Jingmin Niu, Jinghao Sang, Dongyang Li, Juan Liu, Limin Chai

**Affiliations:** 1 Key Laboratory of Chinese Internal Medicine of Ministry of Education and Beijing, Dongzhimen Hospital, Beijing University of Chinese Medicine, Beijing, China; 2 Beijing No.6 Hospital, Department of Gastroenterology, Beijing, China; 3 Beijing Hospital of Traditional Chinese Medicine, Capital Medical University, Beijing, China

**Keywords:** aplastic anemia, immune microenvironment homeostasis, miR-146a/STAT1/SOCS1 pathway, modified Guilu Erxian Glue, T cell proliferation and differentiation

## Abstract

**Objective:**

Two traditional Chinese medicine (TCM) formulas are Guilu Erxian Glue (GEG) and Danggui Buxue Tang (DBT). Their combination, Modified Guilu Erxian Glue (MGEG), is used to treat aplastic anemia (AA). This study aims to clarify how MGEG restores immune tolerance in AA mice. Specifically, we studied whether MGEG can regulate the differentiation and maturation of T cell lineage by regulating the transcription of miR-146a to rebuild the stable state of bone marrow immune microenvironment, inhibit the immune response mediated by T helper (Th)1 cells, and enhance the immunosuppressive function of regulatory T cells (Tregs).

**Materials and methods:**

MGEG’s active constituents and potential targets were identified using HPLC-ESI-MS and network pharmacology. Sublethal irradiation and immune cell infusion were used to create an AA mouse model. Bone marrow histopathology and peripheral blood parameters were used to assess therapeutic efficacy. T cell subsets and the maturation phenotypes of naïve/effector memory T cells were thoroughly examined using mass cytometry (CyTOF) and flow cytometry. ELISA was used to measure cytokine levels associated with T cell subsets and apoptosis. qPCR quantified miR-146a and its target gene, STAT1, expression levels. The expression levels of important proteins in the STAT1/SOCS1, Fas/FasL, and IL-2/STAT5 signaling pathways as well as lineage-defining transcription factors (T-bet, Foxp3, RORγ, GATA3) were assessed using Western blot.

**Results:**

Network pharmacology indicated MGEG primarily modulates JAK-STAT and apoptosis pathways. *In vivo*, MGEG elevated peripheral white blood cell and platelet counts, and alleviated hematopoietic failure. CyTOF and Western blot showed MGEG reprogrammed T cell differentiation: it suppressed Th1/Th17 cells (downregulating T-bet/RORγ) while restoring Foxp3 and promoting an upward trend in GATA3. MGEG boosted naïve T cells and diminished Tem accumulation, reactivating T cell immune reserve. Mechanistically, MGEG upregulated miR-146a, inhibiting the STAT1/SOCS1 pathway to block Th1-driven inflammation. Concurrently, it modulated Fas/FasL and IL-2/STAT5 pathways to inhibit Treg apoptosis and maintain functional stability.

**Conclusion:**

MGEG treats AA by constructing a multi-dimensional immunomodulatory network: it suppresses Th1-mediated inflammation via the miR-146a/STAT1/SOCS1 axis, preserves Treg function through the IL-2/STAT5 and Fas/FasL pathways, and corrects the imbalance between naïve and Tem cells, maintaining long-term immune reserve and homeostasis.

## 1 Introduction

Aplastic anemia (AA) constitutes a hematological condition featuring bone marrow (BM) deterioration and profound blood cell deficiency (1). Currently, immunosuppressive therapy (IST) and hematopoietic stem cell transplantation (HSCT) are the primary clinical treatment options (2, 3). Although the standard IST regimen, comprising anti-thymocyte globulin (ATG) combined with cyclosporine A (CsA), is widely used, it is associated with considerable rates of non-response, relapse, and adverse effects (4). While the incorporation of eltrombopag (EP), a small-molecule thrombopoietin receptor agonist, into first-line IST has significantly improved survival rates in AA patients, its application is still accompanied by certain adverse reactions (5). Therefore, the exploration of safer and more effective immunomodulatory strategies remains an unmet clinical need.

The imbalance between Th and Tregs plays a key role in the pathogenesis of AA, particularly the abnormal ratios of Th1/Th2 and Th17/Treg (6, 7). In individuals with AA, there is an overabundance of Th1 cells, which secrete high levels of inflammatory cytokines like interferon-γ. This triggers the death of hematopoietic stem cells by activating the JAK/STAT1 signaling pathway. Concurrently, Treg cells fail to exert normal immunosuppressive functions due to reduced expression of the key transcription factor Foxp3 and impaired IL-2/STAT5 signaling, leading to a breakdown of immune tolerance (8, 9). IL-2 and its downstream STAT5 signaling are decisive in regulating the maturation and proliferation of CD4^+^ T cell subsets (10), and defects in this pathway are a major cause of reduced Treg numbers in autoimmune diseases (11, 12). Furthermore, the pro-inflammatory microenvironment accelerates T cell overactivation and senescence, manifested as the exhaustion of naïve T cells and the abnormal accumulation of effector memory T cells (Tem), which further exacerbates immune-mediated bone marrow injury (13).

MicroRNAs (miRNAs) regulate T cell development and function and are critical epigenetic factors in maintaining immune tolerance (14). Among them, miR-146a is associated with the immunosuppressive capacity of Tregs (15). Studies have shown that miR-146a is like a “molecular brake”, which can target and suppress STAT1 and its downstream signals, thus preventing Th1 immune overreaction (16). If miR-146a is specifically removed from Tregs, the body’s tolerance to inflammatory damage caused by IFN-γ will be greatly reduced. Because miR-146a plays a key role in co-regulating Th1 response and Treg function, intervention against miR-146a may be a new method to help AA patients recover their immune tolerance.

According to the TCM theory, AA belongs to the categories of “Marrow Depletion”(Sui Lao) and” Consumptive Disease”(Xu Lao), which is considered to be caused by deficiency of kidney essence. Modified Guilu Erxian Glue (MGEG) is derived from the combination of Guilu Erxian Glue (GEG) from the Ming Dynasty text *Yi Fang Kao* and Danggui Buxue Tang (DBT) from the Jin-Yuan period text *Nei Wai Shang Bian Huo Lun*. MGEG possesses efficacy in tonifying the kidney to replenish essence and supplementing qi to nourish blood (17, 18). A clinical case study has reported that its combined application is highly effective in treating chronic AA (19). Modern pharmacological studies have confirmed that GEG can promote hematopoietic stem cell proliferation, improve the bone marrow microenvironment, delay senescence (20), and enhance immune defense (21). Meanwhile, DBT inhibits bone marrow cell apoptosis and improves hematopoietic function by regulating T lymphocyte differentiation and the JAK/STAT signaling pathway (22–24). Our previous animal experiments also showed that MGEG can increase the number of Treg cells in mice with AA, affect the Fas signaling pathway, and inhibits hematopoietic stem cell apoptosis (25, 26). Rather than being an experimental mixture conceived in a lab, MGEG possesses a long history of clinical optimization. Its practical value in safely normalizing blood parameters for chronic AA patients has been robustly validated by randomized controlled trials and comprehensive clinical reviews in medical settings (27, 28). Therefore, the mechanisms uncovered in our present work carry immediate translational weight, effectively linking these *in vivo* observations to established human treatments.

**Figure 1:**
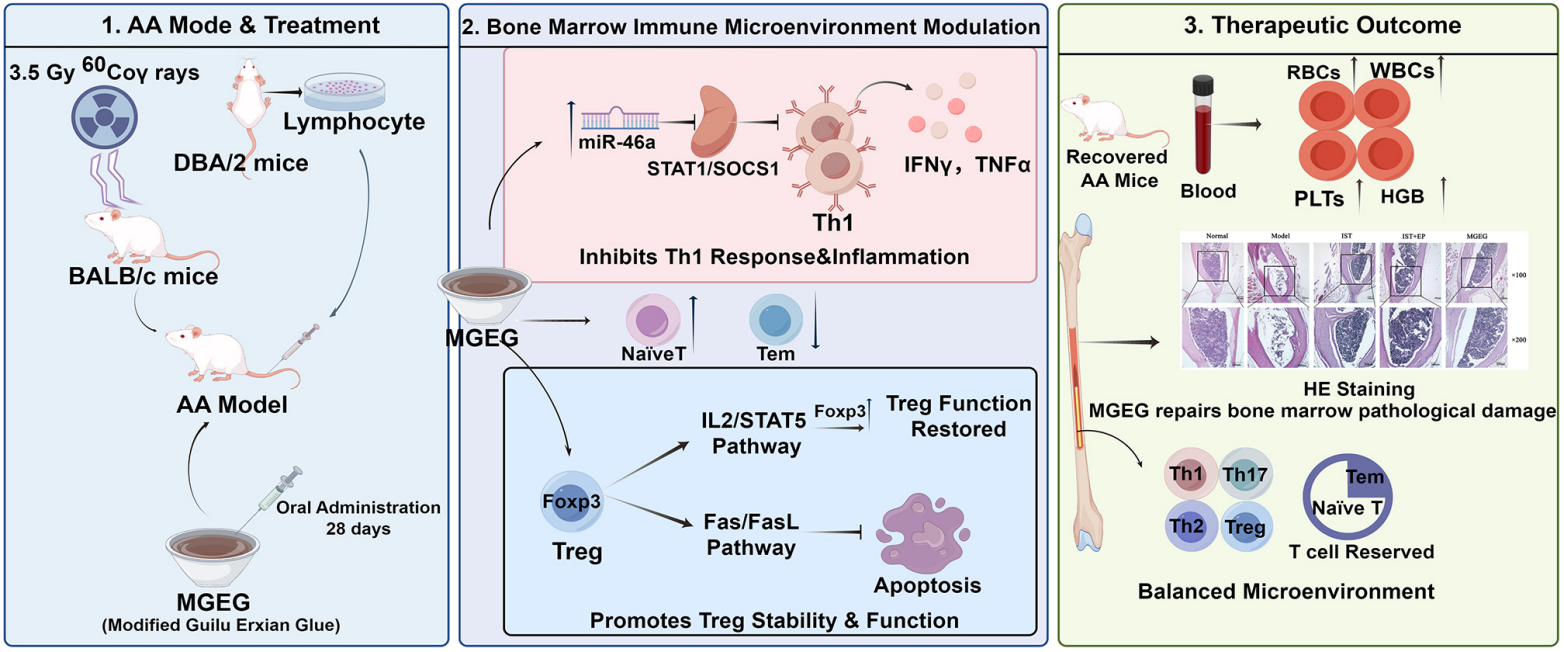
Schematic illustration of the multi-level mechanisms by which MGEG restores immune homeostasis in aplastic anemia. The image depicts: (1) AA Model and Treatment: Establishment of an immune-mediated AA model in BALB/c mice using sub-lethal irradiation and lymphocyte infusion, followed by 28 days of oral MGEG treatment. (2) Immune Modulation: MGEG suppresses Th1-driven inflammation via the miR-146a/STAT1/SOCS1 axis. It also stabilizes the Treg pool by activating IL-2/STAT5 survival signaling and inhibiting the Fas/FasL apoptotic cascade. (3) Therapeutic Outcomes: MGEG repairs bone marrow pathological damage, corrects the imbalance between naïve and effector memory T cells, and restores peripheral blood cell counts. AA, Aplastic anemia; MGEG, Modified Guilu Erxian Glue; Th1, T helper 1 cells; Treg, Regulatory T cells; Tem, Effector memory T cells; IFNγ, Interferon gamma; TNFα, Tumor necrosis factor alpha; TGF-β1 Transforming growth factor beta 1; IL-2, Interleukin 2; STAT1/5, Signal transducer and activator of transcription 1/5; SOCS1, Suppressor of cytokine signaling 1; FasL, Fas ligand; RBCs, Red blood cells; WBCs, White blood cells; PLTs, Platelets; HGB, Hemoglobin; HE, Hematoxylin-Eosin.

Traditional single-target research methods struggle to clarify the holistic mechanisms of TCM formulations due to their complicated composition and multi-target character. An effective method for understanding the “multi-component, multi-target, multi-pathway” processes of TCM formulae is network pharmacology (29, 30). The goal of this research is to use a method that combines “computational prediction and experimental validation.” After predicting the primary targets and pathways of MGEG using network pharmacology, we validated its mechanism of action in an AA mouse model. Specifically, we looked into whether MGEG interferes with miR-146a transcription to control the downstream STAT1/SOCS1, IL-2/STAT5, and Fas/FasL signaling axis. Consequently, we explored how this regulation rebalances the expression of key T cell transcription factors (T-bet, GATA3, RORγ, and Foxp3) and corrects the imbalance between naïve and memory T cells, thereby providing a deeper theoretical basis for MGEG in restoring immune homeostasis. The schematic diagram of the mechanism is shown in **Figure 1**.

## 2 Materials and methods

### 2.1 Preparation of MGEG herbal formula

MGEG is a compound formulation composed of seven traditional Chinese herbs: 10 g of Deer-Horn Glue (Lujiaojiao, 70117021601), 10 g of Tortoise-shell Glue (Guijiajiao, 70117041201), 20 g of *Lycium barbarum* L (717040701), 10 g of Ginseng Radix et Rhizoma (70117011001), 30 g of *Astragalus membranaceus* (7170104), 6 g of Coptidis Rhizoma (701020311), 6 g of Angelicae Sinensis Radix (717030101). Plant names have been verified at (www.worldfloraonline.org). All medicinal substances procured from Beijing University of Chinese Medicine’s Dongzhimen Hospital pharmacy. The preparation method was as follows: With the exception of Deer-Horn Glue and Tortoise-shell Glue, the other herbs were soaked in pure water for 30 minutes. After filtration, the herbs were boiled in 6 times the volume (w/v) of water for 30 minutes, and the extract was filtered. The residue was then boiled a second time in 8 times the volume of water for another 30 minutes. At this stage, Deer-Horn Glue and Tortoise-shell Glue were added to the second decoction and fully dissolved. The extracts from both boilings were combined and concentrated by heating to a final concentration of 1.18 g/mL (crude drug equivalent). Based on the dose-response preliminary experiments conducted by our group, the effects of different concentrations of MGEG on hematopoietic function and hematopoietic stem cells (HSCs) in AA mice were screened. The medium-dose group (equivalent to 11.96 g/kg of crude drug) was ultimately determined as the optimal dosage. This dose is effective in promoting hematopoietic recovery and has no obvious toxicity, so it was selected as the dose for the follow-up mechanism study (31–33).

### 2.2 Analysis of HPLC-ESI-MS

After the MGEG extract was filtered with a 150 μm sieve, we vacuum freeze-dried it at 4 °C. Then, we accurately weighed 20 mg of freeze-dried powder, added it to 5 mL of methanol-water solution (the ratio is 50:50 v/v), extracted it with ultrasonic wave for 20 minutes at room temperature, and then filtered it with 0.22 μm microporous membrane. Finally, we took 10 microliters of samples for analysis. In conducting the chromatographic separation, we utilized a Phenomenex Kinetex C18 column (100 mm × 2.1 mm, 2.6 μm) which was kept at a steady 35 °C throughout the analysis. The mobile phase was made up of a combination of 0.1% aqueous formic acid (Solution A) and acetonitrile (Solution B), with these components being introduced via gradient elution at a flow rate of 0.4 mL/min. While the UV detector was calibrated to 254 nm, full wavelength scanning was carried out across the 190–400 nm spectrum. Analysis via mass spectrometry, employing an electrospray ionizer, was conducted in both positive and negative ion operation modes. Instrumental parameters were optimized as follows: spray voltage set at ± 5500 V (adjusting for positive/negative modes), auxiliary gas temperature maintained at 600 °C, nebulizer gas (Gas1) calibrated to 50 psi, auxiliary gas (Gas2) at 60 psi, and curtain gas pressure set at 30 psi. Additionally, the declustering potential was established at ± 80 V, with collision energy fixed at ± 35 V. Data acquisition was managed via PeakView 2.2 software, employing an Information Dependent Acquisition (IDA) strategy throughout the analysis under nitrogen atmosphere.

### 2.3 Analysis of network pharmacology

This study used network pharmacology to anticipate MGEG’s possible processes based on the active components found by HPLC-MS. Initially, the SwissTargetPrediction (34) (filtered by probability > 0), HERB (35), and SEA databases were used to forecast component targets (36). Concurrently, disease-related targets were acquired from the Therapeutic Target Database (TTD) (37) and GeneCards (38), databases that use the keyword “Aplastic anemia”. To ensure the high relevance and reliability of the targets and to eliminate false positives, a strict filtering criterion was applied. Specifically, entries from the GeneCards database were filtered by setting a relevance score threshold of median value.

By connecting the component and disease targets, potential therapeutic targets were found. In order to search for core targets, a Protein-Protein Interaction (PPI) network was created using the STRING database (39), and a “Drug-Component-Target-Disease” network was created using Cytoscape 3.9.1 (40). Lastly, KEGG and GO enrichment analyses (*P* < 0.05) were performed via Metascape (41), and the Bioinformatics online platform to clarify the key signaling pathways by which MGEG regulates AA and to guide subsequent experimental validation (42).

### 2.4 Molecular docking simulation

The 3D crystal structures of the core target proteins were obtained from the RCSB Protein Data Bank (PDB, https://www.rcsb.org). The structural files (SDF format) of the key active components from MGEG were downloaded from the PubChem database (https://pubchem.ncbi.nlm.nih.gov). Protein preparation and molecular docking simulations were conducted using the CB-Dock2 web server (https://cadd.labshare.cn/cb-dock2). Briefly, the receptor proteins were preprocessed by removing water molecules, adding polar hydrogen atoms, and repairing missing residues. Subsequently, the binding affinities (kcal/mol) between the ligands and target proteins were calculated based on the AutoDock Vina algorithm. Finally, the optimal docking conformations with the lowest binding energies were selected and imported into BIOVIA Discovery Studio Visualizer 2025 to visualize and analyze the 2D and 3D intermolecular interactions.

### 2.4 Animals

This study utilized 75 female BALB/c mice (weighing 15–18 g) and 2 female DBA/2 mice (weighing 16–20 g), all purchased from Beijing Vital River Laboratory Animal Technology Co., Ltd. (License No.: SCXK [Jing] 2021-0006). The animals were kept at the Dongzhimen Hospital’s prestigious Barrier-Level Animal Research Facility, which is part of Beijing University of Chinese Medicine (license: SYXK [Jing] 2020-0013). The study was approved by the Animal Ethics Review Board of the hospital (approval code: 22-24).

### 2.5 Establishment of AA mouse model and grouping

Thymuses and lymph nodes were collected from 2 donor DBA/2 mice to prepare a single-cell suspension, adjusted to a density of 5×10^6^ cells/mL (Trypan blue exclusion rate > 95%). Fifteen BALB/c mice served as the normal group. The remaining mice received sub-lethal whole-body ^60^Co γ-ray irradiation (3.5 Gy) and were injected with 0.2 mL of the lymphocyte suspension via the tail vein within 4 hours post-irradiation to induce the AA model. Prior to irradiation and lymphocyte suspension transfer, baseline peripheral blood counts were evaluated (represented by the initial measurements of the normal group) to ensure the healthy and uniform physiological status of the mice (mean values: WBC 11.36 ± 2.03 × 10^9^/L, RBC 10.97 ± 0.81 × 10¹²/L, HGB 187.33 ± 21.13 g/L, and PLT 1222.50 ± 190.22 × 10^9^/L). To ensure baseline comparability prior to treatment, the modeled mice were stratified according to their body weights and subsequently divided into 4 groups using a computer-generated random number sequence: the model group (Model), the immunosuppressive Therapy group (IST), the IST combined with eltrombopag group (IST+EP), and the MGEG treatment group (MGEG). Administration began on the first day after modeling for all treatment groups. Because irradiation- and immune-mediated peripheral pancytopenia requires several days to fully manifest, successful modeling was strictly confirmed on day 7 post-modeling. The model was deemed successful based on a profound reduction in peripheral blood parameters in the model group compared to the baseline, reaching the following threshold levels: WBC < 1.5 × 10^9^/L, RBC < 9.0 × 10¹²/L, HGB < 155 g/L, and PLT < 250 × 10^9^/L.

### 2.6 Drug treatment

The drug dosages administered to the mice were scientifically calculated by translating the clinical doses used in adult AA patients using the body surface area (BSA) normalization method, applying a standard murine conversion factor of 9.1.

In the IST group, ATG (0.12 mg/d) was slowly injected via the tail vein at a dose of 6 mg/kg/day (equivalent to 0.12 mg/day for a standard 20 g mouse) for 5 consecutive days; CsA was administered via intragastric gavage at a dose of 1.5 mg/kg/day (0.03 mg/day) over 28 sequential days. In the IST+EP group, EP was administered via gavage at a dose of 22.5 mg/kg/day (0.45 mg/day) for 28 successive days, in conjunction with the routine IST therapy. In the MGEG group, the MGEG extract was administered by gavage once daily for 28 consecutive days at a dose of 11.96 g/kg/day (administration volume 10 mL/kg., 0.2 mL for a 20 g mouse). To rigorously control for administration-induced stress and ensure experimental comparability, the normal and model groups were subjected to identical handling procedures. Specifically, mice in these control groups received slow tail vein injections of equivalent volumes of physiological saline for the first 5 days, alongside daily oral gavage of identical volumes of saline for the entire 28-day period. Upon completion of the study, all animals were humanely put to sleep using anesthesia, achieved through an intraperitoneal shot of 0.3% sodium pentobarbital, with the dose calculated at 0.1 mL for every 10 grams of their body mass. This procedure ensured that the subsequent retrieval of tissues and samples could be conducted without complication.

### 2.7 Whole blood cell analysis

Blood samples measuring 10 μL were drawn from the tail veins of mice on days 1, 7, 14, and 28 of treatment, with each sample immediately mixed with 2 mL of PBS. Subsequently, we utilized an automated hematology analyzer (Nihon Kohden, Tokyo, Japan) to determine white blood cell (WBC), red blood cell (RBC), and platelet (PLT) counts, alongside hemoglobin (HGB) concentrations.

### 2.8 Bone marrow histopathology (HE staining)

At the end of the administration period, three mice were randomly selected from each group. One femur from each mouse was harvested and fixed in 4% paraformaldehyde for 48 hours. The samples were first rinsed with running water and then soaked in an EDTA solution for decalcification until the bones became soft. Subsequently, the samples were dehydrated through a graded alcohol series, cleared with xylene, embedded in paraffin, and sectioned. The sections were stained with HE and observed under optical microscope (DM RAS2, Leica, Germany) to see the degree of bone marrow hyperplasia and the changes of adipocytes.

### 2.9 Flow cytometry analysis

At the end of the administration period, we randomly selected six mice from each group. Following the procedure, we divided the spleen tissue of each mouse in half and created a single-cell suspension from it. We then treated these cells with 5 μL of FITC-linked anti-mouse CD4 antibody, 5 μL of APC-linked anti-mouse CD62L antibody, and 5 μL of PE-linked anti-human/mouse CD44 antibody. Subsequently, we incubated the mixture in the dark for a 15-minute period to observe the proportion of naïve T cells to Tem cells. We used FACS Calibur flow cytometer to analyze the samples, and the data were processed by Cell Quest software version 1.5.

### 2.10 Mass cytometry (CyTOF) analysis

Mice were first anesthetized, and blood was subsequently collected via retro-orbital sinus puncture and put it in a tube with 4% sodium citrate (1:9, v/v) to prevent coagulation. The PBS-diluted blood was gently applied above the lymphocyte separation medium. PBMCs were separated via density gradient centrifugation at 400 × g for 20 minutes. After these cells were washed and resuspended in PBS, we took 3× 10^6^ cells and stained them with 5 μM Cisplatin (37 °C, 5 min) to identify dead cells. After that, we added a cell stimulating cocktail (containing PMA, ionomycin, brefeldin A and monensin), and then cultured the cells in an incubator with 5% carbon dioxide at 37 °C for 5 hours. Next, we fixed the cells with 1.6% paraformaldehyde for 10 minutes, and then treated them with 0.1% saponin for 20 minutes to make the cell membrane permeable. Antibody labeling was done according to the instructions of MaxPar X8 Polymer Kits (Fluidigm): first, the surface markers were stained (placed at 4 °C for 30 minutes), then the cells were permeabilized, and then the markers in the cells were stained (placed at room temperature for 30 minutes). After all washing, we resuspended the cells in PBS solution containing EQ calibration beads, and finally detected them with Fluidigm Helios mass spectrometry flow system. We use X-shift clustering algorithm to analyze the data, and draw them with ViSNE diagram. All antibodies and their corresponding metal labels are listed in **Table 1**.

**Table 1:** Cell-labeled antibodies and their corresponding isotope-coupled metal labels.

Antibody	Metal label	Company
CD3 antibody	152Sm	Fluidigm
CD4 antibody	172Yb	Fluidigm
CD25 antibody	151Eu	Fluidigm
IFN-γ antibody	165Ho	Fluidigm
IL-4 antibody	166Er	Fluidigm
IL-17A antibody	174Yb	Fluidigm
T-bet antibody	163Dy	Fluidigm
GATA3 antibody	145Nd	Fluidigm
RORγt antibody	159Tb	Fluidigm
FOXP3 antibody	158Gd	Fluidigm

### 2.11 Enzyme-linked immunosorbent assay

We obtained blood samples by enucleation techniques, followed by centrifugation at 3000 rpm for a quarter-hour while maintaining a temperature of 4 °C to isolate the serum component. Using commercially available ELISA kits from Invitrogen (USA), we measured the serum levels of IFN-γ, TNF-α, IL-17A, IL-4, Fas, FasL, and TGF-β1. Throughout the entire process, we meticulously adhered to the protocols outlined by the manufacturer’s guidelines.

### 2.12 Real-time quantitative PCR

Magnetic bead sorting isolated CD4^+^ T cells from murine spleens, with subsequent RNA extraction via the HiPure RNA Mini Kit (Magen, China). RNA concentration and purity were measured using a spectrophotometer (A260/A280 > 1.8). 2 μg of total RNA was reverse-transcribed into cDNA using the All-in-One qRT SuperMix (Vazyme, China). The RT-qPCR reaction system was prepared according to the kit instructions. miR-146a-5p was detected using the TaqMan probe method with U6 snRNA as the internal control; STAT1 mRNA was detected using the SYBR Green dye method with GAPDH as the internal control. Expression values were determined by the 2^-ΔΔCt^ approach. Oligonucleotide primers appear in **Table 2**.

**Table 2:** Primer sequences.

Gene	Primer	Primer sequences
STAT1	STAT1-F	GGATCGCTTGCCCAACTCTT
	STAT1-R	TGGAGCAGAGCTGAAACGAC
GAPDH	GAPDH-F	AGGTCGGTGTGAACGGATTTG
	GAPDH-R	GGGGTCGTTGATGGCAACA
miR-146a	miR-146a-5pF	CGCCGGTGAGAACTGAATTCCATGGG
U6	U6-F	CTCGCTTCGGCAGCACA
	U6-R	AACGCTTCACGAATTTGCGT

### 2.13 Western blot analysis

CD4^+^CD25^+^Treg cell separation from splenic single-cell suspensions employed Magnetic Activated Cell Sorting (MACS). Briefly, cells were first incubated with Biotin-CD4/CD25 antibodies and anti-biotin microbeads for negative selection (removal of non-CD4^+^ cells). The flow-through was then incubated with anti-PE microbeads for positive selection and eluted through an MS column to obtain high-purity Treg cells. Protein Detection: We isolated total protein from Treg cells and spleen tissues, then measured protein concentrations employing the BCA assay technique. Following this, we subjected 20-30 μg of protein samples to separation via 10% SDS-PAGE before transferring them onto nitrocellulose membranes. To prevent non-specific binding, we blocked the membranes with 5% non-fat milk for one hour at room temperature, subsequently exposing them to primary antibodies overnight at 4 °C: rabbit anti-GAPDH, rabbit anti-STAT5, rabbit anti-p-STAT5 (Tyr694), rabbit anti-Caspase7, rabbit anti-cleaved Caspase7 (CST, USA, 1:1000); rabbit anti-p-Bcl2 (Ser70) (Affinity Bioscience, USA, 1:1000); mouse anti-Foxp3 (Boster, China, 1:2000); goat anti-Bcl2 (R&D Systems, USA, 1:500); human/mouse anti-Caspase8 (R&D Systems, USA, 1:2000); goat anti-Fas (R&D Systems, USA, 1:500); rabbit anti-STAT1 (p-S727), rabbit anti-SOCS1 (Abcam, USA, 1:1000); rabbit anti-STAT1 (CST, USA, 1:1000); mouse anti-T-bet (Santa Cruz, USA, 1:1000); mouse anti-GATA3 antibody (1:1000; Abcam, USA) and rabbit anti-ROR gamma antibody (1:5000; Abcam, USA). Following the washing step, the membranes underwent a one-hour incubation period with HRP-tagged secondary antibodies under ambient conditions. Subsequently, protein bands were made visible through the application of ECL chemiluminescent detection agents. Quantitative analysis of band intensity was performed utilizing ImageJ software, with normalization to GAPDH expression levels serving as the loading control for determining relative protein abundance.

### 2.14 Statistical analysis

The analysis was conducted using SPSS 20.0 for data processing and GraphPad Prism 10.1 for visualizations. All results are presented as the mean ± standard deviation (mean ± SD). We pre-checked for normal distribution prior to the analysis. For longitudinal data measured repeatedly over time (WBC, RBC, HGB, and PLT), a Two-way Repeated Measures ANOVA was employed to account for within-animal correlation, followed by Bonferroni’s *post-hoc* test. One-way ANOVA was reserved for single-time-point measurements (e.g., CyTOF, Western blot).

## 3 Results

### 3.1 Identification and analysis of chemical constituents of MGEG

**Figure 2:**
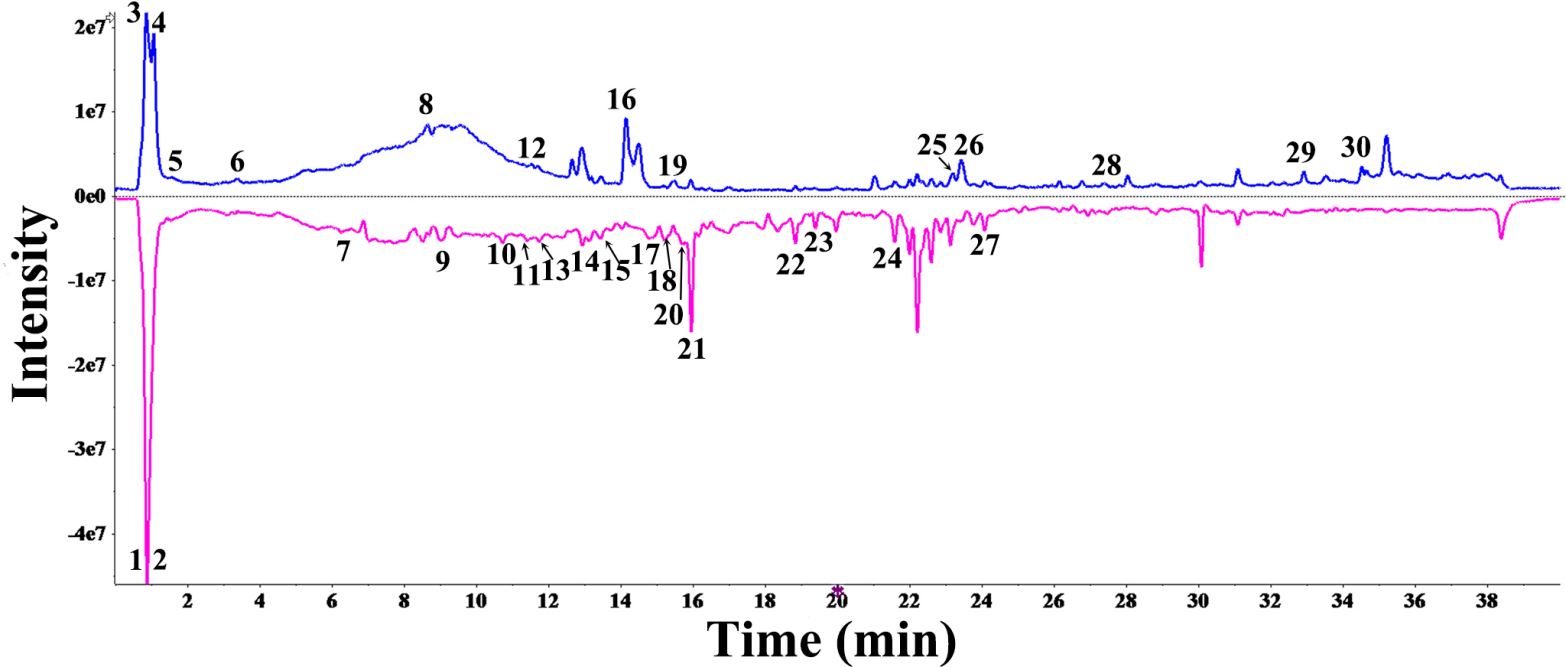
HPLC-ESI-MS/MS analysis of MGEG total ion current diagram (TIC). This picture shows the distribution of the main chemical components in the extract. HPLC-ESI-MS/MS, High-Performance Liquid Chromatography-Electrospray Ionization/Mass Spectrometry.

In order to fully understand which chemical components in MGEG are working, we systematically analyzed the samples by HPLC-ESI-MS/MS. The total ion current diagram (TIC, **Figure 2**) clearly shows the typical chromatographic characteristics of MGEG. By comparing with the standard sample and mass spectrometry database, we successfully determined the main chemical components (**Table 3**), which laid a solid foundation for studying its pharmacodynamic principle in the future.

**Table 3:** Identification of chemical components in MGEG by HPLC-ESI/MS.

Peak	tR, min	Formula	Identification
1	0.82	C_6_H_9_N_3_O_2_	Histidine
2	0.82	C_6_H_14_N_4_O_2_	Arginine
3	0.94	C_7_H_7_NO_2_	Trigonelline
4	0.97	C_30_H_18_O_10_	Amentoflavone
5	1.84	C_10_H_13_N_5_O_4_	Adenosine
6	1.98	C_10_H_13_N_5_O_5_	Guanosine
7	6.98	C_7_H_12_O_6_	Quinic acid
8	8.73	C_20_H_24_NO_4_+	Magnoflorine
9	9.49	C_11_H_12_N_2_O_2_	Tryptophan
10	10.72	C_22_H_22_O_10_	Calycosin-7-o-glucoside
11	11.41	C_27_H_30_O_16_	Rutin
12	11.70	C_21_H_22_NO_4_	Palmatine
13	11.75	C_20_H_24_O_9_	Nodakenin
14	12.93	C_16_H_12_O_5_	Calycosin
15	13.03	C_22_H_26_O_8_	(+)-Syringaresinol
16	13.19	C_20_H_20_NO_4_+	Jatrorrhizine
17	13.45	C_22_H_22_O_9_	Ononin
18	15.18	C_47_H_80_O_18_	Notoginsenoside R1
19	15.36	C_12_H_8_O_4_	Methoxsalen
20	15.43	C_16_H_12_O_4_	Formononetin
21	15.95	C_42_H_72_O_14_	Ginsenoside Rg1
22	18.98	C_41_H_68_O_14_	Astragaloside A
23	19.95	C_42_H_72_O_13_	Ginsenoside Rg2
24	21.77	C_26_H_43_NO_6_	Glycocholic acid
25	23.12	C_53_H_90_O_22_	Ginsenoside Rb2
26	23.35	C_19_H_20_O_5_	Decursin
27	24.14	C_41_H_66_O_12_	α-Hederin
28	27.48	C_33_H_58_O_14_	Gingerglycolipid B
29	33.41	C_20_H_18_NO_4_+	Berberine
30	33.74	C_32_H_50_O_13_	Rubusoside

### 3.2 Network pharmacology shows that MGEG regulates AA through JAK-STAT and apoptosis pathway

**Figure 3:**
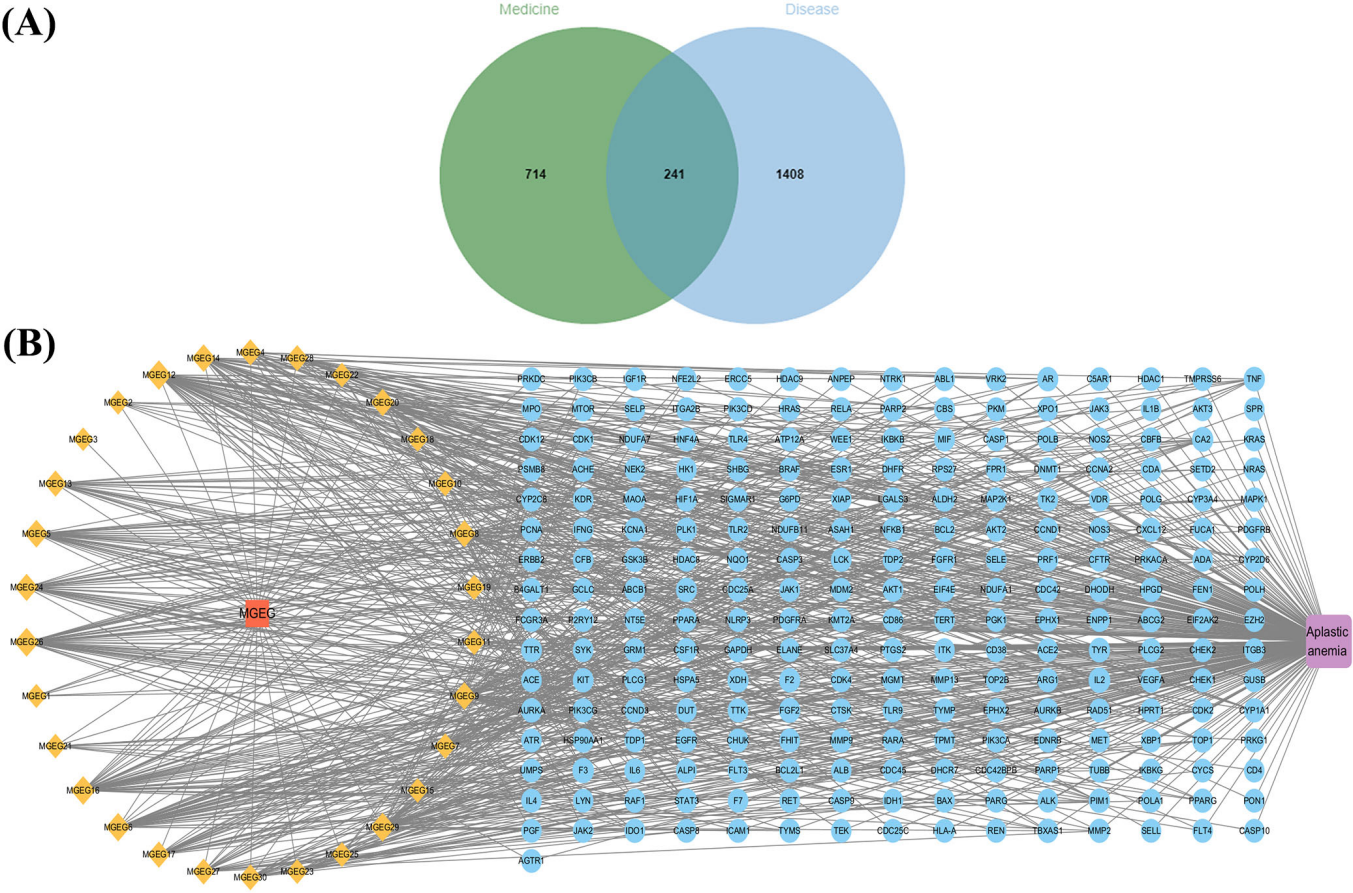
Network pharmacology analysis identifies core targets of MGEG in aplastic anemia. **(A)** Venn diagram showing the overlap between predicted targets of MGEG active components and aplastic anemia-related targets, yielding 241 common targets. **(B)** Drug–component–target–disease interaction network constructed using Cytoscape. Nodes represent MGEG, its active components, common targets, and AA; edges represent interactions. Node size corresponds to degree value, indicating connectivity within the network.

Through the active components detected by HPLC-MS, we found 241 common targets of MGEG and AA (**Figure 3A**), and drew a complete network diagram (**Figure 3B**) to show how these components work together.

**Figure 4:**
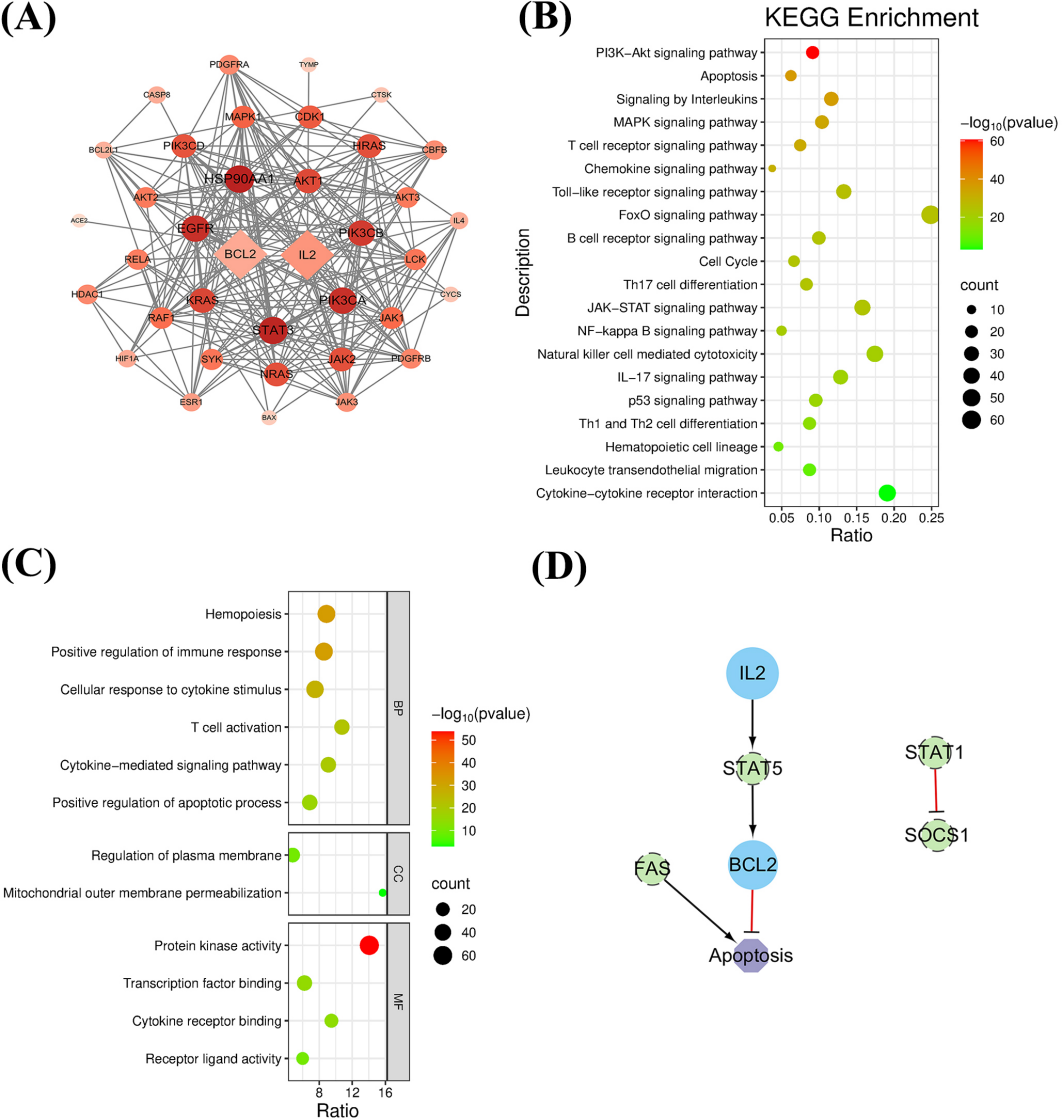
Core mechanistic subnetwork analysis reveals survival and inflammatory regulatory axes of MGEG in AA. **(A)** Protein–protein interaction (PPI) subnetwork highlighting IL-2 and BCL2 as central signaling hubs and their associated interacting proteins. Node size reflects degree value. **(B)** KEGG enrichment of common targets, indicating enrichment in immune-related signaling pathways. **(C)** Gene Ontology (GO) enrichment analysis showing the top biological processes (BP), cellular components (CC), and molecular functions (MF). **(D)** Core mechanistic subnetwork illustrating two principal regulatory axes: the IL-2/STAT5/BCL2 survival axis and the STAT1/SOCS1 inflammatory axis. In panel **(D)**, visual elements denote distinct functional categories rather than quantitative topological values: blue circular nodes represent core components of the cell survival axis; green circular nodes with hatching patterns designate regulators of inflammation and death receptor signaling; and the purple hexagonal node signifies the terminal biological process (Apoptosis). PPI, Protein-protein interaction; BCL2, B-cell lymphoma 2; KEGG, Kyoto Encyclopedia of Genes and Genomes; GO, Gene Ontology; BP, Biological processes; CC, Cellular components; MF, Molecular functions.

In order to study the molecular pathway behind it, we extracted a core subnetwork and focused on the functional regulatory axis linking the key targets IL-2 and BCL2 (**Figure 4A**). Topological analysis shows that although IL-2 and BCL2 are not the highest in the central ranking of the whole network, they are key signal hubs, and some important high connectivity proteins are connected around them, such as JAK1, STAT3 and PIK3CA (which are related to T cell proliferation), as well as HSP90AA1 and CASP8 (which are related to programmed cell death). Most importantly, IL-2 and BCL2 integrate a large number of signals through common intermediate nodes such as STAT3 and AKT1. This indicates that MGEG may activate the upstream JAK-STAT and PI3K-Akt signaling pathways by regulating these major effector molecules, thus restoring the immune balance.

KEGG enrichment analysis and GO classification (**Figures 4B, C**) confirmed that these targets were mainly concentrated in JAK-STAT signaling pathway, apoptosis, and T cell receptor signaling pathways. Therefore, we have constructed a core mechanism subnetwork (**Figure 4D**), which clearly depicts two key regulatory axes (1): IL-2/STAT5/BCL2 transcriptional survival axis, indicating that MGEG may inhibit the apoptosis of Treg cells through this pathway; (2) STAT1/SOCS1 inflammatory axis, suggesting that MGEG may inhibit excessive immunity by restoring this negative feedback cycle. These findings provide a clear theoretical basis for subsequent experimental verification.

### 3.3 Validation of molecular interactions through docking simulations

**Figure 5:**
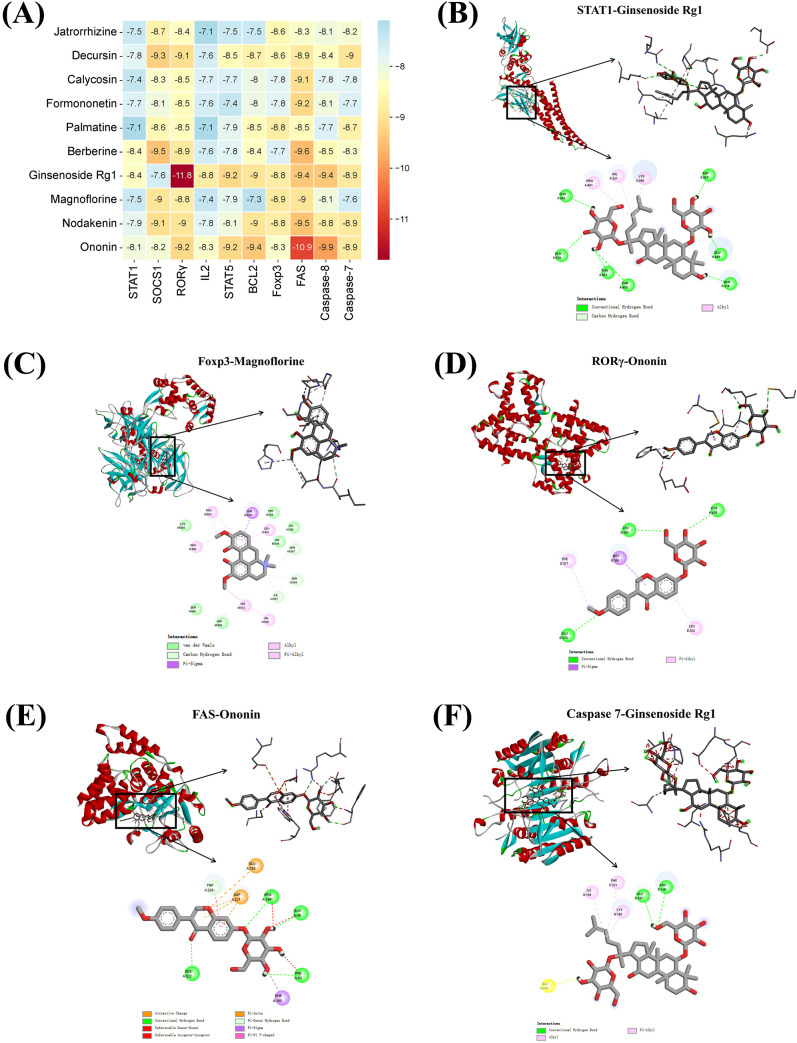
Molecular docking of MGEG components with core targets. **(A)** Heatmap of binding energies (kcal/mol) for 10 components and 10 targets. **(B–F)** 3D/2D interactions of key complexes: **(B)** STAT1-Ginsenoside Rg1 (-8.4); **(C)** Foxp3-Magnoflorine (-8.9); **(D)** RORγ-Ononin (-9.2); **(E)** FAS-Ononin (-10.9); **(F)** Caspase 7-Ginsenoside Rg1 (-8.9). Green dashed lines indicate conventional hydrogen bonds. Foxp3, Forkhead box P3; RORγ, RAR-related orphan receptor gamma; FAS, Fas cell surface death receptor.

To assess how MGEG constituents structurally engage with specific therapeutic targets, molecular docking simulations were carried out. The resulting interaction heatmap (**Figure 5A**) reveals that most evaluated active compounds possess strong affinities for the essential proteins. The majority of the binding energies dropped below -7.0 kcal/mol, reflecting the formation of stable molecular architectures.

Based on binding strength and biological importance, five critical receptor-ligand complexes were extracted for detailed interaction mapping. As the dominant chemical identified via mass spectrometry, Ginsenoside Rg1 effectively occupied the active pocket of STAT1 (-8.4 kcal/mol, **Figure 5B**) primarily through a network of conventional hydrogen bonds. Magnoflorine, a major alkaloid in the extract, formed a stable complex with the Treg-associated protein Foxp3 (-8.9 kcal/mol, **Figure 5C**). Regarding immune differentiation and death receptor signaling, Ononin displayed a broad targeting profile by deeply anchoring into both the Th17-regulating transcription factor RORγ (-9.2 kcal/mol, **Figure 5E**) and FAS (-10.9 kcal/mol, **Figure 5D**). Additionally, Ginsenoside Rg1 also showed a firm connection with the apoptotic executioner Caspase-7 (-8.9 kcal/mol, **Figure 5F**). Ultimately, these structural observations theoretically explain the regulatory actions of MGEG on the STAT1 cascade, T cell lineage commitment, and apoptosis.

### 3.3 MGEG effectively reverses bone marrow failure and improves hematopoietic function in AA mice

**Figure 6:**
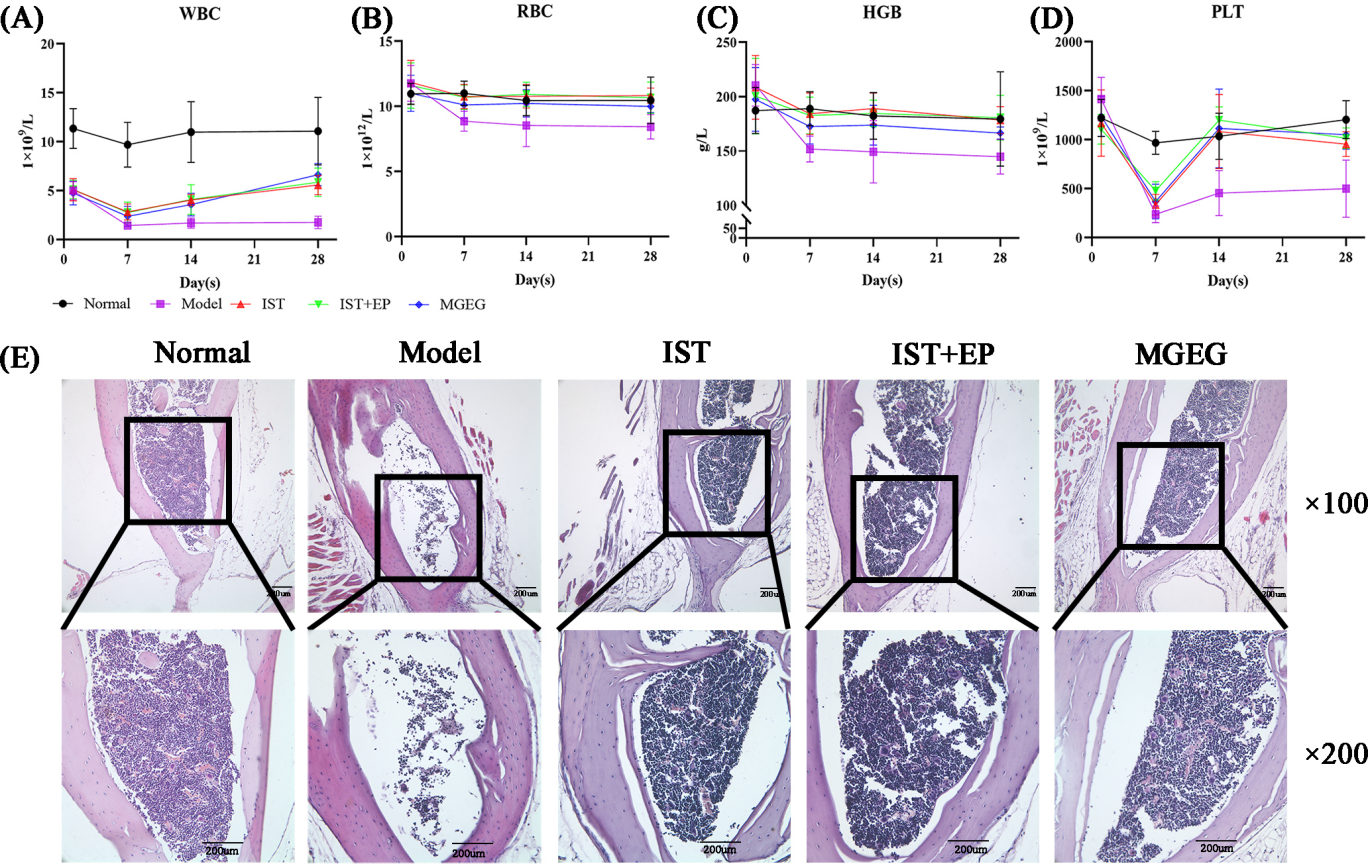
MGEG promotes hematopoietic recovery and ameliorates bone marrow failure in AA mice. **(A–D)** Dynamic changes in peripheral blood parameters including WBC, RBC, HGB, and PLT counts on days 1, 7, 14, and 28. The data is represented by mean ± SD, *n* = 6. Statistical significance for the overall recovery trajectories was determined using Two-way Repeated Measures ANOVA. MGEG treatment resulted in a significant overall improvement in WBC and PLT counts compared to the model group (*P* = 0.030 and *P* < 0.01, respectively). No significant overall main effects were observed for RBC (*P* = 0.133) and HGB (*P* = 0.499). **(E)** Representative HE staining images of femoral bone marrow pathology. The upper panels display low-magnification views (×100), and the lower panels show high-magnification views (×200). SD, Standard deviation; ANOVA, Analysis of variance.

MGEG treatment promoted hematopoietic recovery. To rigorously account for the within-subject correlation of peripheral blood counts measured repeatedly over time (Days 1, 7, 14, and 28), a Two-way Repeated Measures ANOVA was performed. The analysis validated successful model induction, showing a profound overall reduction in multilineage blood counts (WBC, RBC, HGB, and PLT) in the model group compared to the normal group (all *P* < 0.05). Importantly, MGEG administration effectively reversed the decline in specific lineages. The overall treatment trajectories for WBC and PLT were significantly elevated in the MGEG group compared to the model group across the 28-day period (*P* < 0.05 or *P* < 0.01). While the overall recovery trends for RBC and HGB in the MGEG group did not reach statistical significance against the model group (*P* > 0.05), the robust efficacy of MGEG in promoting WBC and PLT recovery was statistically comparable to the clinical first-line therapies (IST and IST+EP), with no significant differences observed among these treatment regimens (all *P* > 0.05) (**Figures 6A–D**). Bone marrow histopathology (HE staining) further confirmed that MGEG significantly repaired bone marrow pathological damage, increased the degree of hyperplasia, and promoted megakaryocyte regeneration (**Figure 6E**). To evaluate the *in vivo* safety of MGEG, we continuously tracked the general health of the animals over the four-week trial. Mice receiving the treatment maintained healthy phenotypes, characterized by steady weight gain, active movements, and well-groomed coats. We recorded zero instances of gastrointestinal distress, sluggishness, or premature death. Additionally, gross anatomical inspections at the endpoint revealed no visible lesions or structural abnormalities. Together, these findings confirm that the 11.96 g/kg dosing regimen is highly safe for sustained administration.

### 3.4 MGEG reshapes the differentiation balance and maturation status of T cell subsets in AA mice

Given that AA is a T cell-mediated autoimmune disease and network pharmacology analysis predicted the involvement of MGEG in immunomodulatory pathways, we combined mass cytometry (CyTOF) and Western blot to comprehensively evaluate the remodeling effect of MGEG on T cell immune homeostasis at both the cellular phenotype and key transcription factor levels.

**Figure 7:**
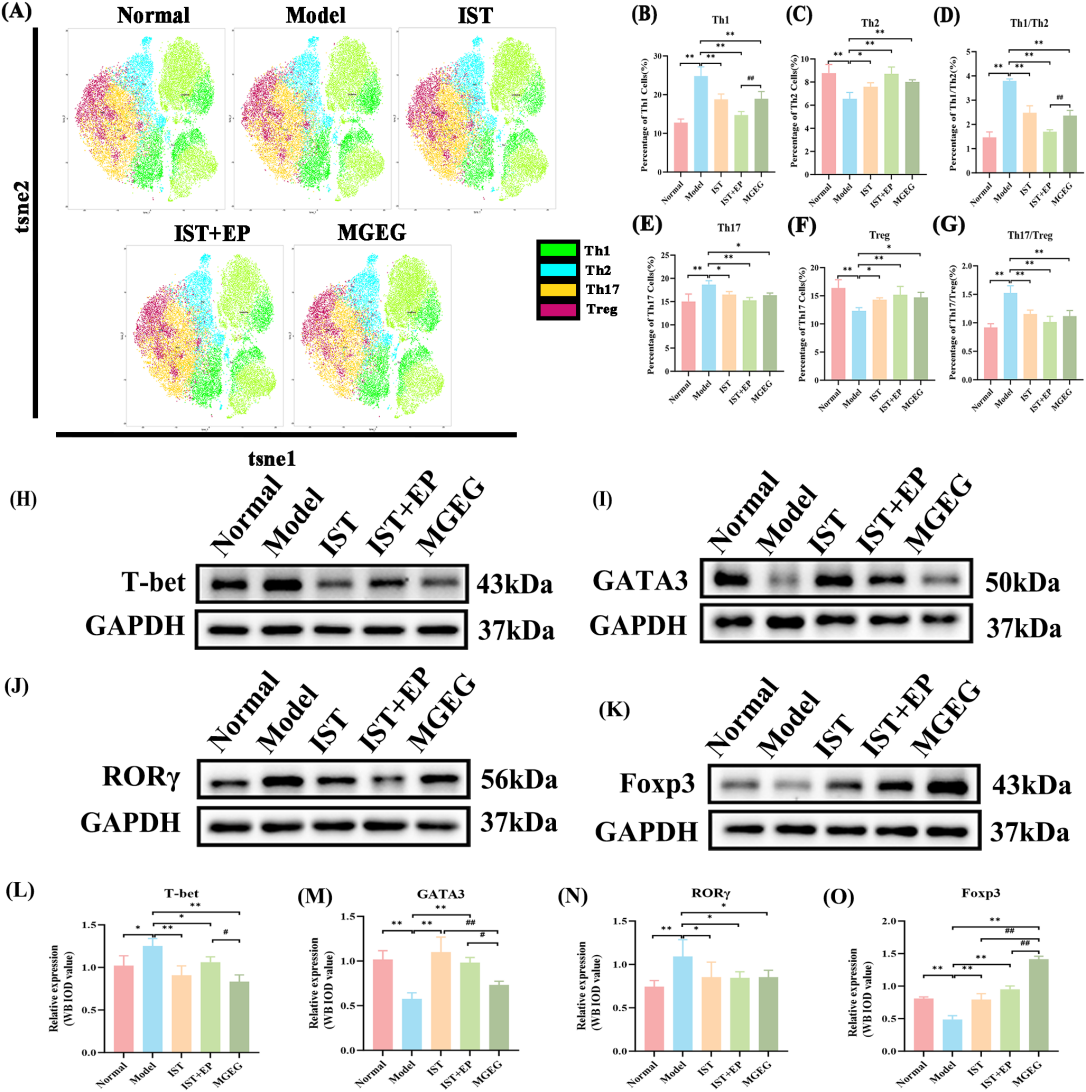
MGEG reshapes the differentiation balance of T cell subsets in AA mice. **(A)** t-SNE visualization of T cell subset distribution analyzed by CyTOF. **(B–G)** The ratio of Th1, Th2, Th17 and Treg cells in blood, and the ratio of Th1/Th2 and Th17/Treg. **(H–K)** Western blot bands of proteins (T-bet, GATA3, RORγ and Foxp3) which determine cell types in spleen tissue. **(L–O)** Measurement of the relative content of these proteins. The data is represented by mean ± SD, *n* = 3. The asterisk indicates that the difference is obvious, *P < 0.05, **P < 0.01 vs. model group; ^#^*P* < 0.05, ^##^*P* < 0.01 vs. MGEG group. Unless otherwise indicated, no statistically significant differences were observed between the MGEG group and the standard IST monotherapy group (*P* > 0.05). t-SNE, t-distributed stochastic neighbor embedding; CyTOF, Cytometry by time of flight;T-bet, T-box transcription factor TBX21; GATA3, GATA binding protein 3.

First, MGEG corrected the aberrant differentiation of CD4^+^ T cells that was skewed towards pro-inflammatory subsets. CyTOF results revealed a severe immune imbalance in the model group, characterized by aberrantly elevated proportions of pro-inflammatory Th1 and Th17 cells (*P* < 0.01) and significantly reduced proportions of anti-inflammatory Th2 and Treg cells (*P* < 0.01). To corroborate these phenotypic alterations at the molecular level, we examined the protein expression of lineage-defining transcription factors for each subset. In line with the flow cytometry findings, the Western blot revealed a notable boost in the levels of the Th1 master regulator T-bet and the Th17 cytokine driver RORγ in the spleens of the model group. Conversely, the Th2 controller GATA3 and the pivotal Treg mediator Foxp3 showed a decrease in expression. Post-treatment with MGEG, there was a considerable reduction in the percentage of Th1 and Th17 cells in the peripheral blood (*P* < 0.05 or *P* < 0.01), while the counts of Th2 and Treg cells were notably restored (*P* < 0.05 or *P* < 0.01) (**Figures 7A–G**). Correspondingly, the MGEG treatment group exhibited significantly downregulated protein expression of T-bet and RORγ in the spleen, alongside effectively restored protein levels of Foxp3 and an upward trend in GATA3 expression (**Figures 7H–O**). Notably, comparative analysis between the treatment groups revealed a nuanced immunomodulatory profile for MGEG. In suppressing pro-inflammatory drivers (such as T-bet and RORγ) and promoting a recovering trend in Th2-related GATA3, the efficacy of MGEG was statistically comparable or slightly inferior to the standard clinical regimens (IST and IST+EP). Most remarkably, however, MGEG demonstrated a significantly superior capacity to upregulate Foxp3 compared to both IST monotherapy and the IST+EP combination therapy. These findings confirm that while MGEG broadly matches the immunosuppressive effects of conventional drugs, it possesses a unique and potent advantage in specifically boosting the master transcriptional regulator of Treg cells, thereby fundamentally reshaping the equilibrium of T cell differentiation.

**Figure 8:**
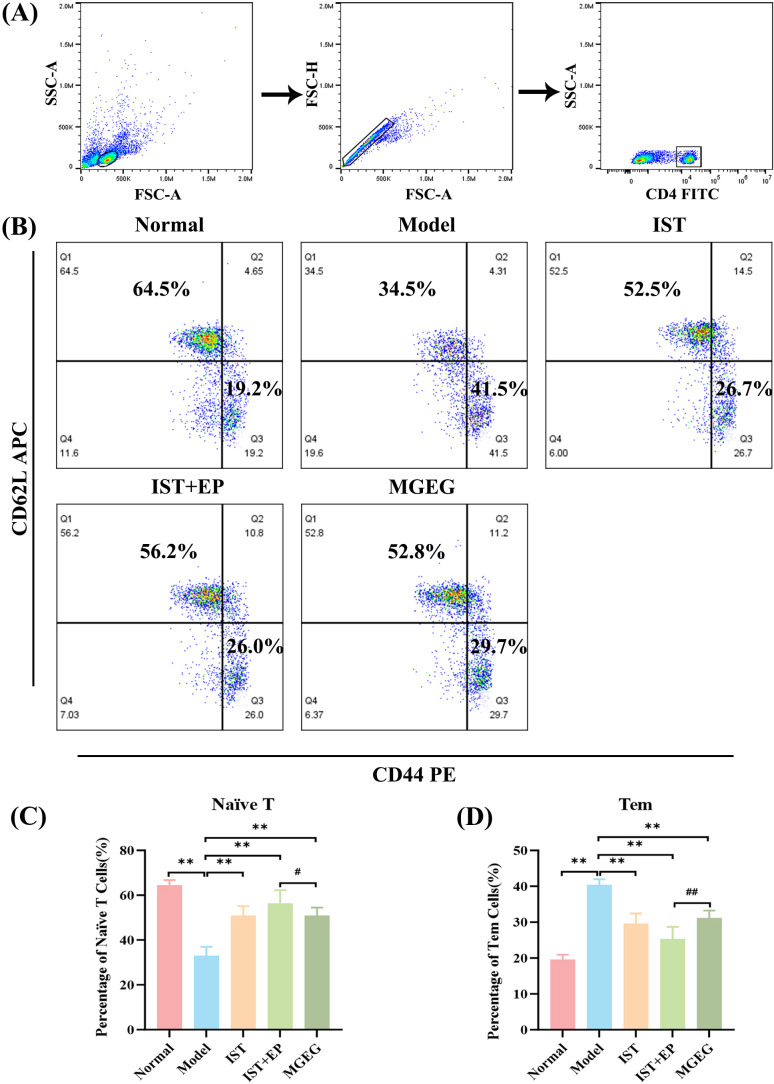
MGEG restores naïve and effector memory T cell balance. **(A, B)** The proliferation and differentiation of naïve T (CD4^+^ CD44^-^ CD62L^+^) and effector memory T (CD4^+^ CD44^+^ CD62L^-^), **(C, D)** Quantification analysis of naïve T, and Tem cells. Data are expressed as Mean ± SD, *n* = 6. Significance was indicated as *P < 0.05, **P < 0.01 vs. model group; ^#^*P* < 0.05, ^##^*P* < 0.01 vs. MGEG group. Unless otherwise indicated, no statistically significant differences were observed between the MGEG group and the standard IST monotherapy group (*P* > 0.05).

Moreover, MGEG can also help restore the reserve function of naïve T cell pool. We analyzed the mature state of T cells by flow cytometry, and found that T cells in the model group were obviously overactivated and depleted, which showed that the proportion of naïve T cells (CD62L^+^CD44^-^) decreased significantly (*P* < 0.01), while the proportion of (CD62L^-^CD44^+^) cells increased abnormally (*P* < 0.01). Importantly, after the intervention of MGEG, the proportion of naïve T cells increased significantly (*P* < 0.01), while the proportion of Tem cells decreased significantly (*P* < 0.01). Consistent with the subset analysis, MGEG effectively restored the naïve/memory T cell balance to a degree comparable to standard IST (*P* > 0.05). These results suggest that MGEG facilitates the maintenance of the reserve and renewal capacity of the naïve T cell pool, thereby preventing T cell overactivation and senescence (**Figures 8A–D**).

### 3.5 MGEG corrects serum cytokine disorder and rebuilds the immune microenvironment

**Figure 9:**
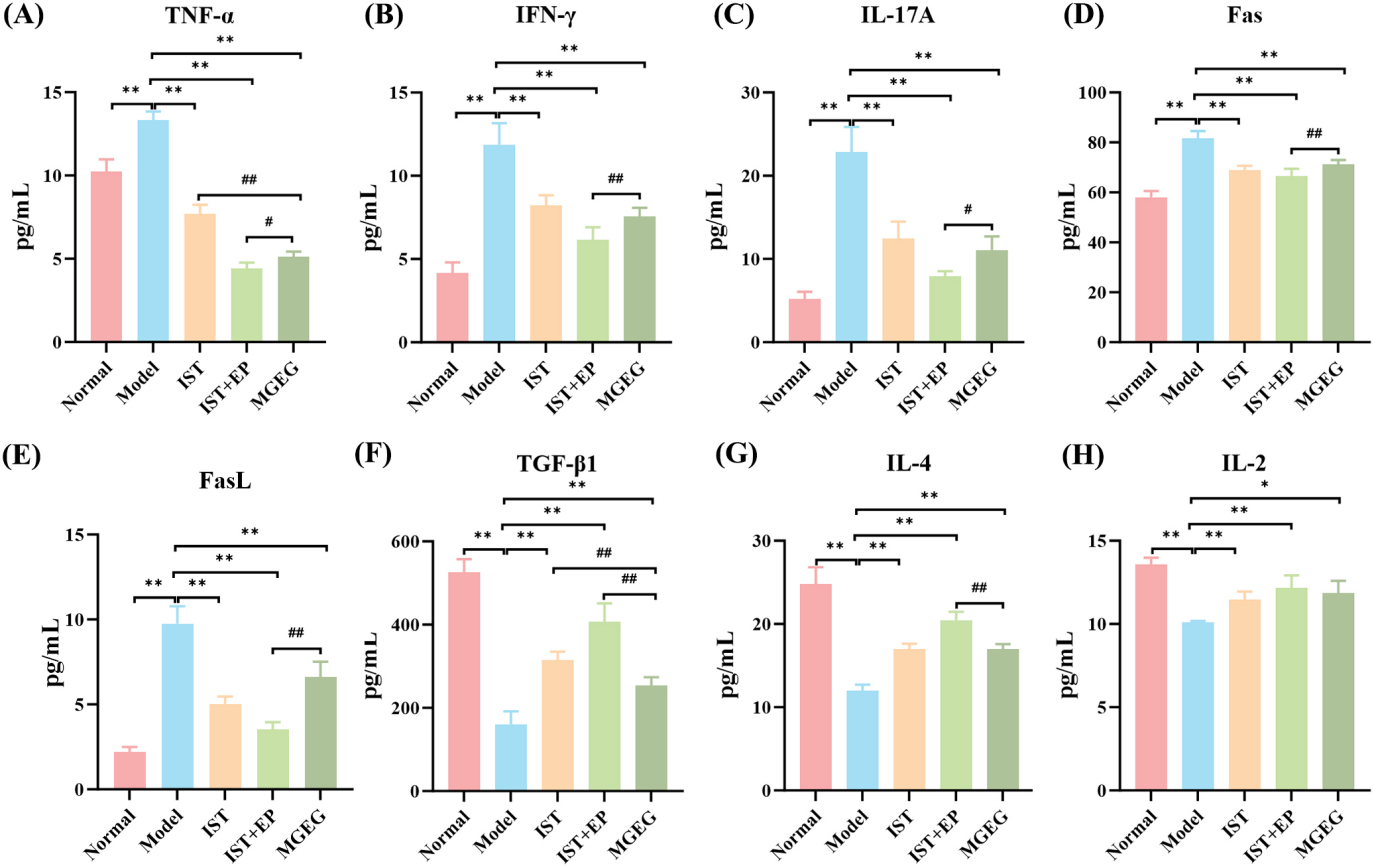
MGEG modulates serum cytokine profiles and apoptosis-related factors in AA mice. **(A-H)** ELISA analysis of serum concentrations of pro-inflammatory cytokines (TNF-α, IFN-γ, IL-17A), apoptosis-related factors (Fas, FasL), and anti-inflammatory cytokines (TGF-β1 IL-4, IL-2). Data are expressed as Mean ± SD, *n* = 6. Significance was indicated as *P < 0.05, **P < 0.01 vs. model group; ^#^*P* < 0.05, ^##^*P* < 0.01 vs. MGEG group. Unless otherwise indicated, no statistically significant differences were observed between the MGEG group and the standard IST monotherapy group (*P* > 0.05).

Compared to the model group, the MGEG intervention significantly attenuated the aberrantly elevated serum levels of inflammatory markers (TNF-α, IFN-γ, IL-17A) and apoptosis-associated factors (Fas, FasL), with results deemed statistically highly significant (*P* < 0.01). Simultaneously, MGEG significantly increased the concentrations of TGF-β1, IL-4, and IL-2 (*P* < 0.05 or *P* < 0.01), indicating that MGEG potently curtails overproduction of pro-inflammatory agents and concurrently stimulates the emission of anti-inflammatory factors. Notably, comparative analysis revealed that MGEG’s capacity to correct this cytokine disorder was statistically comparable to that of standard IST monotherapy, with no significant differences observed between the two groups (*P* > 0.05). While the IST+EP group exhibited a marginally stronger effect in regulating certain cytokines (such as TNF-α, IFN-γ, TGF-β1, and IL-4), MGEG independently demonstrated a robust and comprehensive ability to suppress pro-inflammatory cascades and promote an immune-tolerogenic microenvironment (**Figure 9**).

### 3.6 MGEG inhibits Th1 overactivation via the miR-146a/STAT1/SOCS1 axis

**Figure 10:**
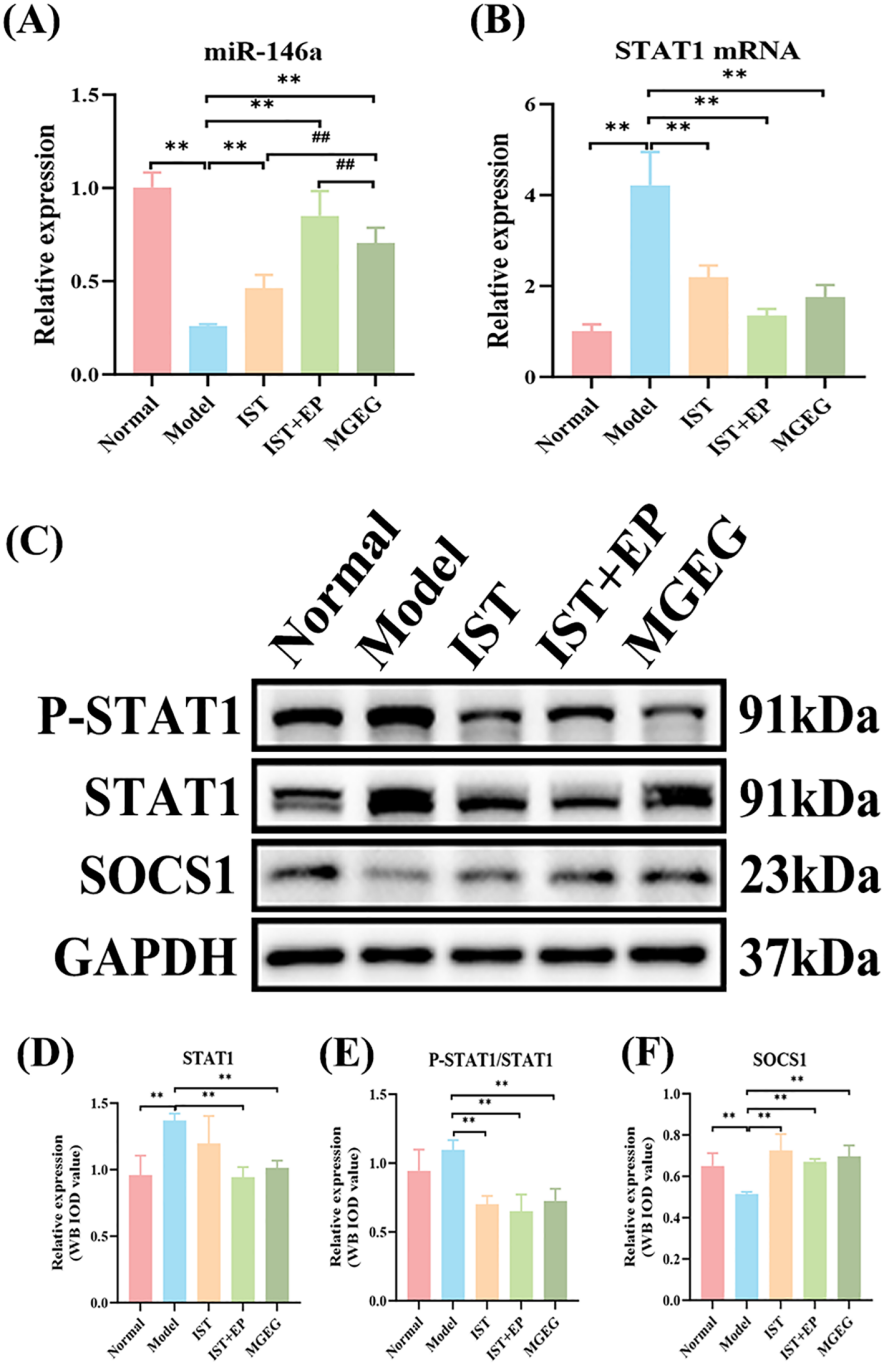
MGEG inhibits Th1 overactivation via the miR-146a/STAT1/SOCS1 axis. **(A, B)** Relative expression levels of miR-146a and STAT1 mRNA in CD4+ T cells determined by qPCR. **(C)** Western blot bands of p-STAT1, STAT1, and SOCS1 in spleen tissues. **(D–F)** Quantitative analysis of relative protein expression levels. For **(A, B)**, data are expressed as Mean ± SD, *n *= 6. For **(D–F)**, data are expressed as Mean ± SD, *n* = 3. Significance was indicated as **P < 0.01 vs. model group; ##*P* < 0.01 vs. MGEG group. Unless otherwise indicated, no statistically significant differences were observed between the MGEG group and the standard IST monotherapy group (*P* > 0.05). miR-146a, microRNA-146a; mRNA, Messenger RNA; qPCR, Quantitative polymerase chain reaction.

To investigate the molecular pathways by which MGEG suppresses Th1-driven inflammation, we focused on miR-146a and its target STAT1/SOCS1 signal axis. qPCR analysis revealed significantly reduced miR-146a expression in CD4^+^ T cells from the AA model group compared to the normal group (*P* < 0.01). Notably, MGEG treatment significantly upregulated the expression of miR-146a (*P* < 0.01). This restorative effect was statistically superior to that of the IST monotherapy group (*P* < 0.01), although it remained marginally lower than the highly immunosuppressive IST+EP combination therapy. Accordingly, the transcription of STAT1 mRNA was significantly down-regulated in the MGEG group (*P* < 0.01), achieving an inhibitory efficacy equivalent to both the IST and IST+EP groups (**Figures 10A, B**). Western blot analysis of spleen tissue further verified these results, showing that the levels of total STAT1 protein and its phosphorylated form p-STAT1 in the model group were abnormally increased, while the expression of negative regulatory factor SOCS1 was significantly decreased (*P* < 0.01), indicating that the inflammatory signaling pathway was over-activated. Following MGEG treatment, the protein expression and phosphorylation of STAT1 were significantly inhibited, while SOCS1 expression was effectively restored (*P* < 0.01). Consistent with the mRNA findings, the regulatory effects of MGEG on these key axis proteins (STAT1, p-STAT1, and SOCS1) were statistically comparable to the standard clinical regimens (IST and IST+EP), with no significant differences observed among the treatment groups (*P* > 0.05) (**Figures 10C–F**). Collectively, these results demonstrate that MGEG can upregulate miR-146a to target and abrogate STAT1 overactivation while restoring the SOCS1-mediated negative feedback loop, thereby disrupting the inflammatory signal transduction of Th1 cells.

### 3.7 MGEG reshapes Treg homeostasis via regulating Fas/FasL signaling pathway

**Figure 11:**
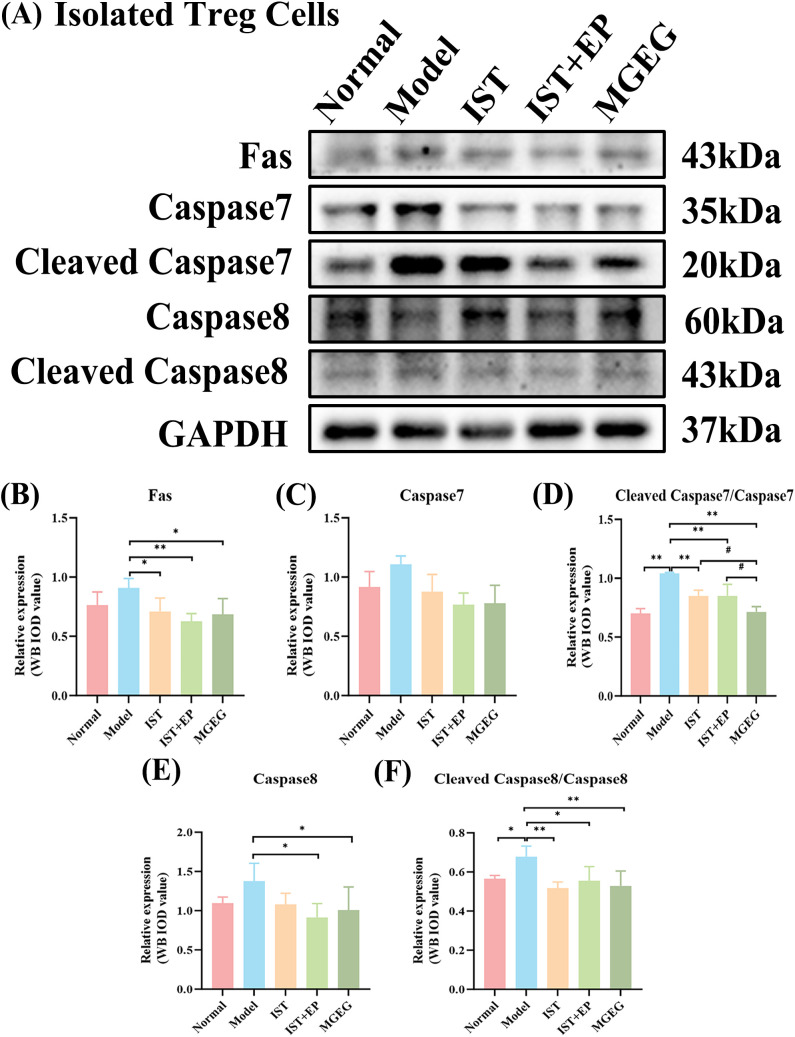
MGEG inhibits Fas/FasL-mediated Treg apoptosis. **(A)** Representative Western blot images of Fas, Caspase8, and Caspase7 in isolated Treg cells. **(B–F)** Densitometric quantification normalized to GAPDH. Data are expressed as Mean ± SD, *n* = 3. Significance was indicated as *P < 0.05, **P < 0.01 vs. model group; ^#^*P* < 0.05 vs. MGEG group. Unless otherwise indicated, no statistically significant differences were observed between the MGEG group and the standard IST monotherapy group (*P* > 0.05).

MGEG intervention effectively reversed the apoptotic trend of Tregs, significantly downregulating the expression of Fas, FasL, and key execution molecules Cleaved Caspase 7/8 (*P* < 0.05 or *P* < 0.01) (**Figures 11A–F**). Importantly, comparative analysis with the IST and IST+EP groups revealed that MGEG was exceptionally effective in halting this apoptotic cascade. For the upstream receptor Fas and the initiator Cleaved Caspase 8, the inhibitory efficacy of MGEG was statistically comparable to both the IST and IST+EP groups (*P* > 0.05). Most notably, in suppressing the terminal executioner Cleaved Caspase 7, MGEG demonstrated a significantly superior inhibitory effect compared to both the IST monotherapy and the IST+EP combination therapy (*P* < 0.05) (**Figures 11A–F**). This confirms that MGEG maintains the Treg pool by inhibiting Fas/FasL-mediated apoptotic signals.

### 3.8 MGEG reshapes Treg homeostasis via regulating IL-2/STAT5 signaling pathway

**Figure 12:**
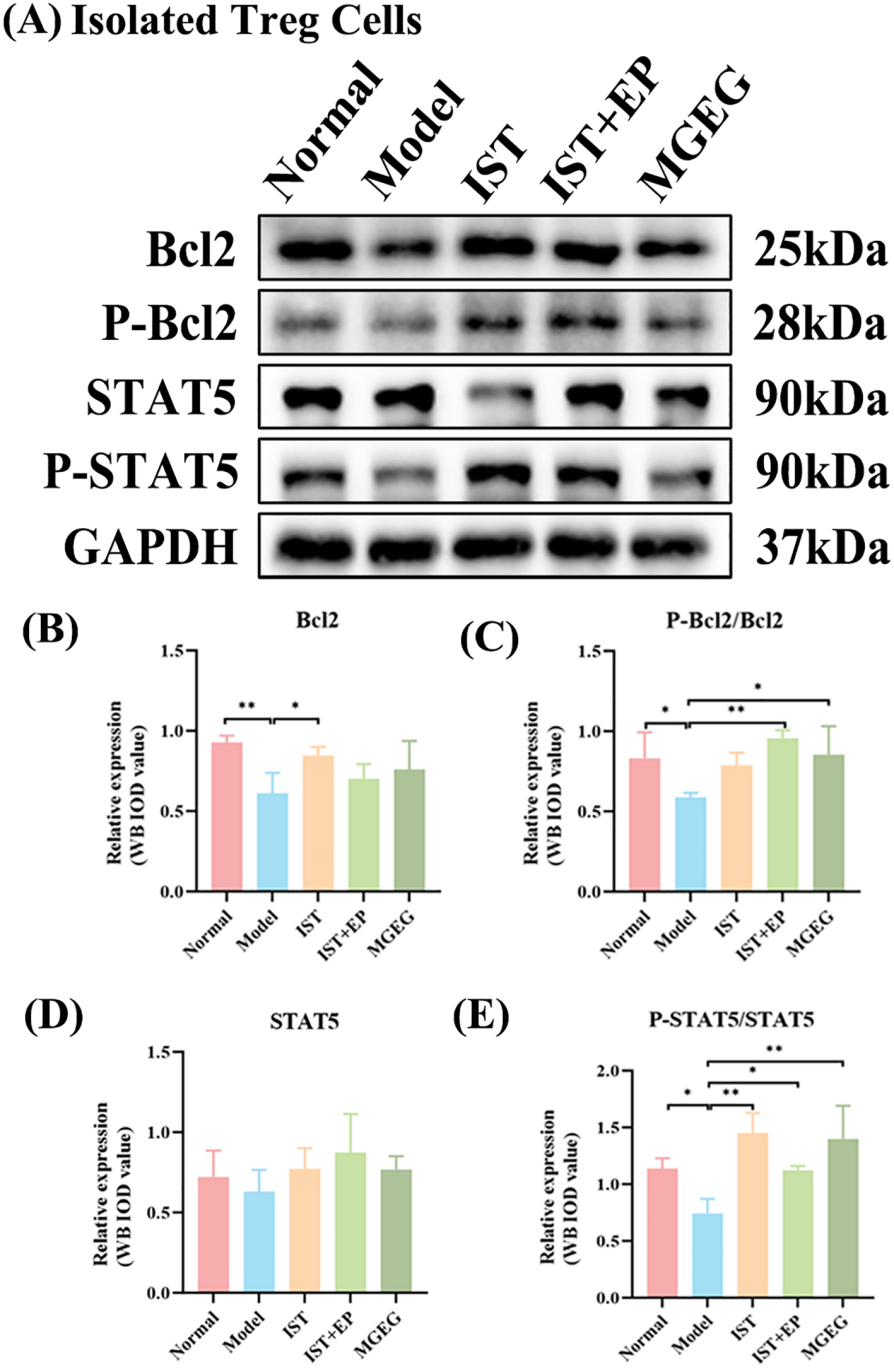
MGEG activates IL-2/STAT5 signaling to maintain Treg survival. **(A)** Representative Western blot images of STAT5, p-STAT5, Bcl2, and p-Bcl2 in Treg cells. **(B–E)** Quantitative densitometric analysis normalized to GAPDH. Data are expressed as Mean ± SD, *n* = 3. Significance was indicated as *P < 0.05, *P* <0.01 vs. model group. Unless otherwise indicated, no statistically significant differences were observed between the MGEG group and the standard IST monotherapy group (*P* > 0.05).

Our findings demonstrate that MGEG protects Treg cells from apoptosis primarily by triggering the IL-2/STAT5 signaling cascade. Interestingly, the baseline expressions of total STAT5 and Bcl2 proteins exhibited no statistical variation across the experimental groups (*P* > 0.05). Instead, the therapeutic impact manifested heavily at the post-translational level. Mice treated with MGEG showed a pronounced elevation in the phosphorylation ratios of P-STAT5 (Tyr694)/STAT5 and P-Bcl2 (Ser70)/Bcl2 relative to the untreated model (*P* < 0.05 or *P* < 0.01). Furthermore, this enhancement of pro-survival signaling fully matched the performance of standard clinical interventions, yielding outcomes statistically equivalent to both IST monotherapy and the combined IST+EP regimen (*P* > 0.05) (**Figures 12A–E**). Together, these data indicate that MGEG sustains the Treg reservoir by facilitating targeted phosphorylation events within the IL-2/STAT5 axis.

## 4 Discussion

AA is an autoimmune condition marked by bone marrow dysfunction affecting blood cell production and resulting in reduced blood counts. This study combined network pharmacological prediction and *in vivo* verification, and systematically revealed the immunomodulatory mechanism of MGEG in the treatment of AA. MGEG substantially enhanced blood cell counts and mitigated bone marrow pathology in AA mice. Notably, its efficacy in improving WBC and PLT was statistically comparable to standard IST. This is consistent with previous studies, indicating that Chinese herbal compound has a significant protective effect on bone marrow failure through the synergistic effect of multiple components (28, 43). Based on HPLC-MS analysis, we identified many active components in MGEG, such as ginsenoside Rg1, which can reverse hematopoietic dysfunction and inhibit HSC apoptosis (44); berberine, which can inhibit the activation of Th cells (45, 46); and astragaloside IV, which can enhance macrophage activity (47).

The core problem of AA is a fundamental breakdown in T cell immune tolerance, characterized by an overactive response from pro-inflammatory Th1 and Th17 cells, while regulatory T cells exhibit both quantitative and functional deficiencies (1, 48). Driven by transcription factor T-bet, Th1 cells will secrete a large amount of IFN-γ; this potent cytokine can not only amplify inflammatory signals, but also trigger systemic immune activation, thus disrupting the balance of bone marrow microenvironment (49, 50). At the same time, the expression of Foxp3 in AA patients decreased, which led to the decrease of the ability of Treg cells to produce anti-inflammatory cytokines, and the immunosuppression function was also impaired (6, 51). Our study confirmed that MGEG can regulate the balance of T cell subsets. Western blot analysis revealed that MGEG effectively suppressed the expression of pivotal transcription factors T-bet and RORγ while simultaneously reducing the concentrations of pro-inflammatory cytokines IFN-γ, TNF-α, and IL-17A. Conversely, the treatment revived Foxp3 expression and induced an upward trend in GATA3, elevating anti-inflammatory cytokine levels of IL-4 and TGF-β, which ultimately reestablished equilibrium between the Th1/Th2 and Th17/Treg immune pathways. Notably, this restorative capacity of MGEG was statistically comparable to that of standard clinical regimens (IST and IST+EP), highlighting its robust immunomodulatory potential. Our molecular docking analysis provides a robust structural basis for these observations: the high-abundance alkaloid Magnoflorine stably anchors to the Treg-specific transcription factor Foxp3, while the isoflavone Ononin tightly binds to the Th17 master regulator RORγ. Isoflavones like Ononin (and its aglycone formononetin) have been extensively reported to possess potent immune-skewing properties by suppressing Th17 lineage commitment (52). Simultaneously, emerging evidence highlights that Magnoflorine exhibits robust capacities to modulate systemic autoimmune inflammation and T cell dynamics (53). This direct molecular intervention likely contributes to the restorative capacity of MGEG, which reestablished immune equilibrium comparable to standard clinical regimens.

Moreover, the differentiation and maturity of T cells is an important index to evaluate the organism’s immune reserve ability. AA patients often show “senescent” immune characteristics, specifically the depletion of naïve T cells and the aberrant accumulation of Tem cells induced by chronic antigen stimulation, which is closely associated with disease progression and relapse (13). Our study found that MGEG therapy can significantly increase the proportion of naïve T cells, while reducing the proportion of Tem cells, achieving a restorative balance comparable to standard IST. This shows that MGEG can not only inhibit the active inflammation, but also prevent the premature exhaustion of T cells, which can protect the organism’s ability of long-term immune surveillance and self-renewal.

To further elucidate the molecular mechanisms by which MGEG inhibits Th1 overactivation, we studied an important protein STAT1 in JAK-STAT pathway and its “braking” protein SOCS1. MiRNAs play a critical role in regulating the immune system (14); Among them, miR-146a is especially related to the immunosuppressive ability of Tregs cells (15). MiR-146a maintains immune balance by specifically targeting and downregulating the expression of STAT1. In miR-146a-deficient models, the STAT1 protein will accumulate abnormally, which will interfere with the normal work of SOCS1 (16). STAT/SOCS signaling pathway plays a key role in maintaining systemic immune balance (54, 55). Our study found that MGEG treatment significantly increased the transcription level of miR-146a in CD4^+^ T cells of AA mice, exerting a restorative effect that was statistically superior to IST group. Accordingly, MGEG significantly inhibited the expression of STAT1 protein and restored the level of SOCS1, successfully achieving a downstream inhibitory efficacy equivalent to the IST+EP group. Crucially, molecular docking revealed a strong binding affinity between Ginsenoside Rg1 (the most abundant component in MGEG) and STAT1. By directly occupying the STAT1 pocket, Ginsenoside Rg1 may sterically hinder its activation. This structurally supports recent pharmacological findings that Ginsenoside Rg1 acts as a potent anti-inflammatory agent by directly interfering with STAT1-mediated signaling cascades (56). This evidence indicates that MGEG may exert its effects via the miR-146a/STAT1/SOCS1 epigenetic axis, blocking the IFN-γ/STAT1-mediated inflammatory response and thereby promoting the restoration of immune balance (57).

Although our findings establish miR-146a as a central regulatory node in suppressing Th1 overactivation, the precise upstream mechanisms driving its transcriptional restoration by MGEG require elucidation. We propose a compound-driven hypothesis based on the bioactive constituents identified in our HPLC-MS analysis. Extensive pharmacological evidence indicates that the principal components of MGEG, such as Berberine and Astragaloside IV, actively modulate immune-related gene transcription and suppress Th cell activation (45, 47). Simultaneously, Ginsenoside Rg1—which demonstrated strong binding affinity to STAT1 in our molecular docking analysis (−8.4 kcal/mol)—possesses well-documented capacities to regulate intracellular signaling cascades and hematopoietic function (44, 56). Given that miR-146a expression is highly dependent on specific promoter-binding transcription factors, we hypothesize that MGEG actively upregulates miR-146a through the combined transcriptional modulation exerted by these core constituents. In contrast, the partial restoration of miR-146a observed in the IST group likely reflects an indirect consequence of reduced systemic inflammation following T cell depletion, rather than active promoter engagement. This mechanistic distinction is consistent with our observation that IST monotherapy achieved significantly inferior miR-146a upregulation compared to MGEG (*P* < 0.01).

In addition to inhibiting Th1 inflammation, it is another key point to treat AA to make Treg cells survive and keep their functions stable. On the one hand, IL-2/STAT5 signaling pathway is essential for the development and survival of Treg cells. When IL-2 binds to its receptor, it will trigger STAT5 phosphorylation, and then activate key transcription factors, thus promoting the development and functional maturity of Treg cells (58, 59). Studies have shown that fully activating the IL-2/STAT5 signaling pathway is essential for the normal differentiation of Treg cells (60), and both IL-2 and TGF-β regulate the expression of Foxp3 through this pathway (61). Our network pharmacology predictions and Western blot results confirmed that MGEG significantly upregulated p-STAT5 levels and p-Bcl2 expression in Treg cells of AA mice. While the precise role of Bcl-2 phosphorylation can be context-dependent, recent molecular studies have established that phosphorylation at the Ser70 residue is critical for fully unlocking its anti-apoptotic potential. Phosphorylation at Ser70 actively stabilizes the Bcl-2 protein, shields it from degradation, and enhances its structural capacity to sequester pro-apoptotic mediators (62, 63). Therefore, the elevation of p-Bcl-2 (Ser70) observed in our study suggests that MGEG actively stabilizes the anti-apoptotic machinery at the post-translational level. On the other hand, Fas/FasL pathway-mediated apoptosis is a major cause of Treg reduction in AA patients (48). In the case of inflammation, Tregs with high Fas expression are susceptible to apoptosis, whereas activated Bcl-2 can resist this process (8). In addition, the external apoptosis pathway also involves the binding of FasL and Fas receptor, thus activating Caspase-8/-10 and Caspase-3/-7 and initiating apoptosis (64). Our results show that MGEG significantly reduced the expression of these pro-apoptotic proteins. Crucially, comparative analysis revealed a compelling detail: while MGEG’s inhibitory efficacy on upstream targets (such as Fas and Cleaved Caspase-8) was comparable to standard clinical regimens, it demonstrated a statistically superior inhibitory effect on the terminal executioner Cleaved Caspase-7 compared to both the IST and IST+EP therapies. This highlights that in preventing Treg apoptosis, MGEG not only matches the efficacy of standard therapies but robustly outperforms them at specific downstream nodes. Structurally, our docking results reveal that ononin deeply embeds into the death receptor FAS, while ginsenoside Rg1 securely anchors to Caspase-7, the terminal apoptotic executioner. The robust binding affinity observed between Rg1 and Caspase-7 perfectly aligns with existing pharmacological evidence, providing a structural rationale for its known capacity to shield tissue microenvironments and stem cells against excessive terminal apoptosis (65). Taken together, MGEG protects the numerical and functional integrity of Treg cells by simultaneously activating the IL-2/STAT5 survival pathway and inhibiting the Fas/FasL apoptotic cascade, thereby providing a superior stabilization of immune homeostasis.

While both MGEG and standard IST regimens ultimately suppress Th1 overactivation and restore the naïve/memory T cell balance, their fundamental modes of action differ significantly. Conventional IST relies on intense, non-specific T cell depletion (ATG) or calcineurin blockade (CsA), which abruptly shuts down immune activity in a broad and indiscriminate manner (4). This blunt suppression frequently leads to immune exhaustion, severe toxicities, and high relapse rates (4, 5). Notably, CsA-mediated calcineurin inhibition has been demonstrated to impair NFAT-dependent Foxp3 gene transcription, which inherently constrains Treg restoration even as effector T cell activity is suppressed (66). This mechanistic limitation is consistent with our observation that IST monotherapy achieved significantly inferior Foxp3 upregulation compared to MGEG (*P* < 0.05). In contrast, our data characterize MGEG as an active immune tolerance reprogrammer rather than a broad immunosuppressant. This distinction is evidenced by three distinct molecular advantages. First, MGEG demonstrated significantly superior Foxp3 upregulation compared to both IST and IST+EP, suggesting active transcriptional restoration of the Treg lineage master regulator—an effect structurally supported by Magnoflorine’s direct engagement with the Foxp3 protein in our docking analysis (−8.9 kcal/mol). Second, MGEG uniquely suppressed the terminal apoptotic executioner cleaved Caspase-7 to a significantly greater degree than both standard regimens. This reflects targeted Treg cytoprotection at the effector level, corroborated by the direct molecular interaction between Ginsenoside Rg1 and Caspase-7 (−8.9 kcal/mol). Third, while IST’s partial restoration of miR-146a likely reflects an indirect consequence of reduced inflammatory burden following lymphodepletion, MGEG achieved significantly higher miR-146a upregulation than IST monotherapy (*P* < 0.01), consistent with an active, compound-driven transcriptional mechanism as hypothesized above. Together, this multi-component, mechanistically coordinated action simultaneously secures Treg survival pathways and restores the miR-146a negative feedback loop. Consequently, while MGEG achieved phenotypic recovery in the naïve/memory T cell balance comparable to IST monotherapy (13), it accomplishes this through an active reprogramming strategy rather than broad lymphodepletion, thereby preserving long-term immune reserve and avoiding the cellular cytotoxicity inherent to conventional therapies.

Furthermore, MGEG’s reliable safety profile stems directly from its constituent formulas, GEG and DBT. Human clinical trials confirm GEG avoids renal and hepatic detriment during prolonged use (67). Moreover, DBT and its primary active compounds, such as Astragaloside IV and Ginsenoside Rg1, possess documented hepatoprotective and antioxidant properties rather than systemic toxicity (68, 69). This robust pharmacological foundation completely aligns with our *in vivo* findings, where treated mice maintained healthy physiological and organ conditions without structural liver damage.

The clinical relevance of our findings rests on a dual foundation of phenotypic alignment and structural compatibility. We specifically selected the BALB/c model because it accurately phenocopies the distinct immune disruptions of human AA, namely Treg deficiency and Th1-driven bone marrow failure, thereby anchoring our physiological observations to the human disease context. Simultaneously, our molecular simulations confirmed that active MGEG compounds, such as Magnoflorine and Ginsenoside Rg1, interact robustly with human structural variants of essential targets like RORγ, Foxp3, and STAT1. With binding energies consistently surpassing the -7.0 kcal/mol threshold, this computational data reveals a profound molecular synergy with human immune networks. Ultimately, matching this multi-tiered evidence against the established success of MGEG in clinical AA management confirms that modulating the miR-146a/STAT1/SOCS1 pathway is a highly realistic and translatable strategy for human therapeutic intervention.

## 5 Conclusion

Our research shows that MGEG can effectively improve the problem of bone marrow failure in AA mice. Its mechanism involves a multi-level immunoregulation network: centrally, MGEG upregulates miR-146a to specifically target and inhibit the STAT1/SOCS1 signaling axis, thus preventing the immune damage caused by Th1 cells from the source. At the same time, it can also regulate the expression of Fas/FasL and IL-2/STAT5 pathway-related proteins, reduce the apoptosis of Treg cells, and enhance the expression of Foxp3, thus maintaining the stability and function of Treg cells. In addition, MGEG can correct the imbalance between initial T cells and memory T cells, alleviate immune aging and protect the immune reserve function of the body. These findings provide a solid theoretical basis for MGEG as a multi-target immunomodulator in the treatment of aplastic anemia.

## Data Availability

The raw data supporting the conclusions of this article will be made available by the authors, without undue reservation.
