# Supplementary material for: Modified Guilu Erxian Glue restores immune tolerance in aplastic anemia by reprogramming T cell differentiation via the miR-146a/STAT1/SOCS1 axis
Source: Front Immunol. 2026 May 29;17:1826665. doi: 10.3389/fimmu.2026.1826665 (PMC13259725; doi:10.3389/fimmu.2026.1826665)

Supplementary Material

# Supplementary Data

Western blot

T-bet


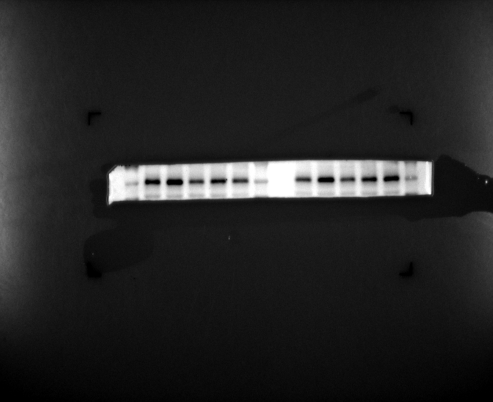

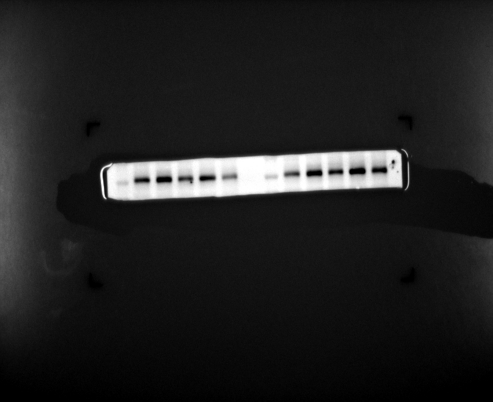


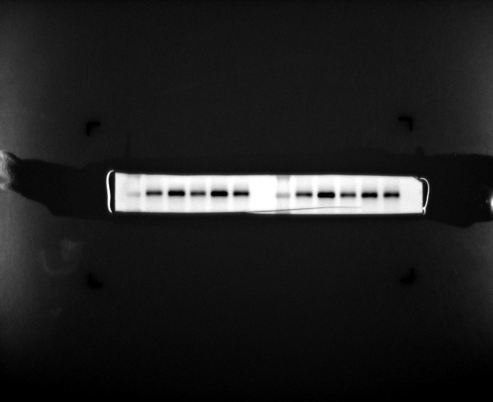


GADPH


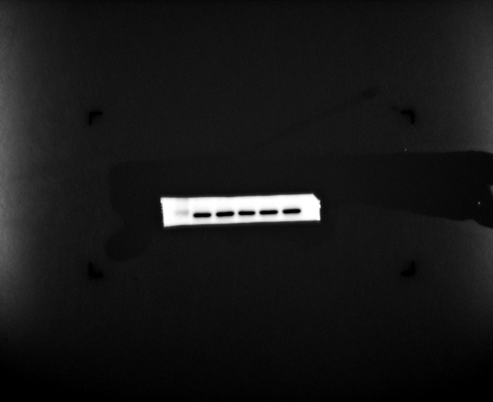

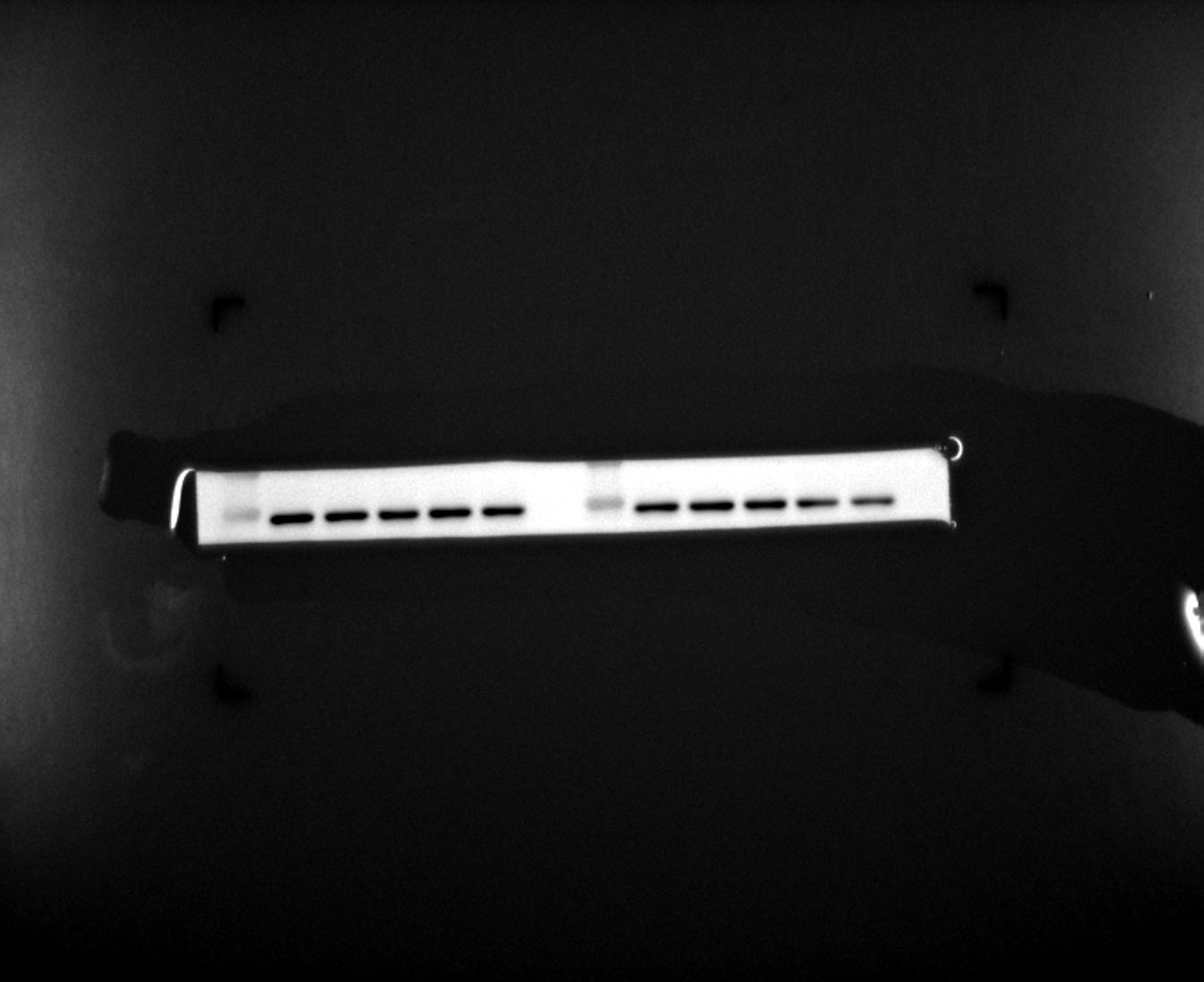


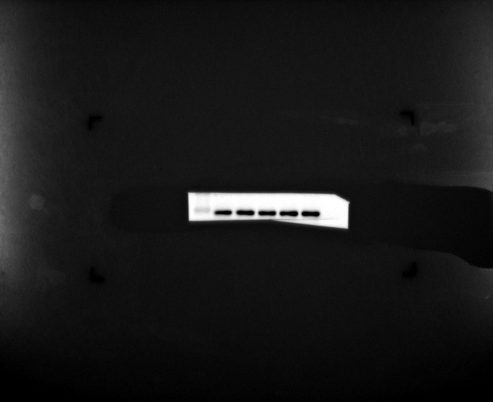


GATA3


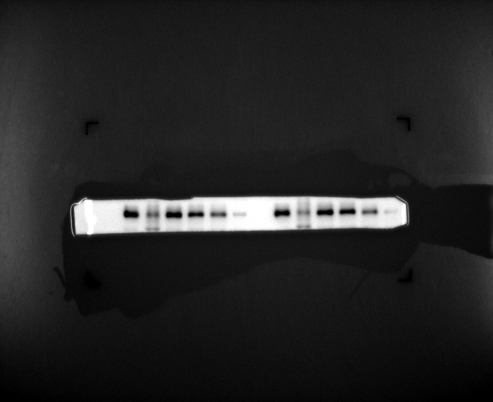

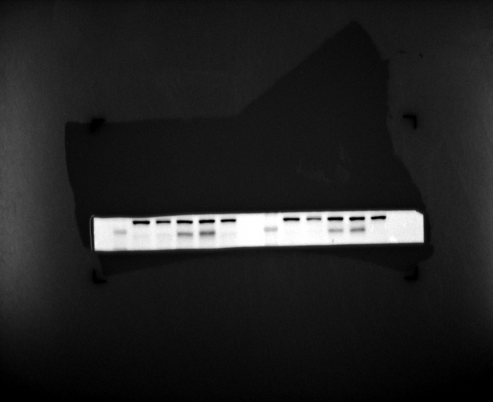


GADPH


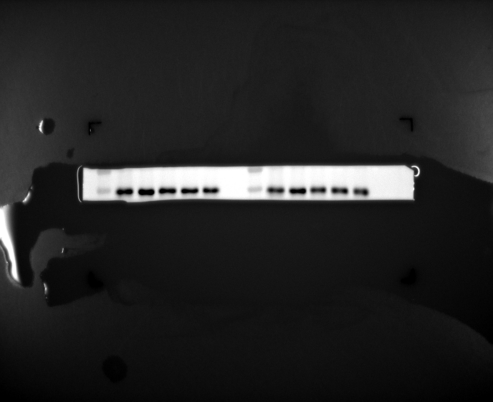

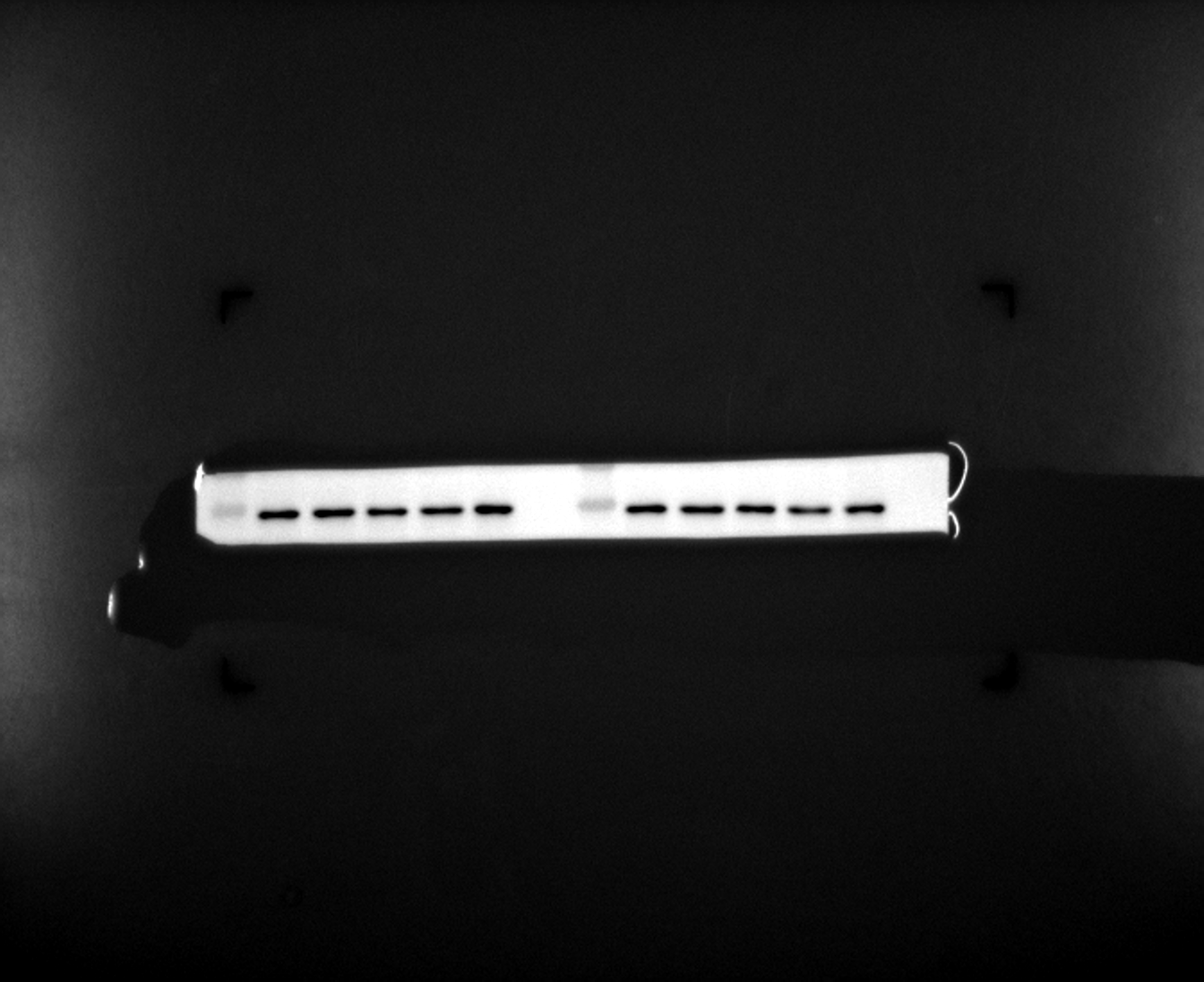


RORγ


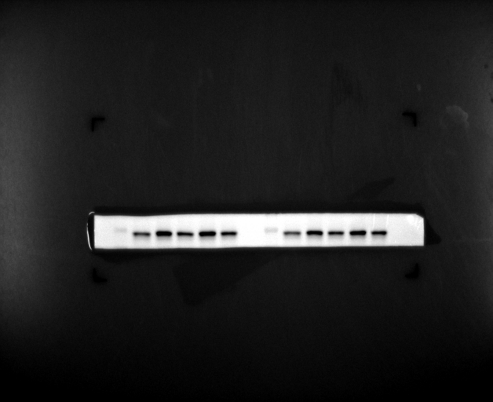

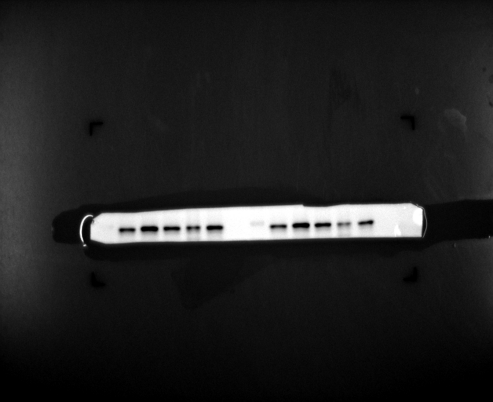


GADPH


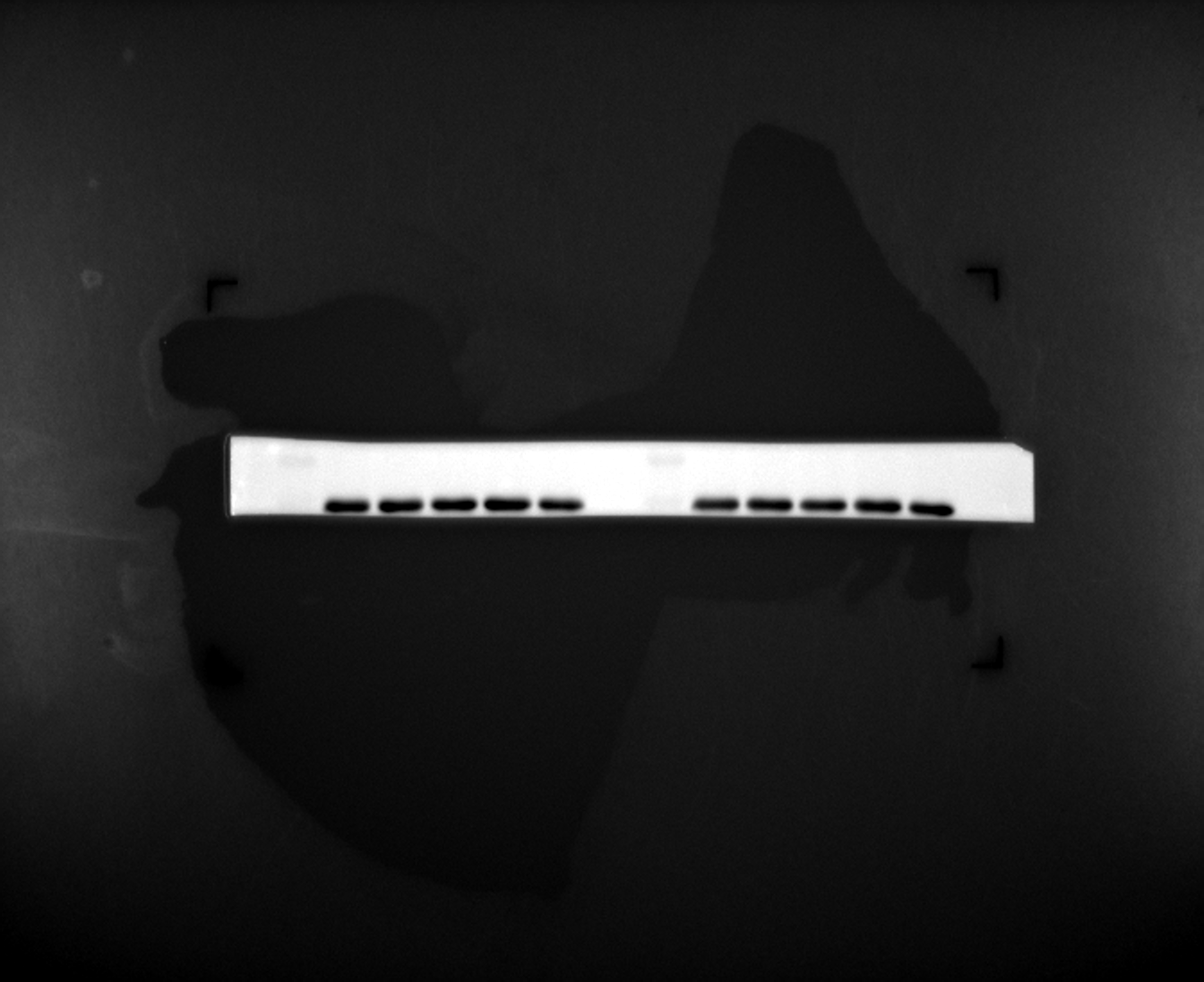

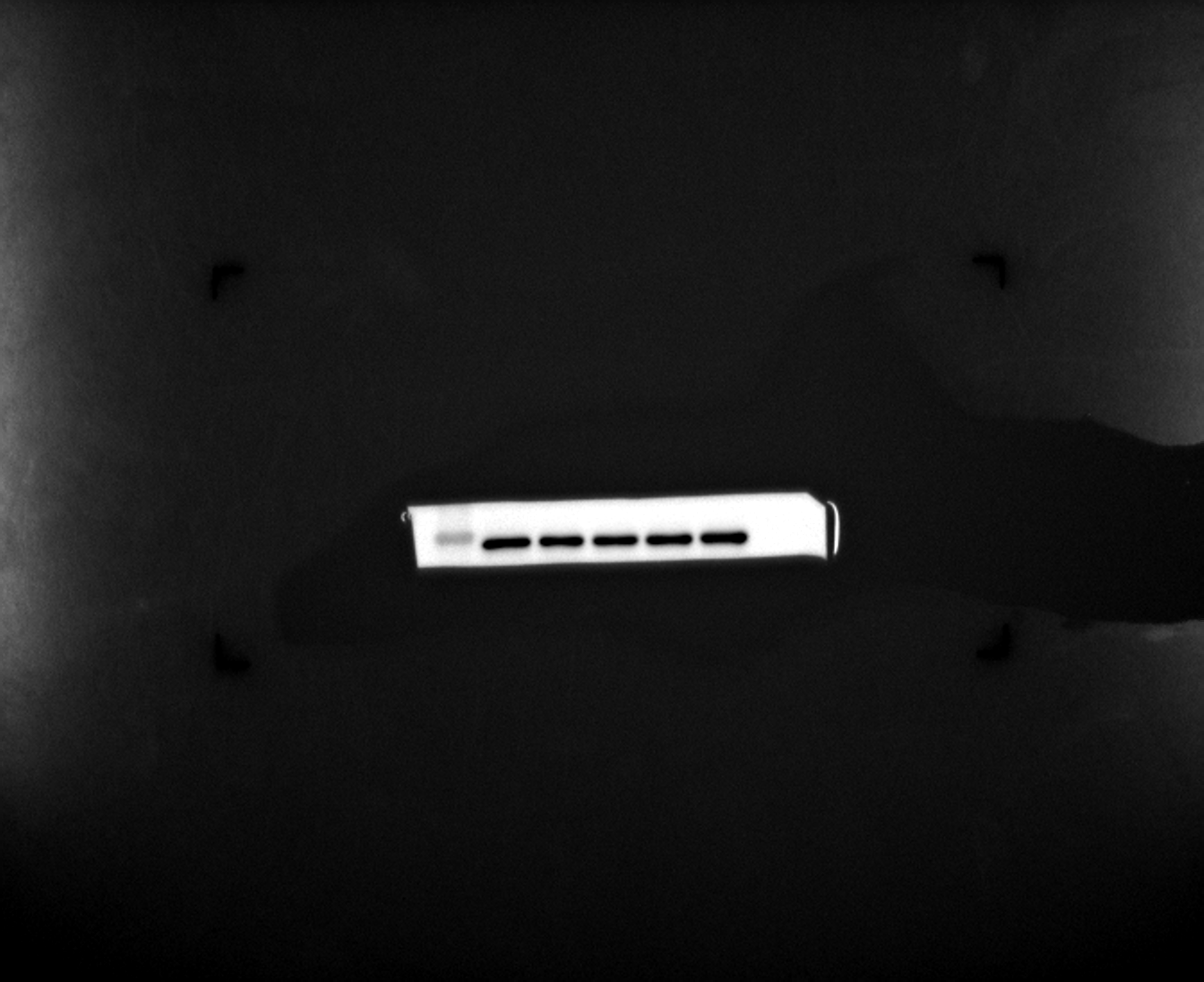


FOXP3


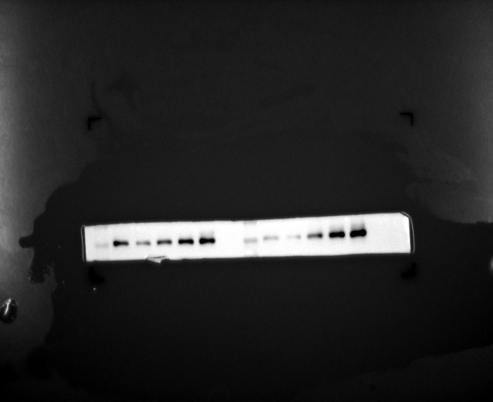

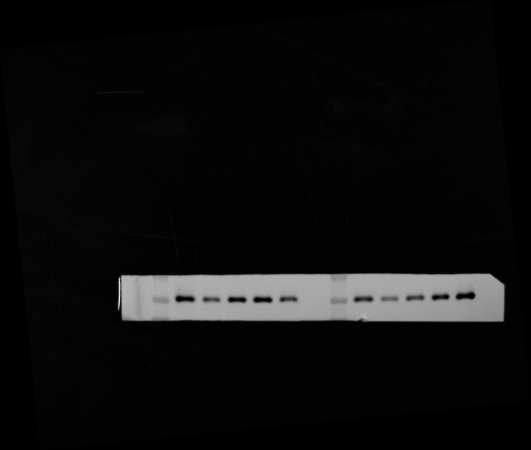


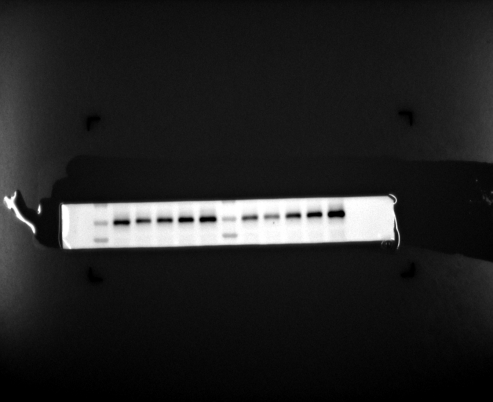


GADPH


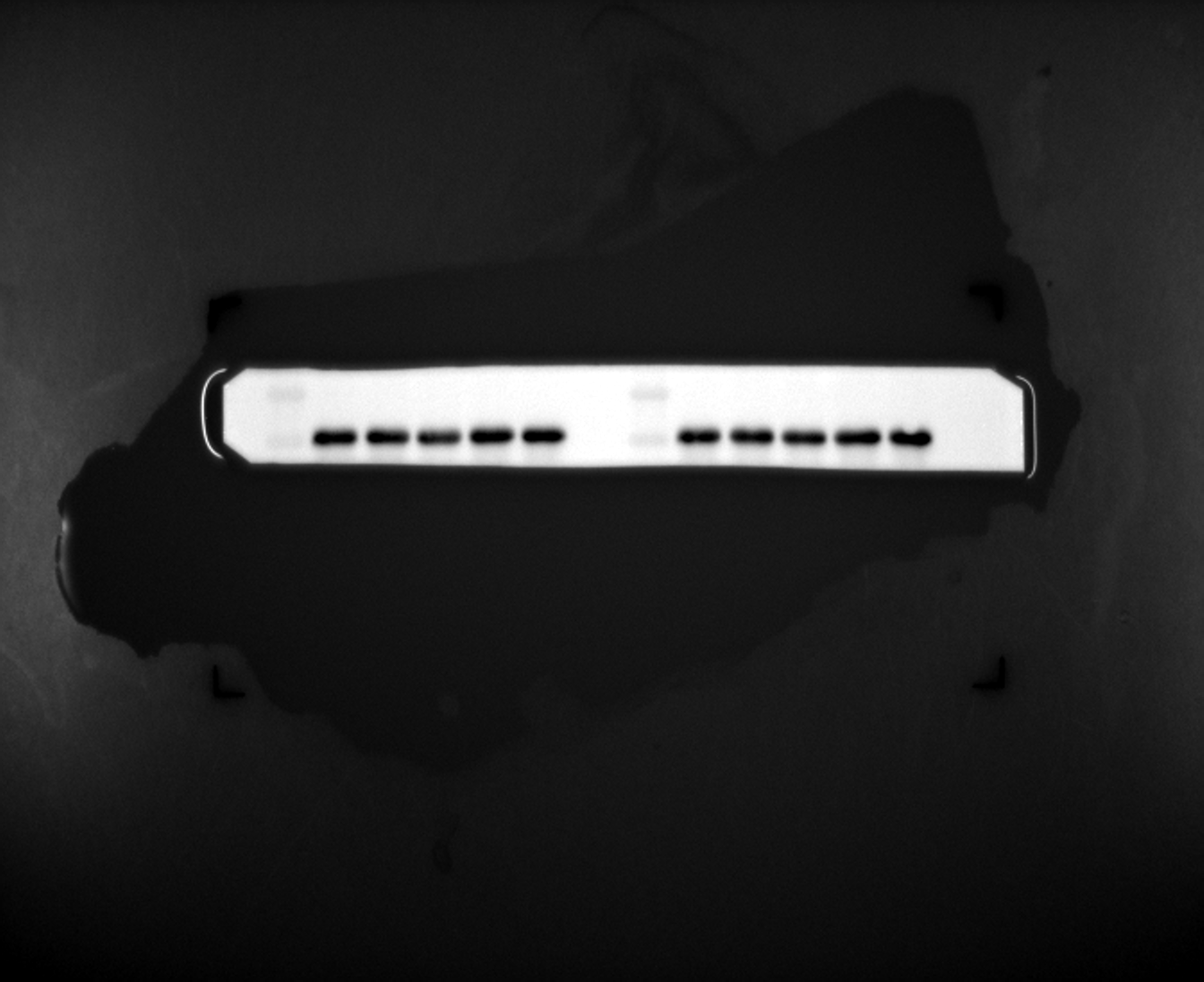

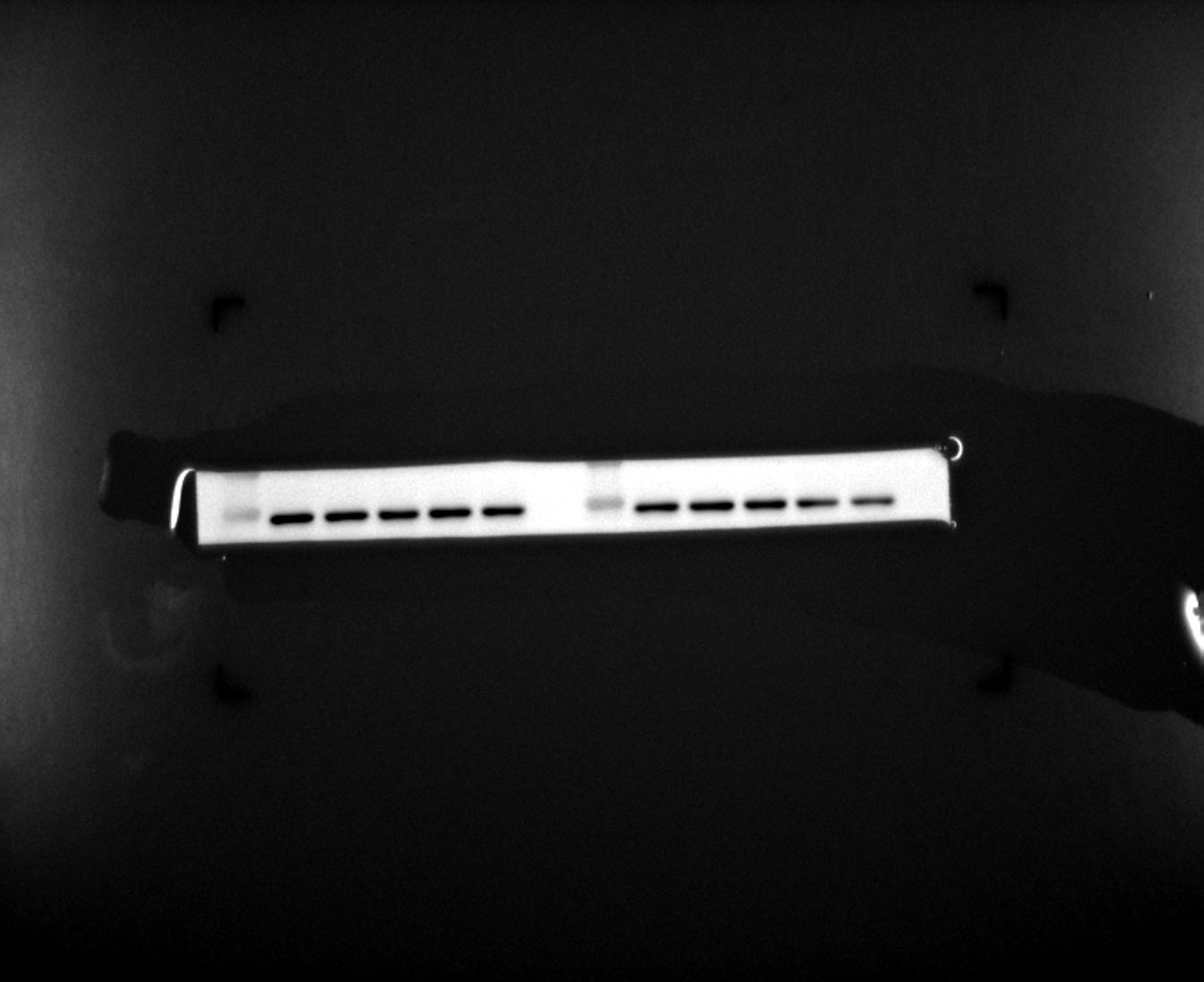


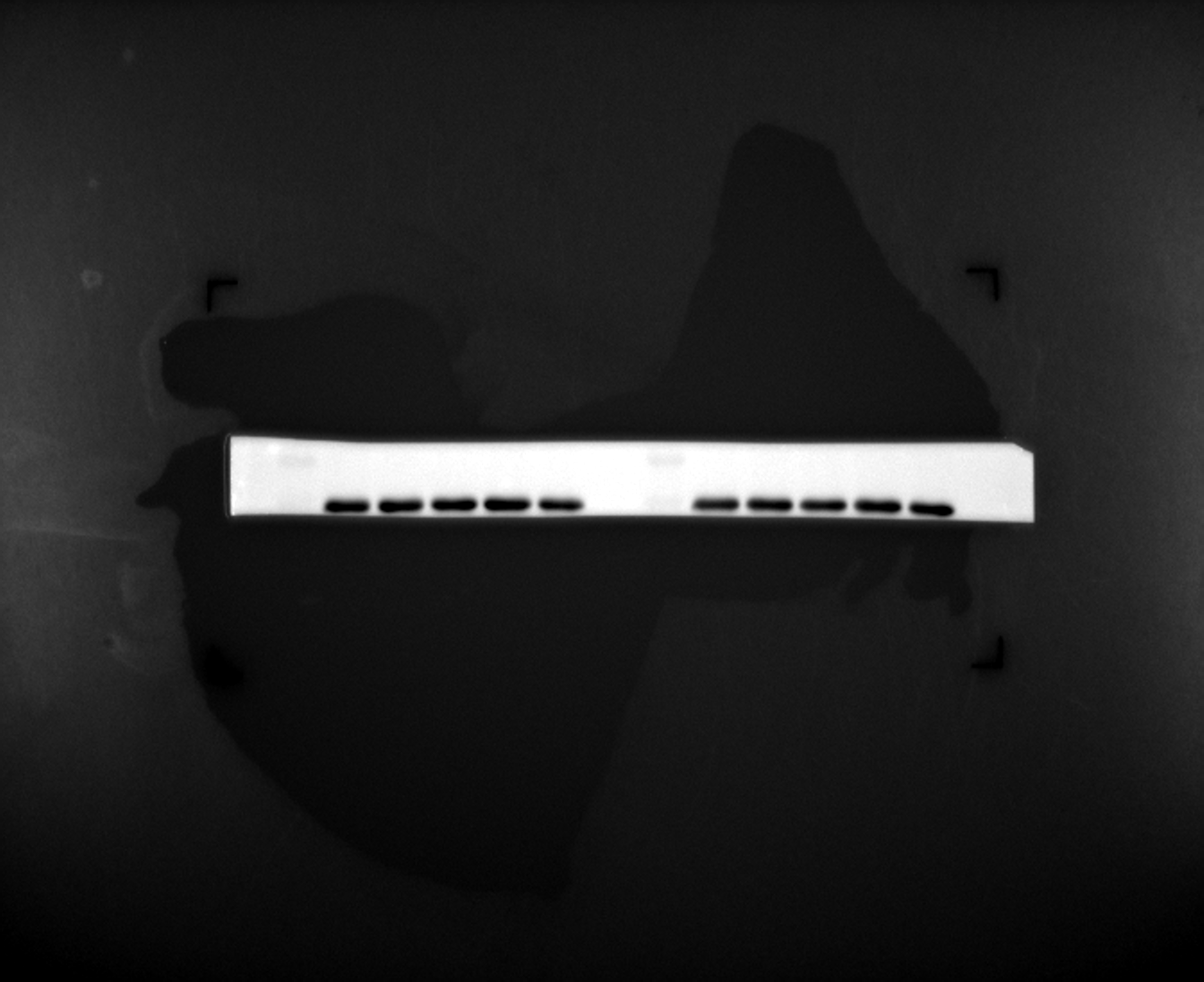


P-STAT1


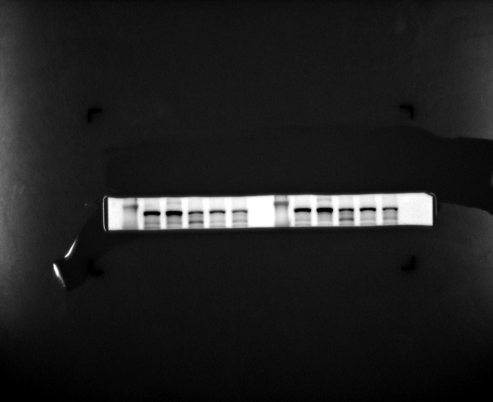

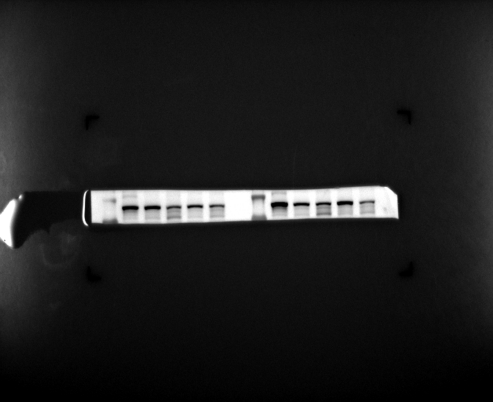


STAT1


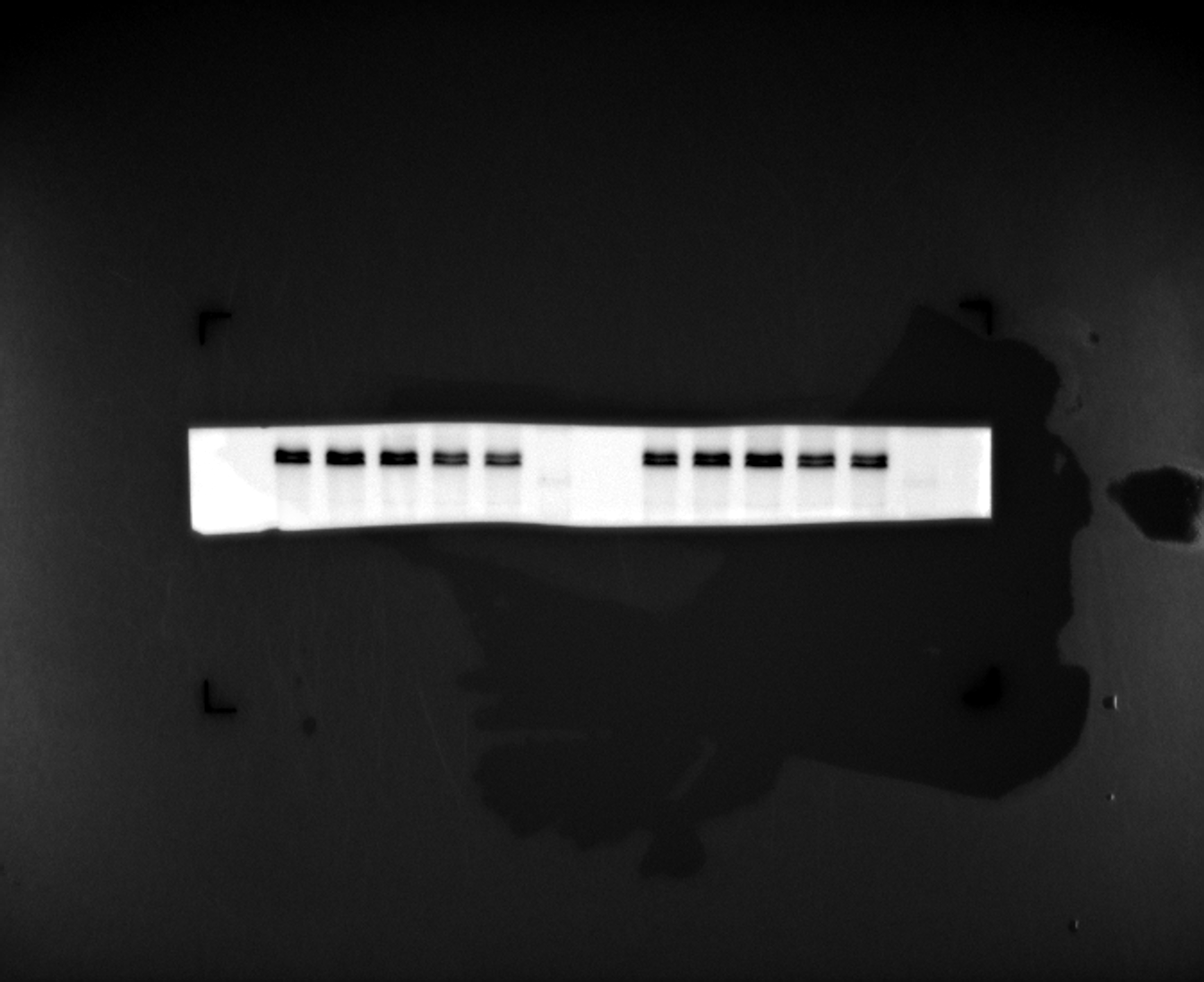

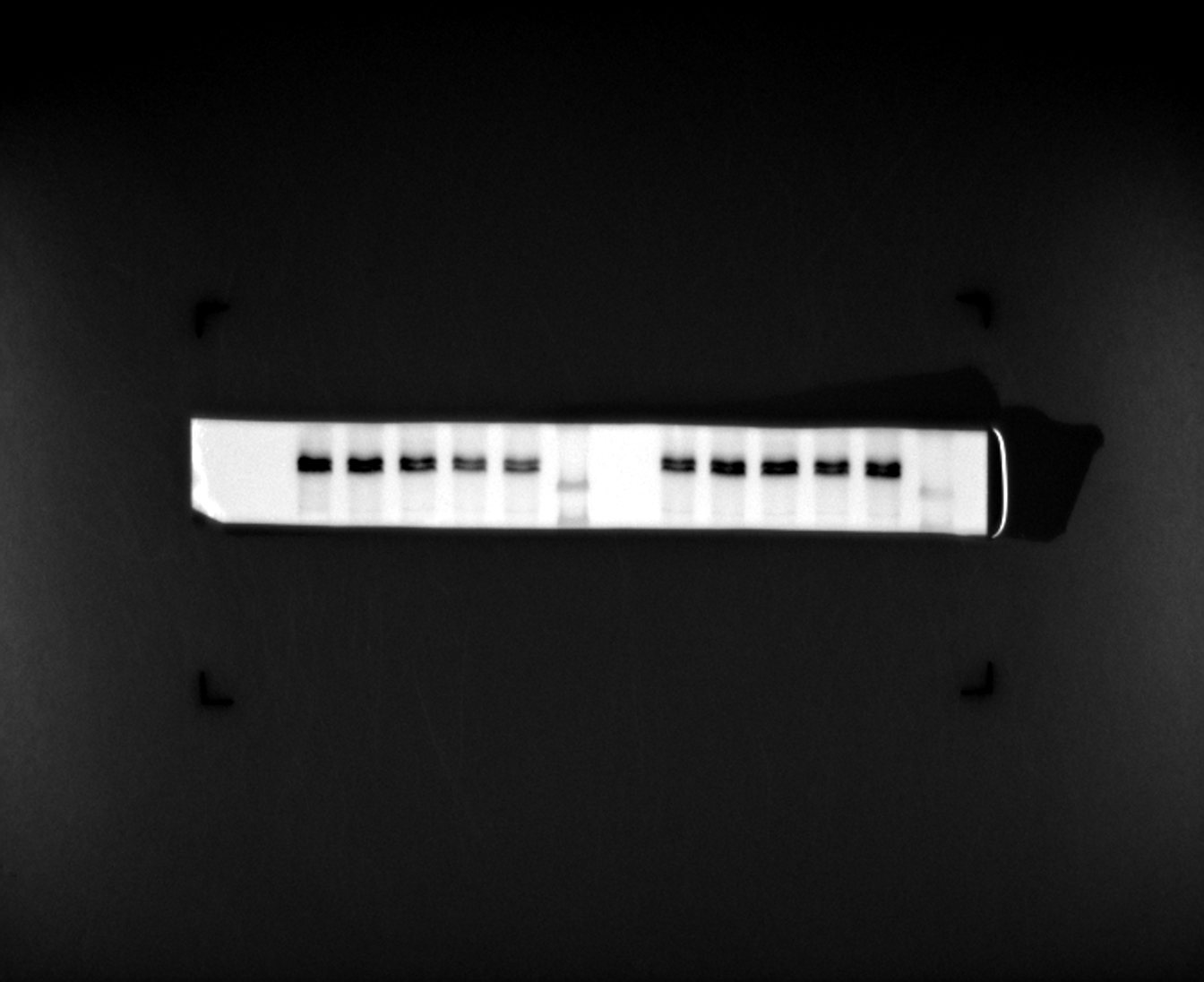


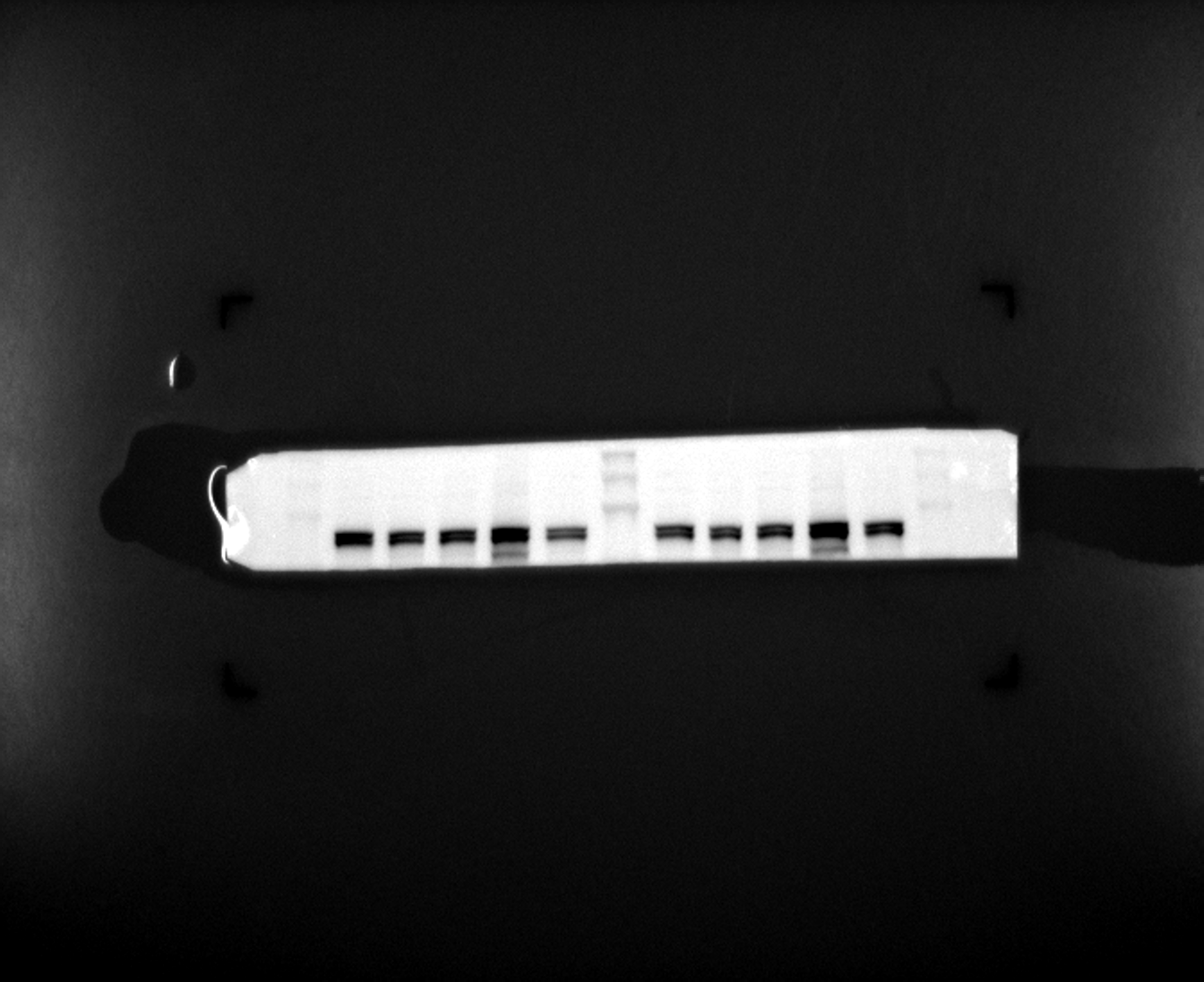


STAT1


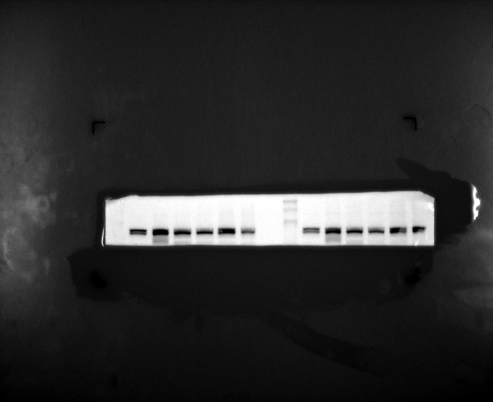

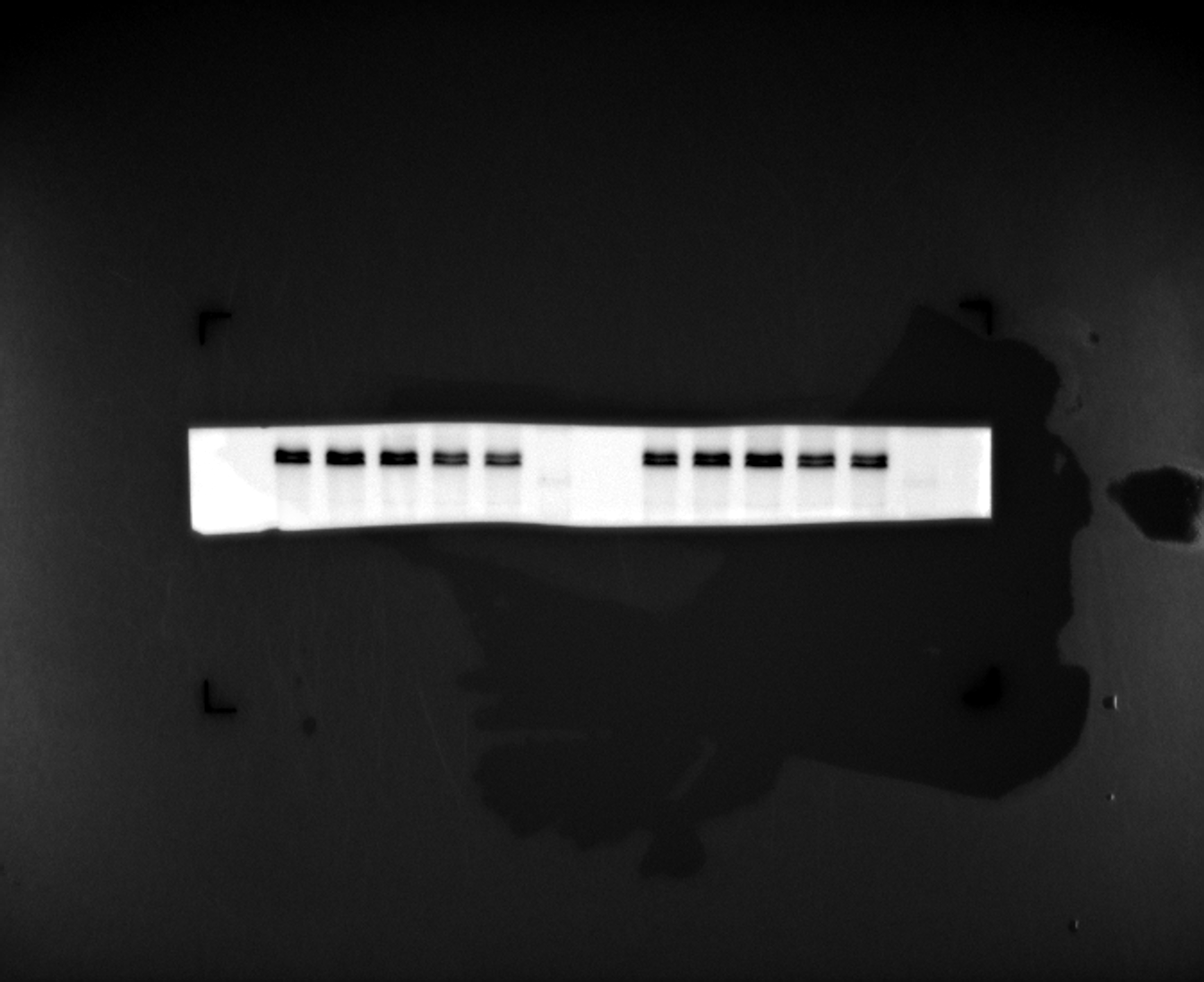


GADPH


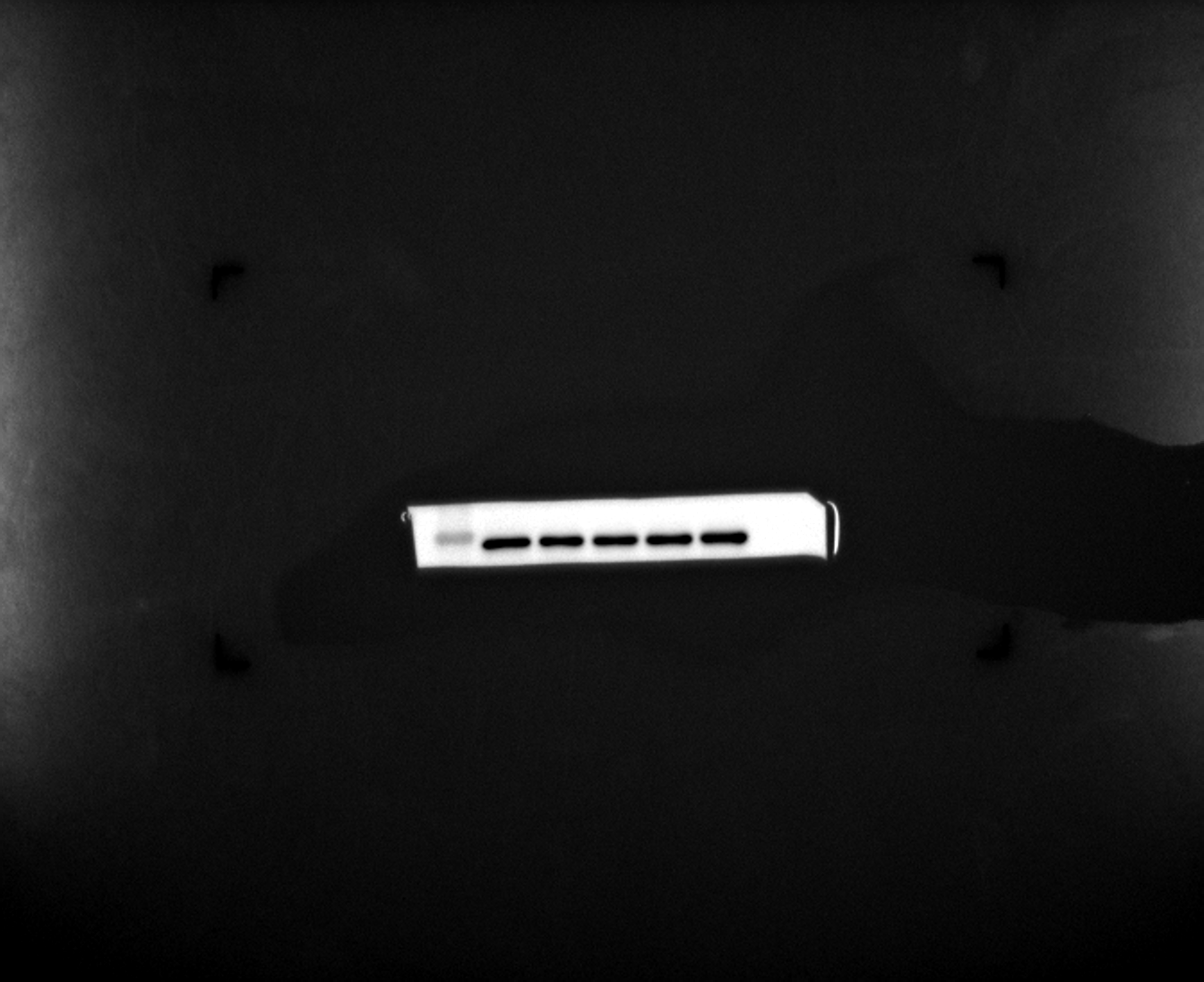

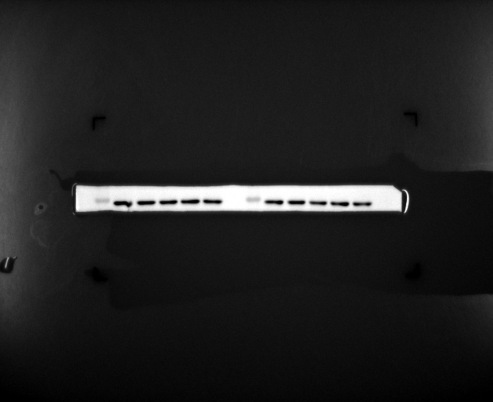


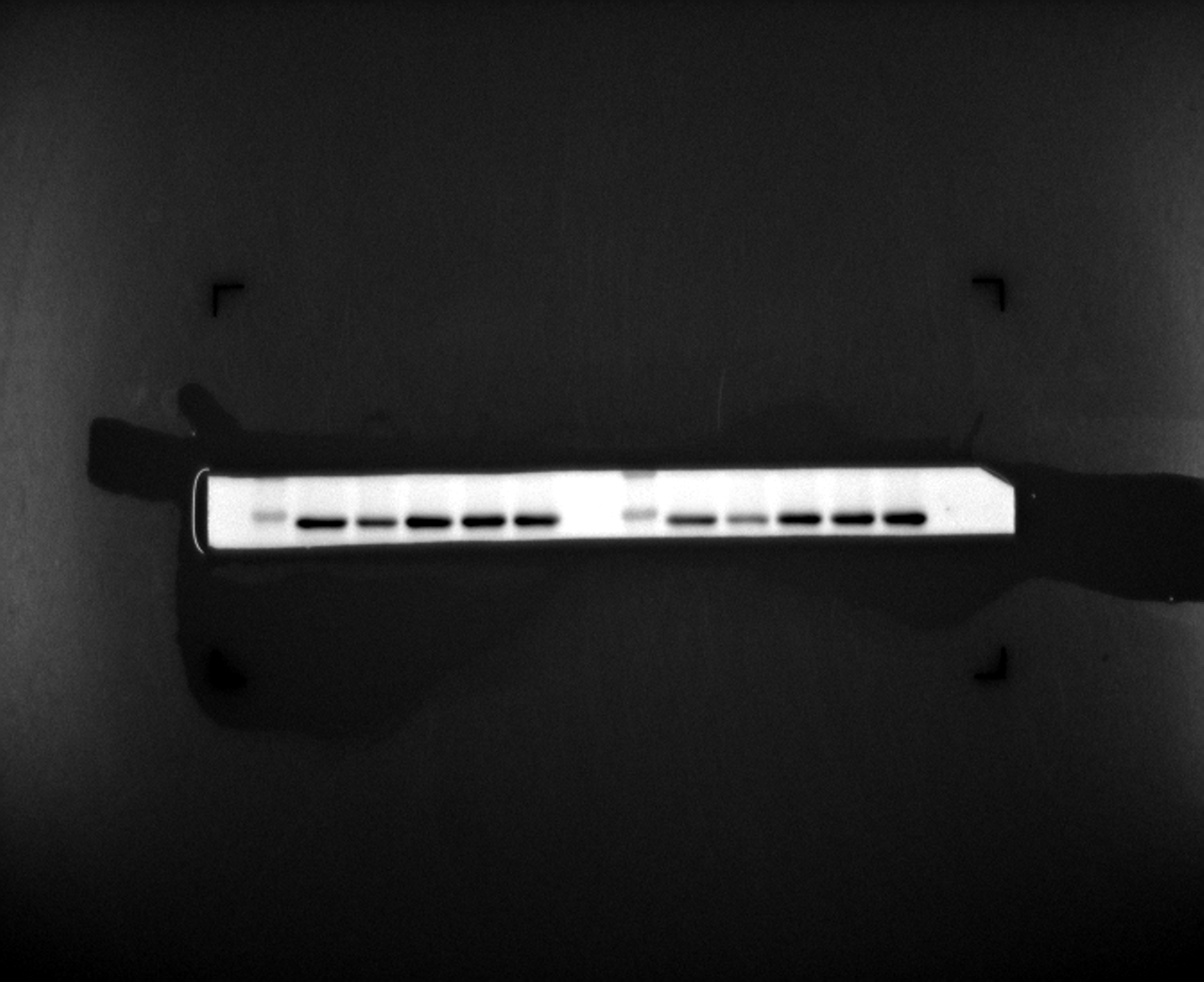


SOCS1


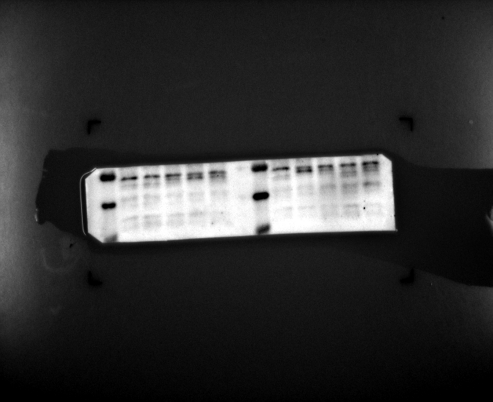

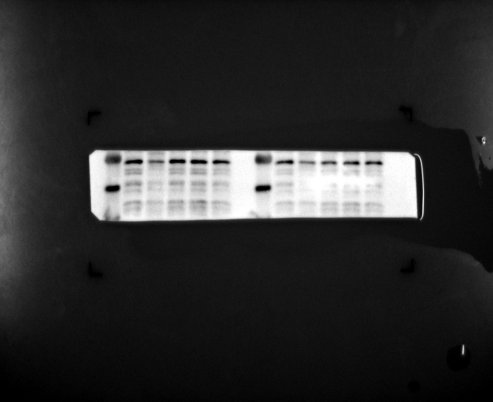


GADPH


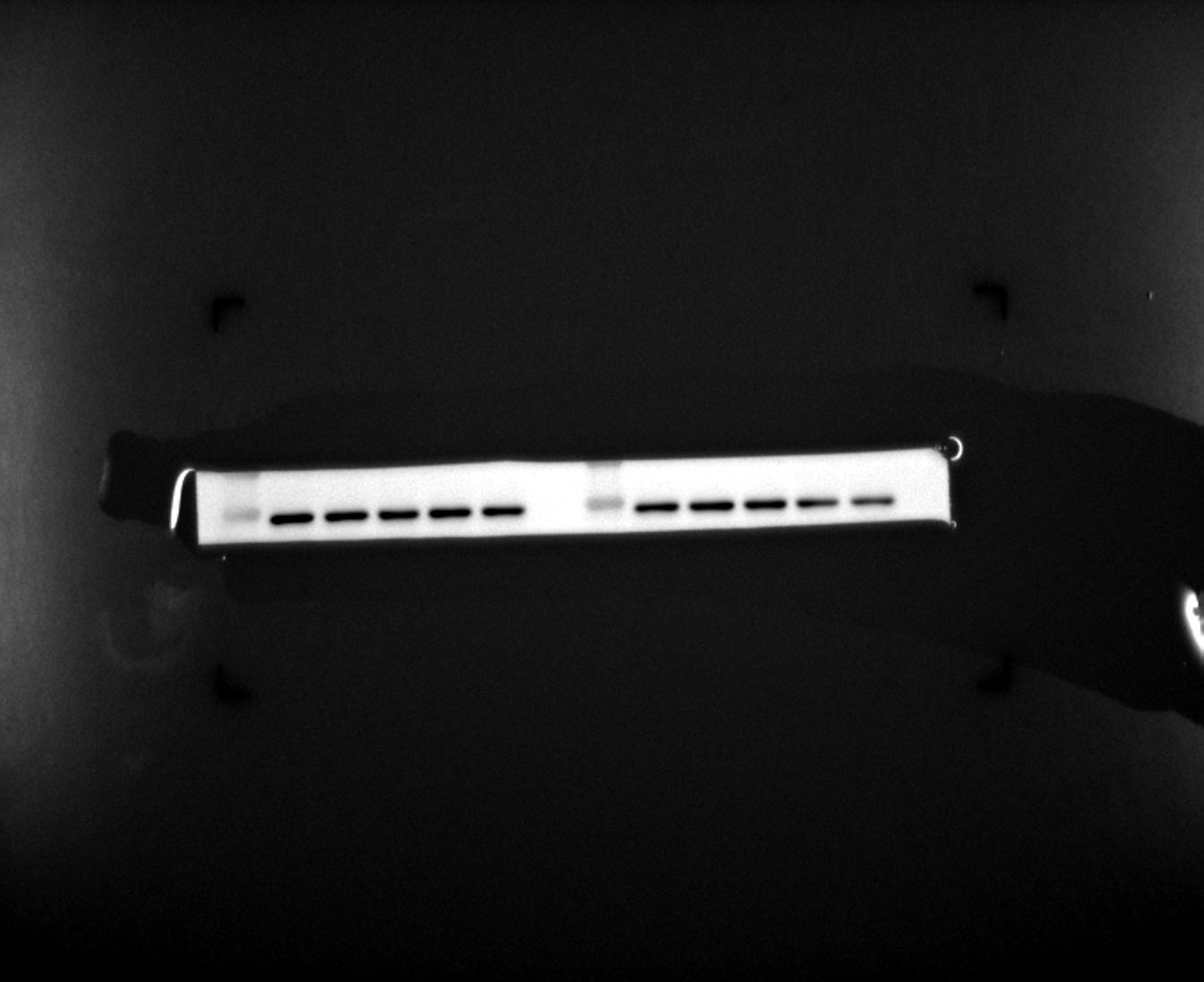

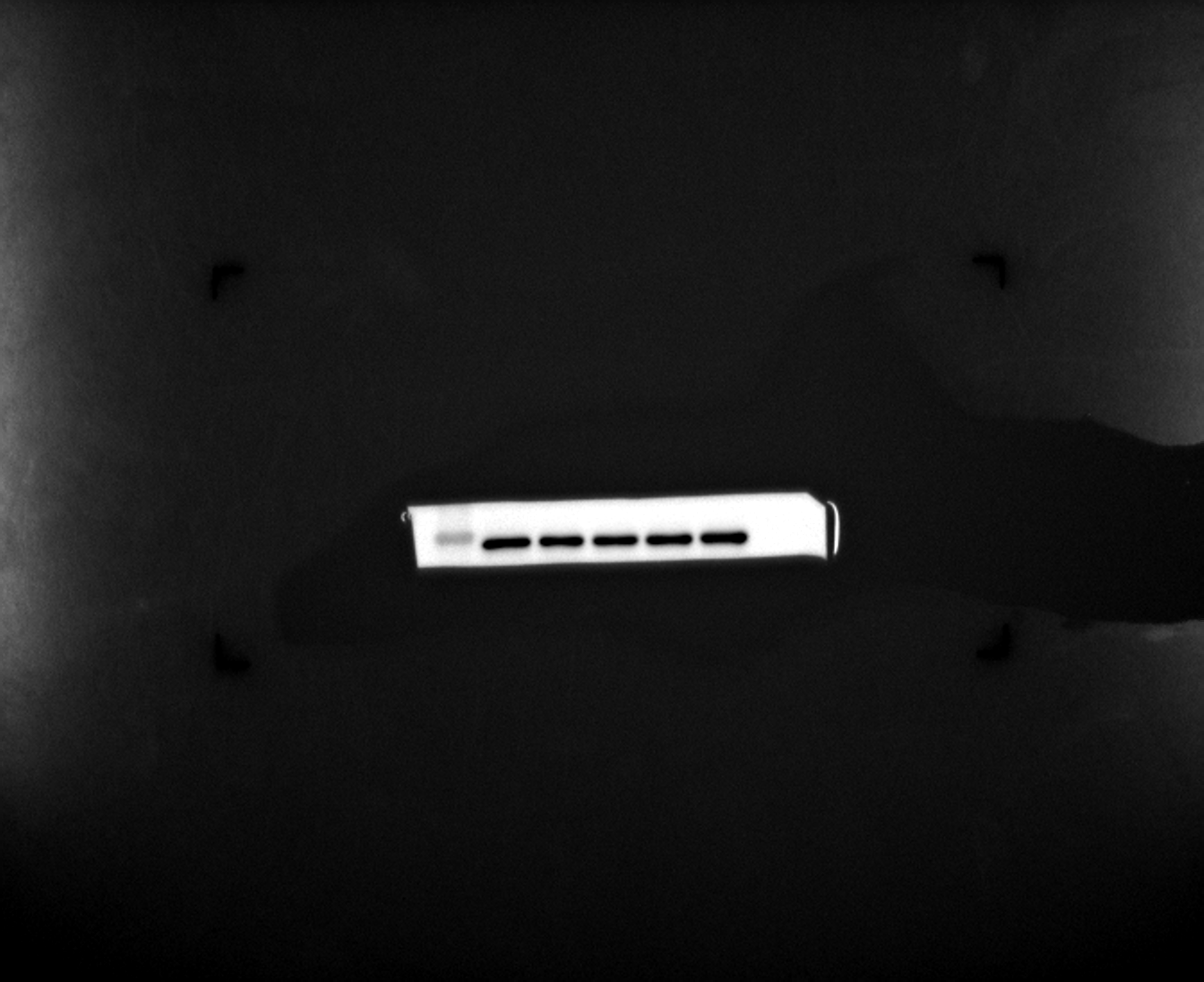


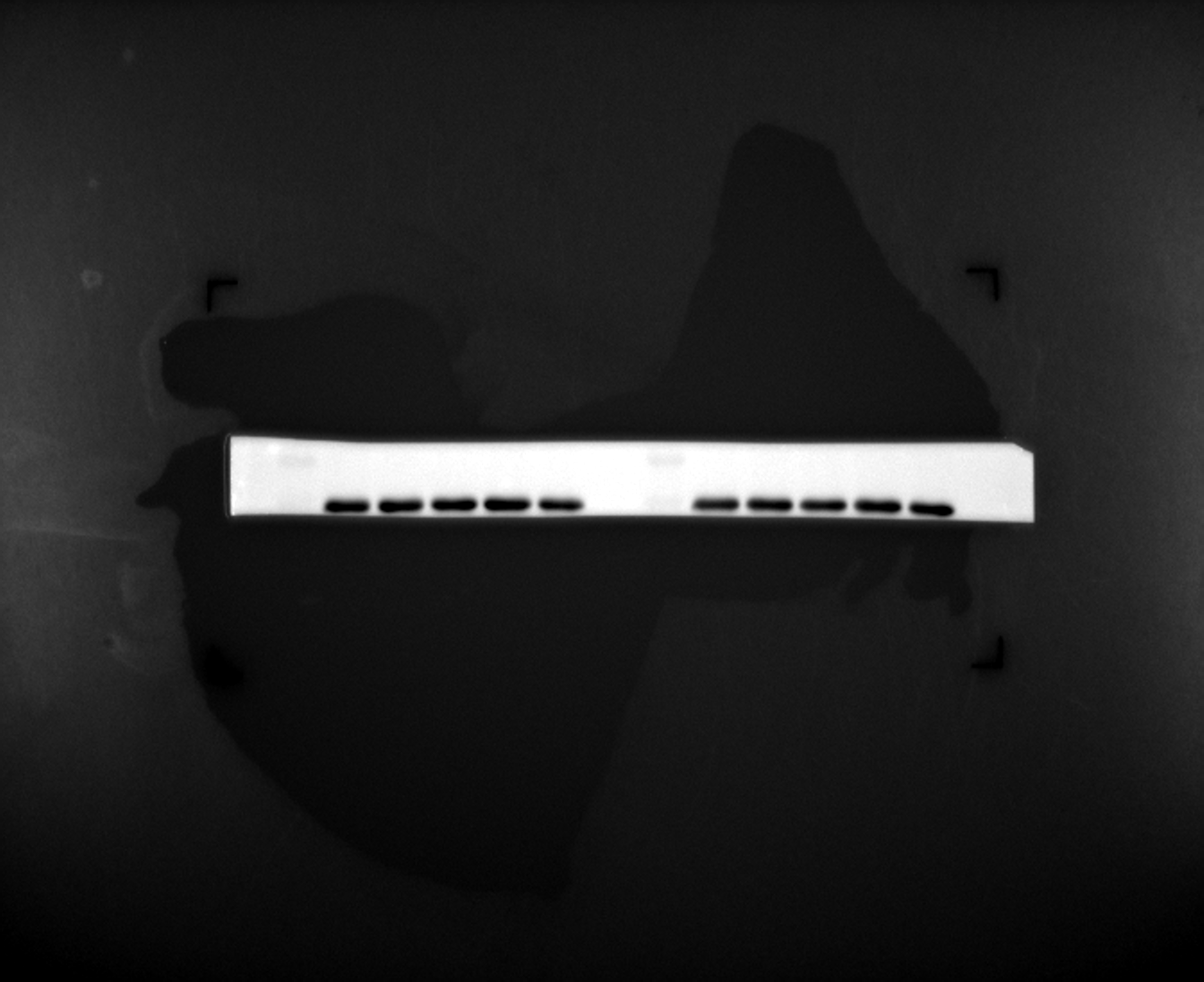


FAS


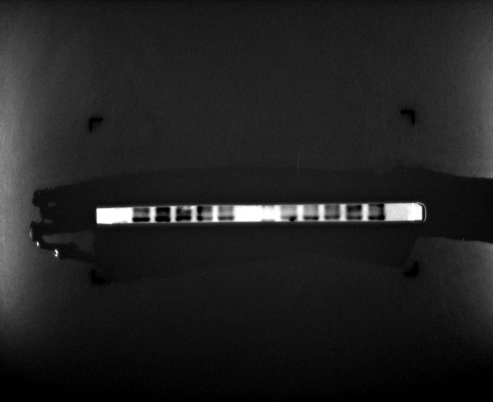

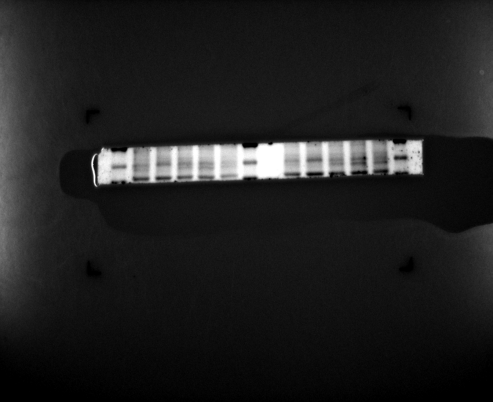


11


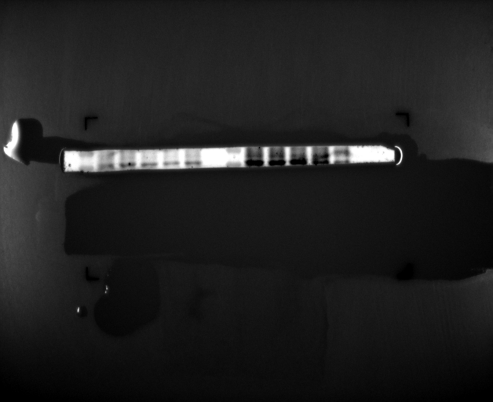


GADPH


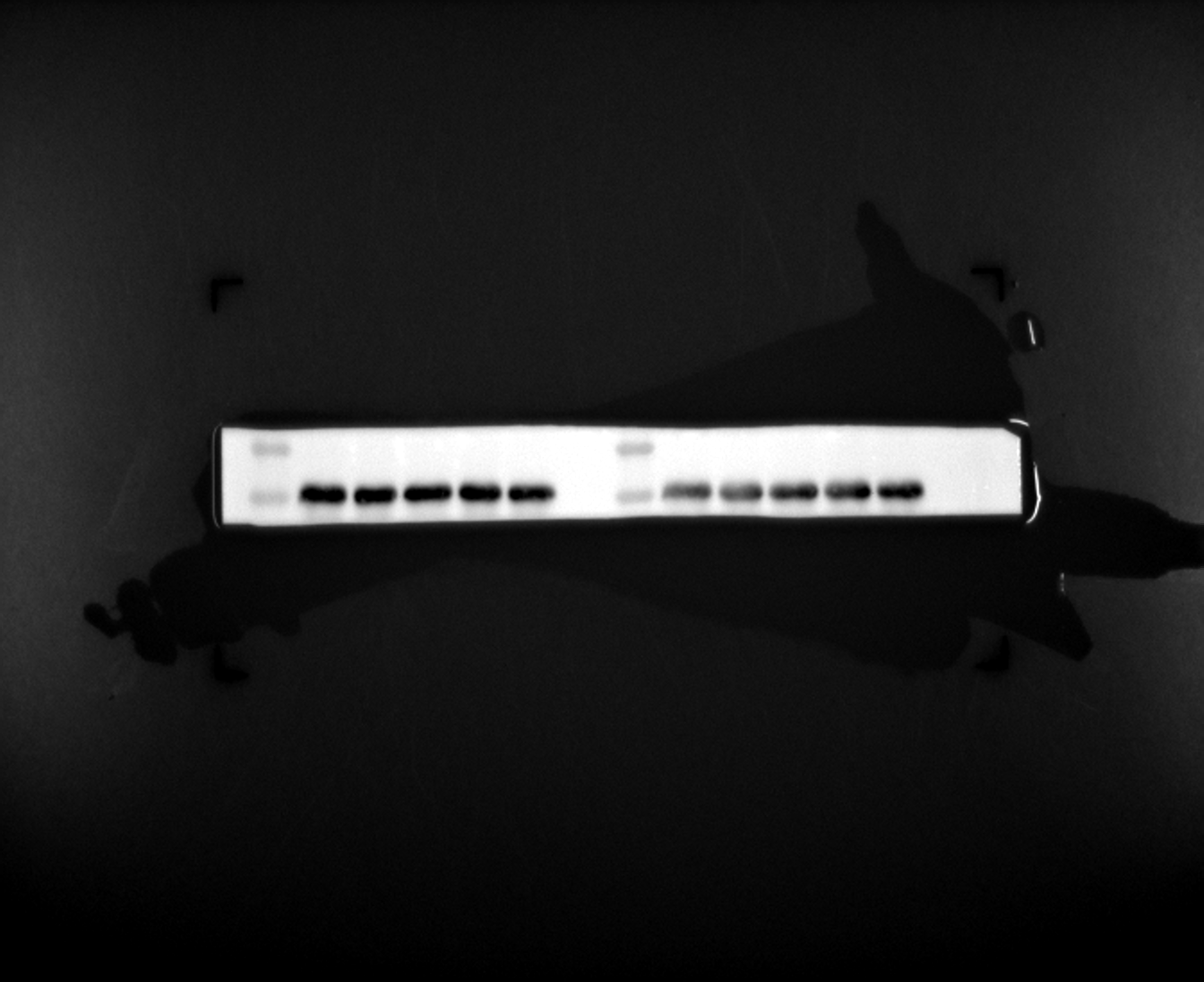

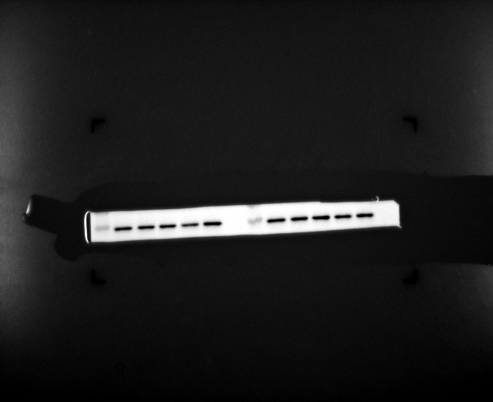


Caspas7


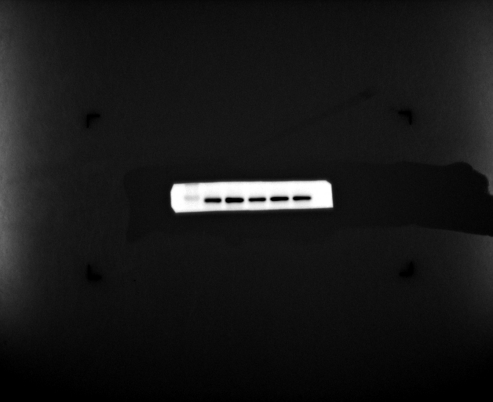

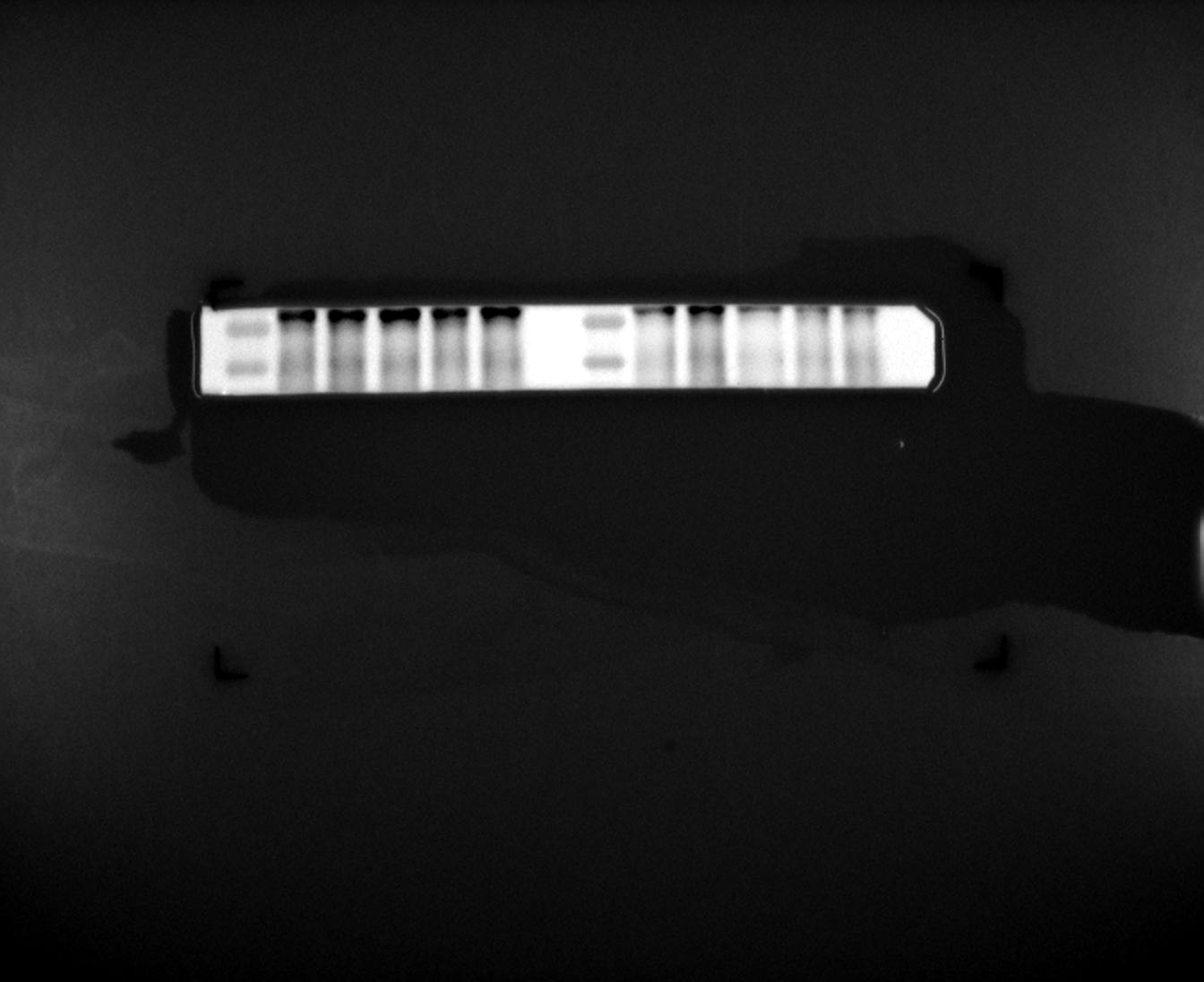


GADPH


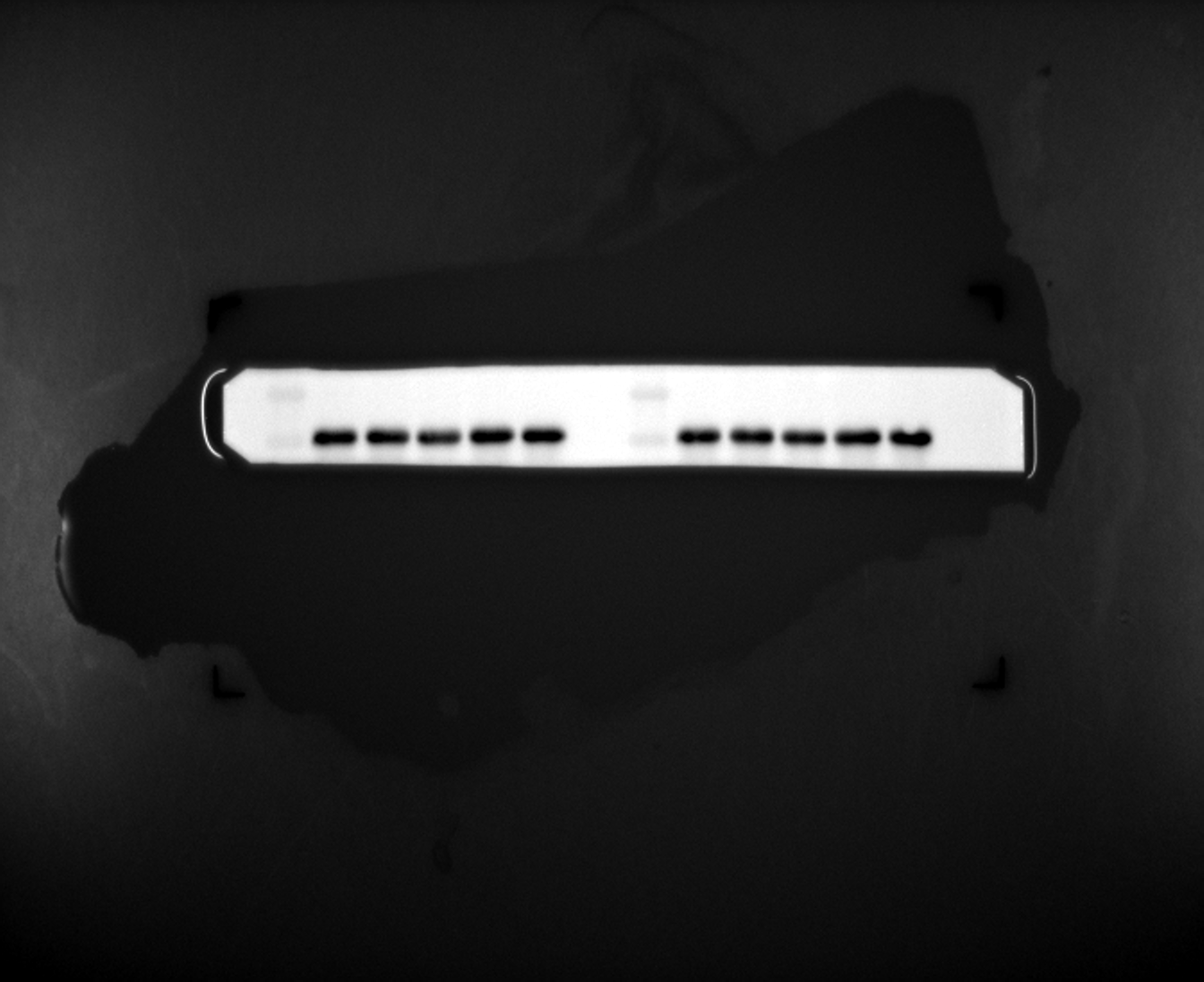

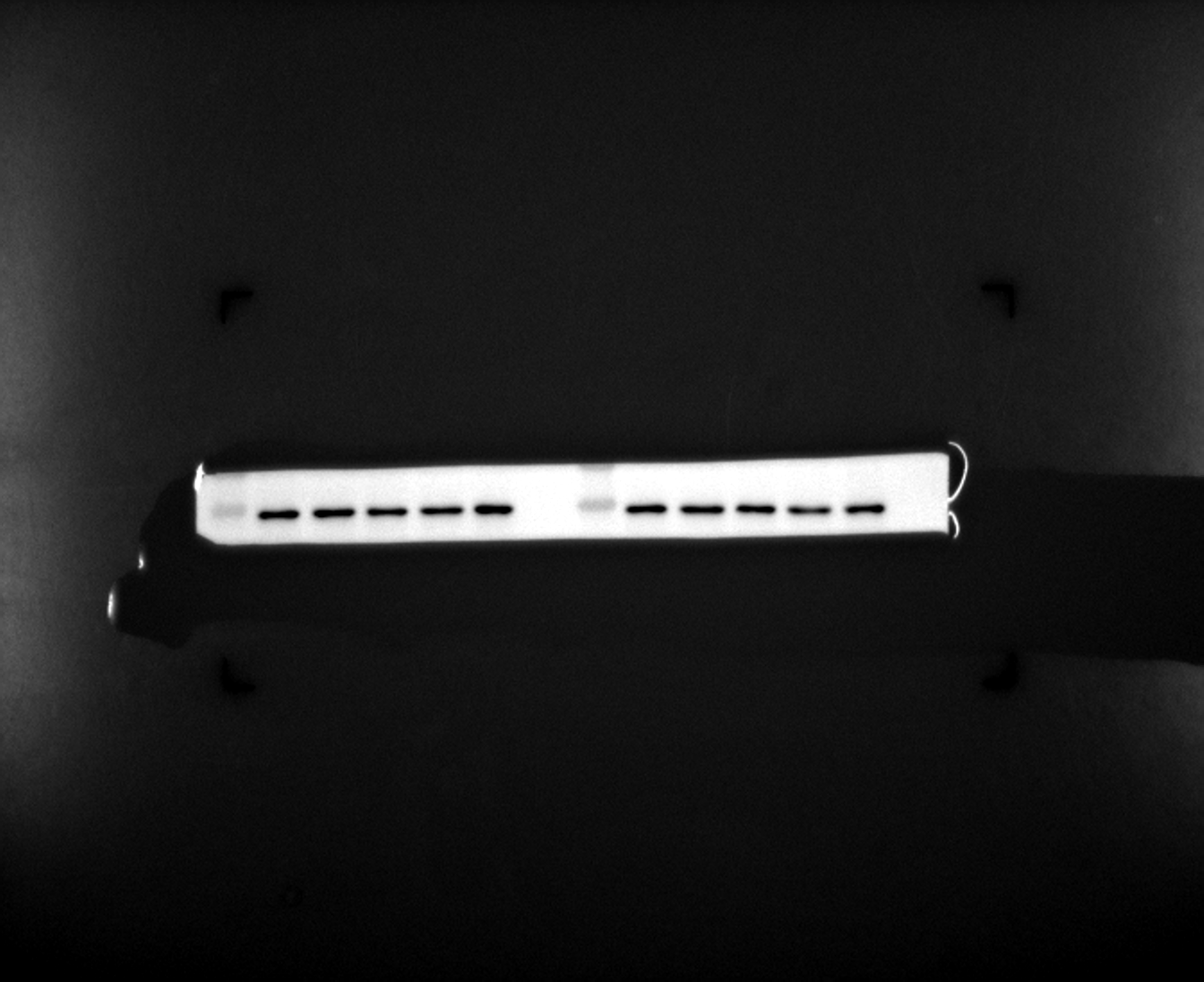


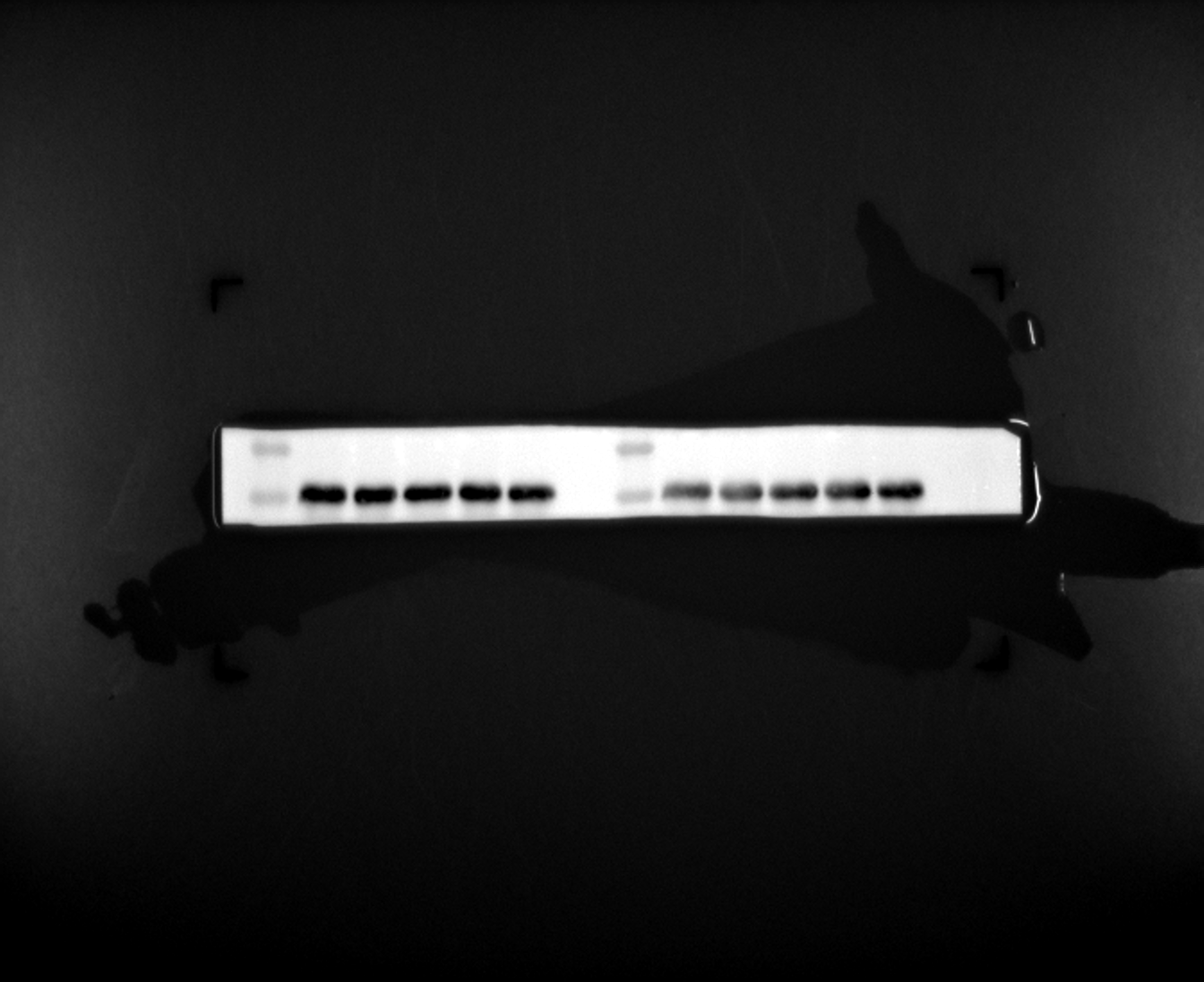


Cleaved Caspas7


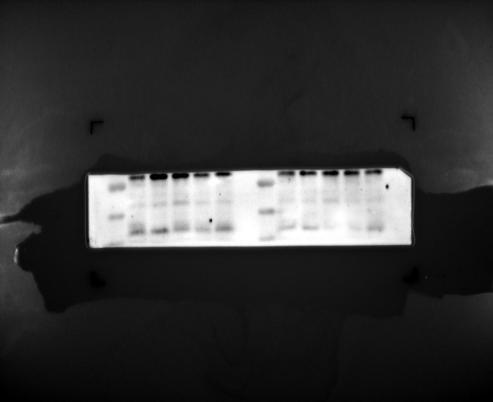

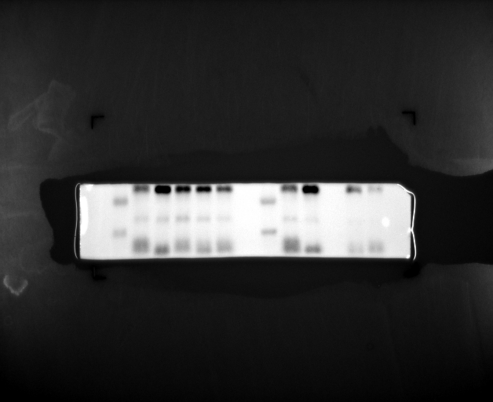


Caspase7


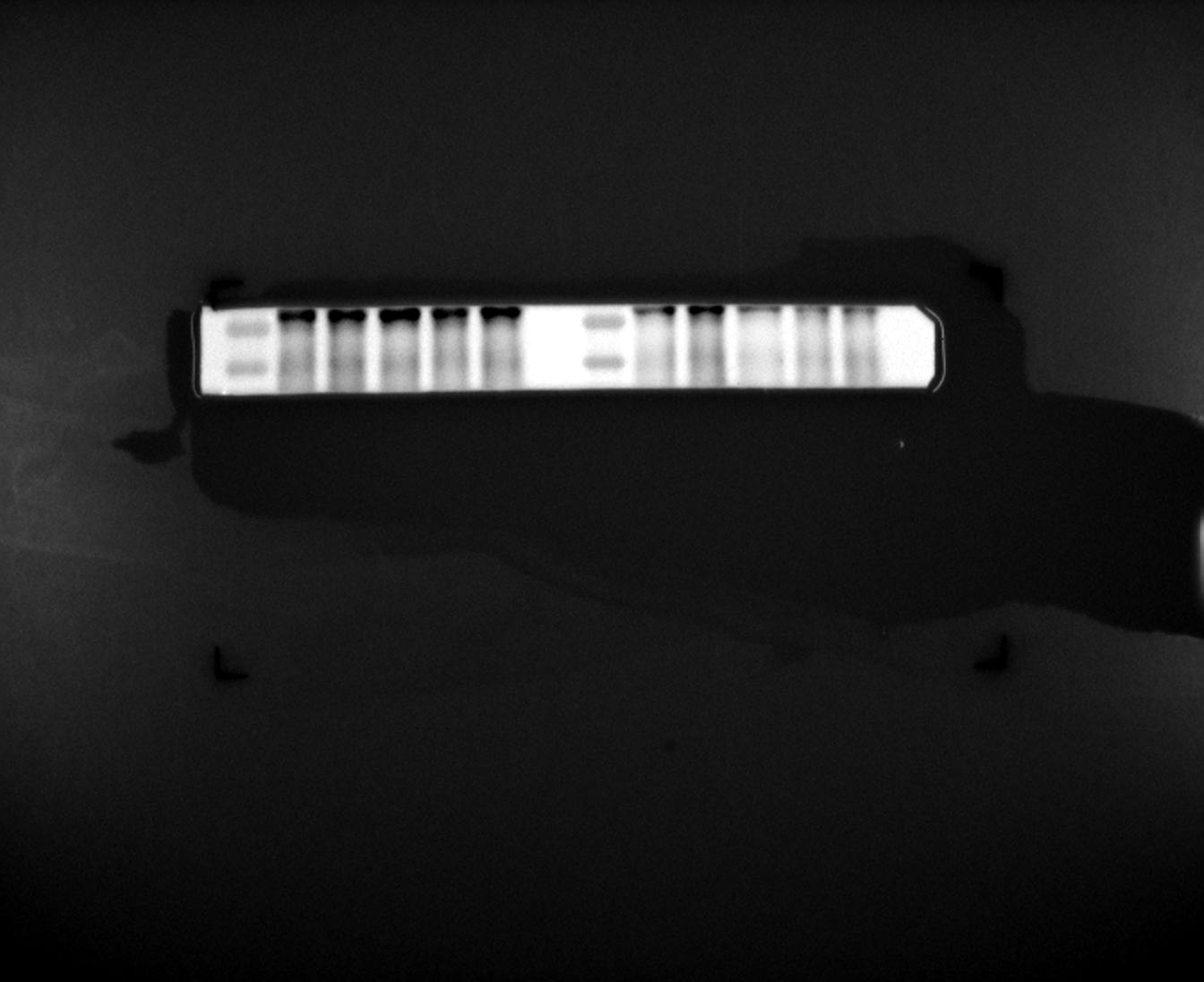

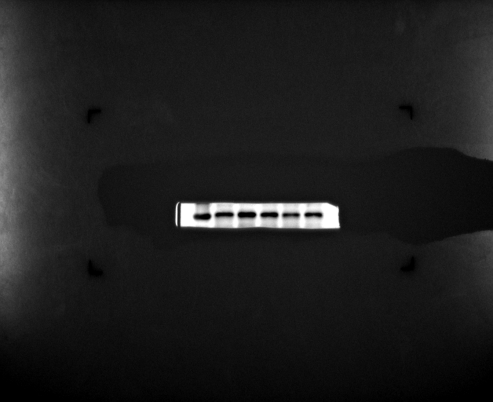


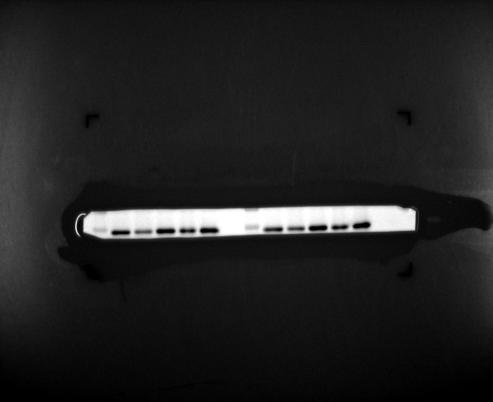
、

Caspase8


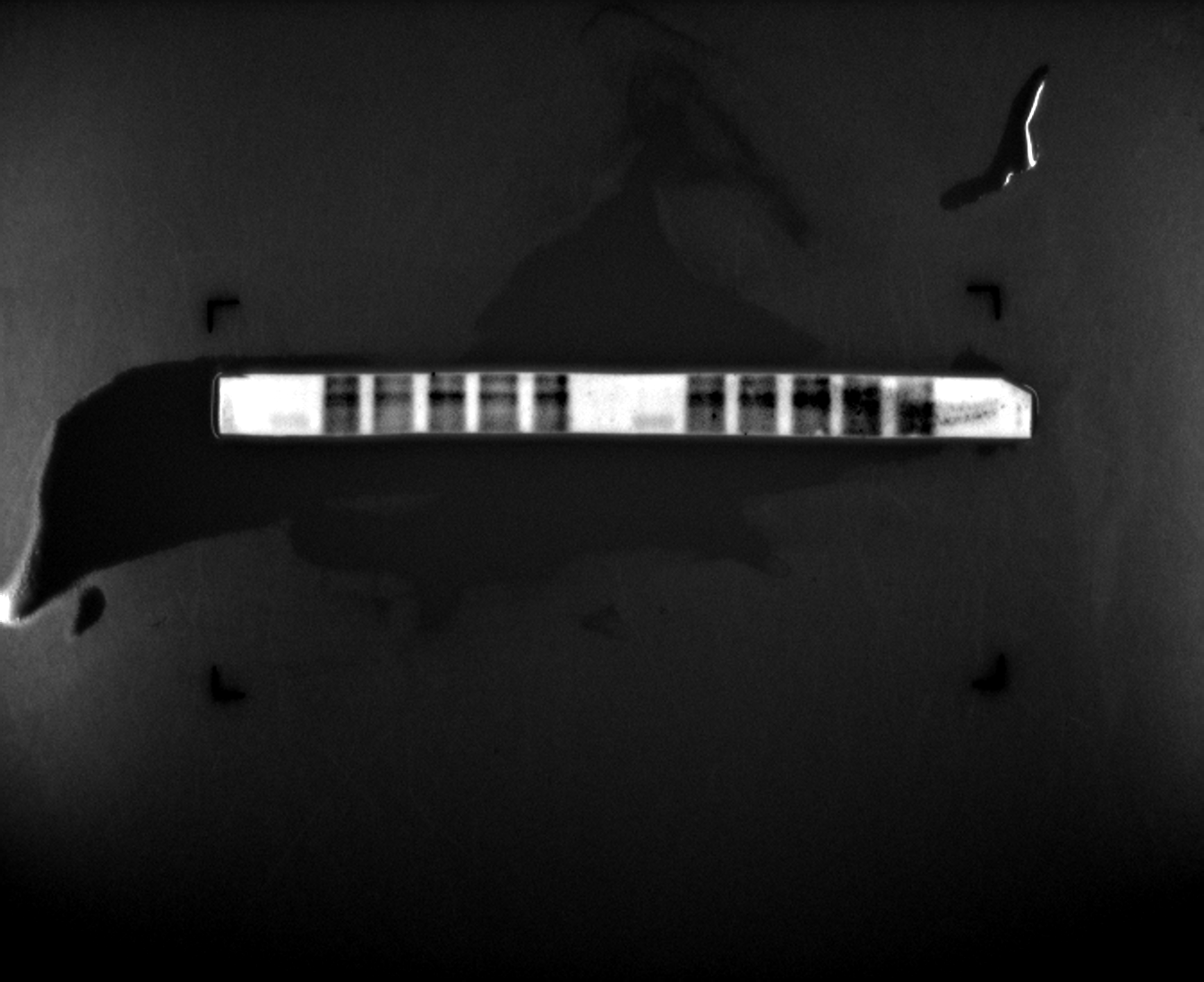

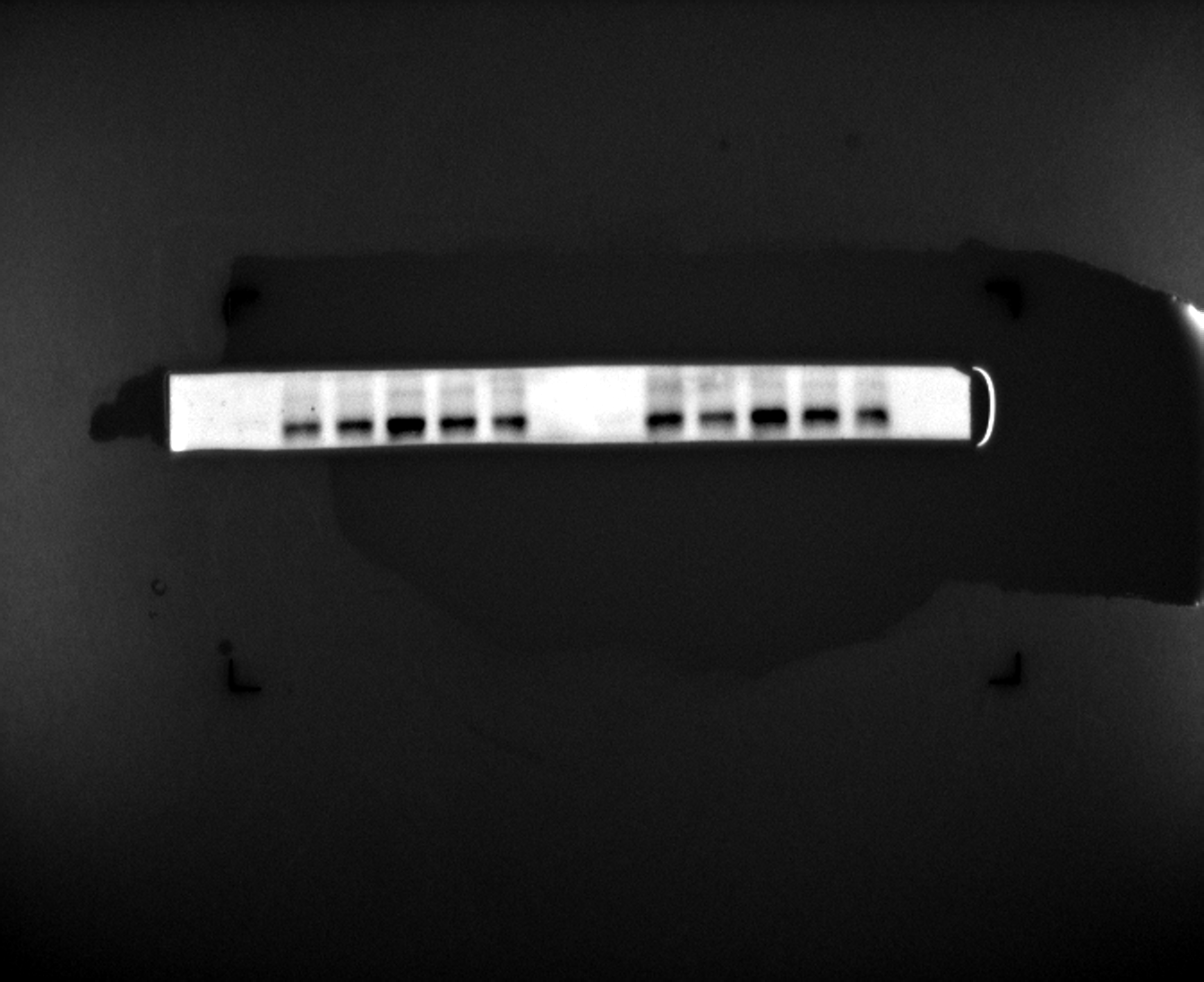


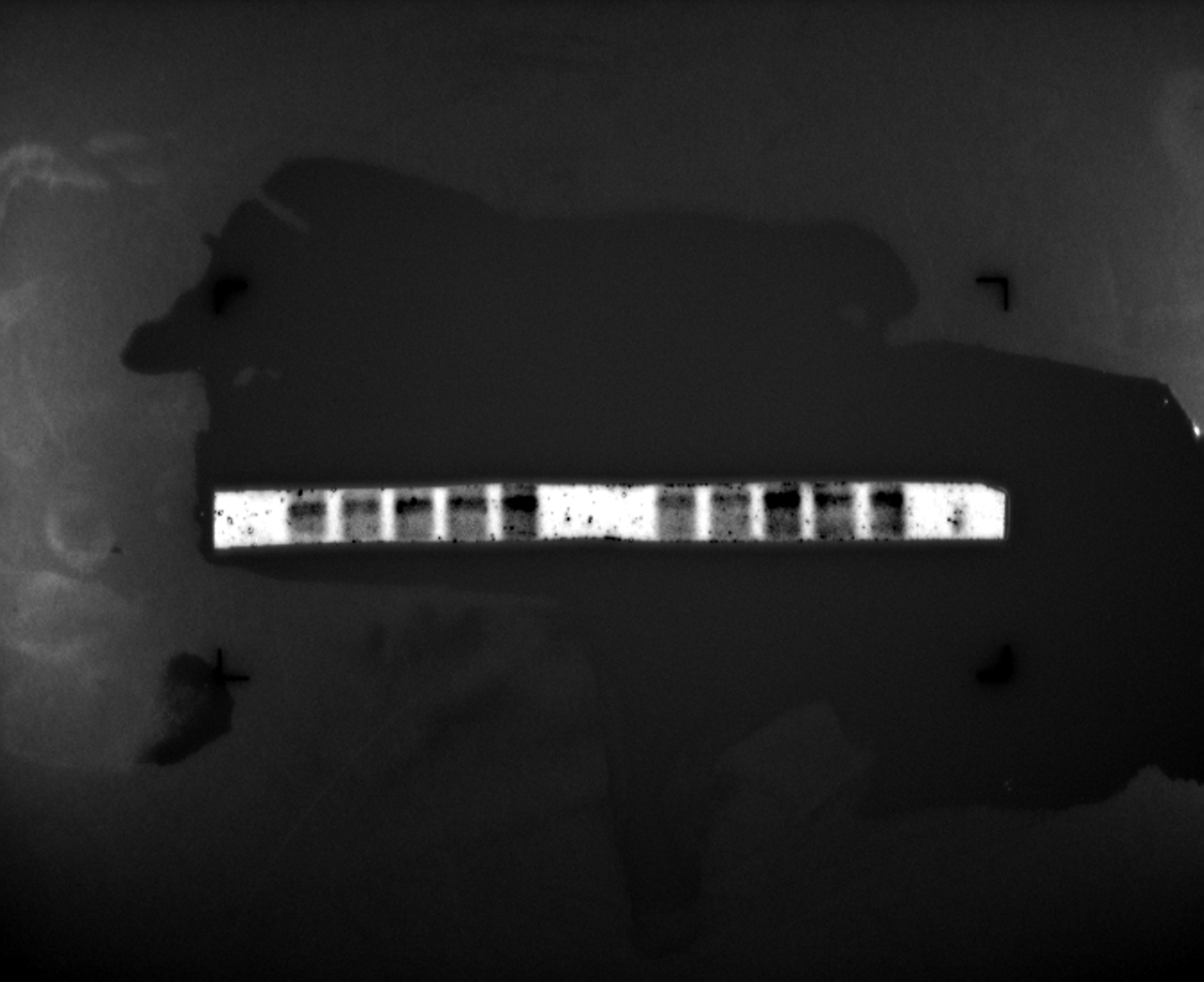


GADPH


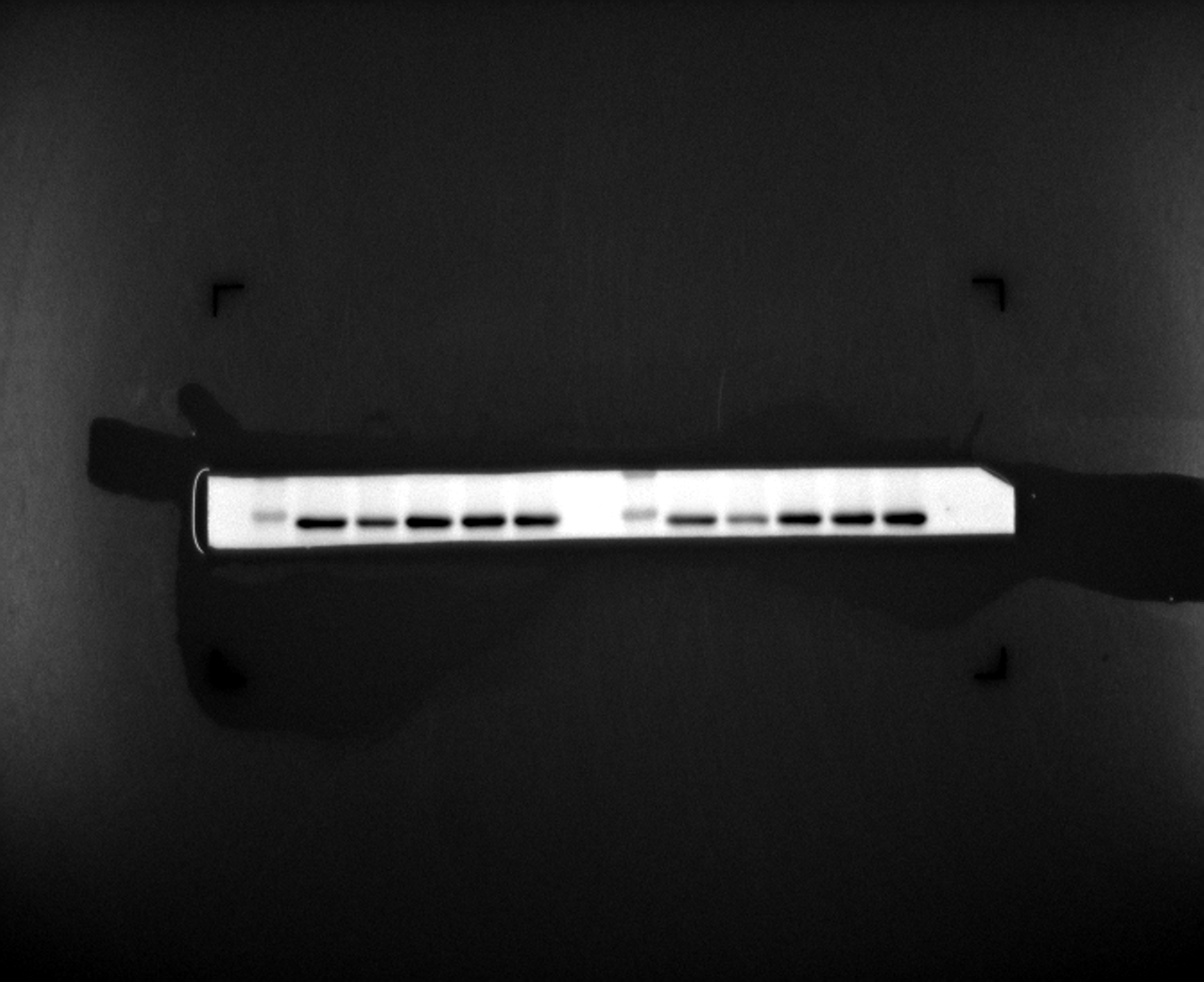

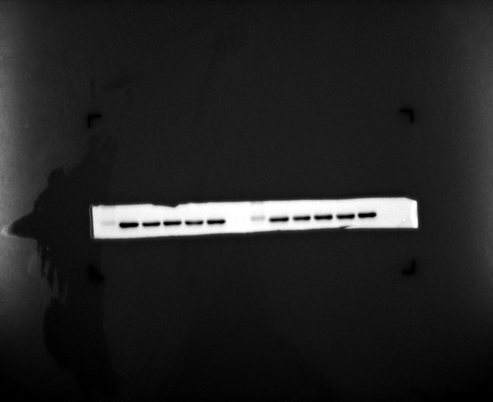


CLE-caspase8


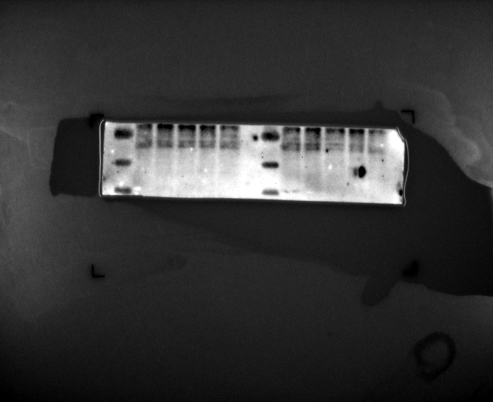

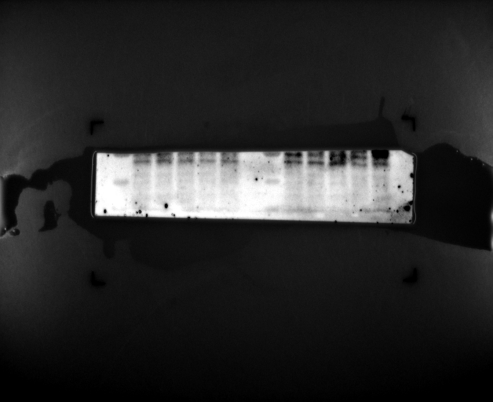


Caspase8


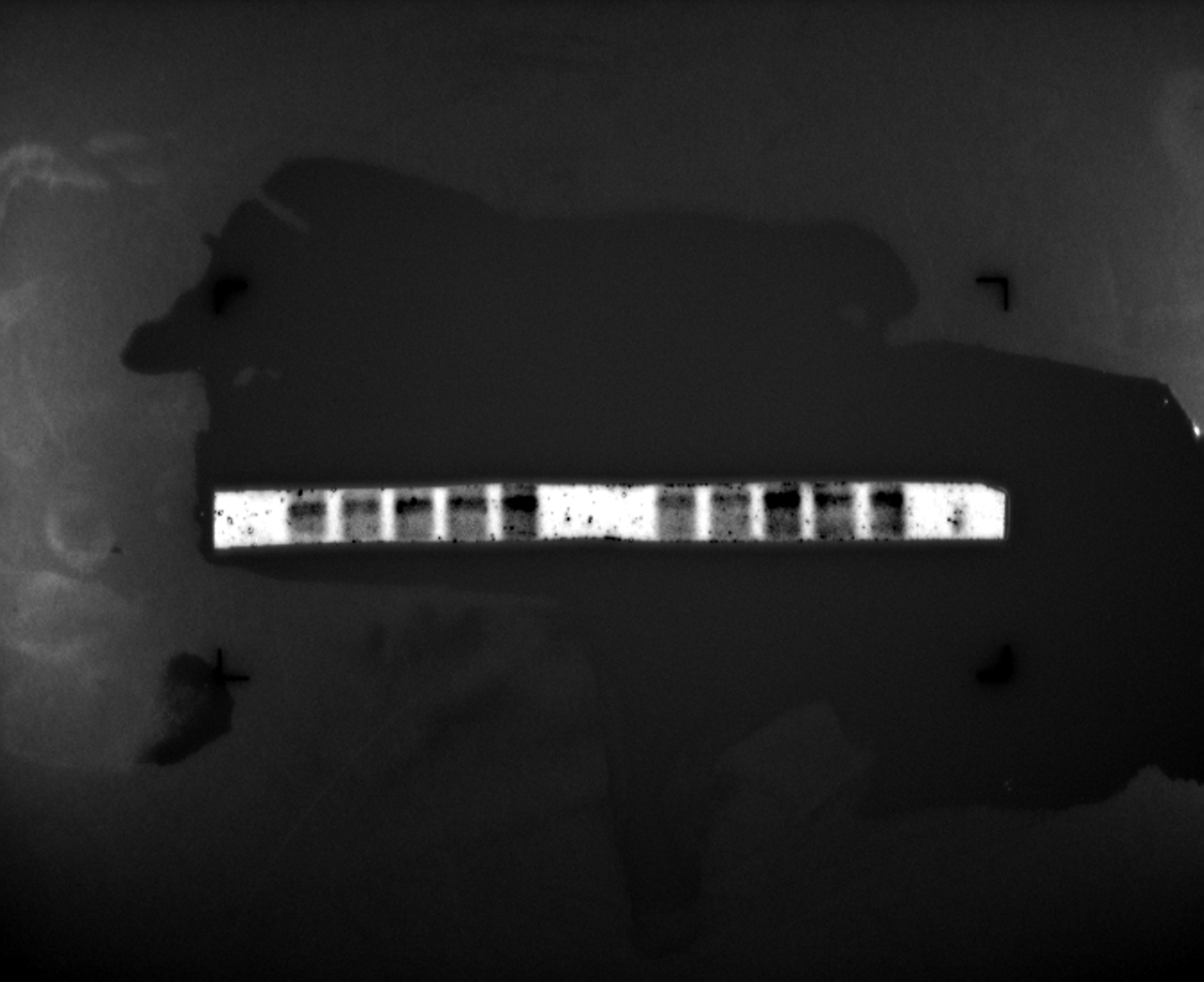

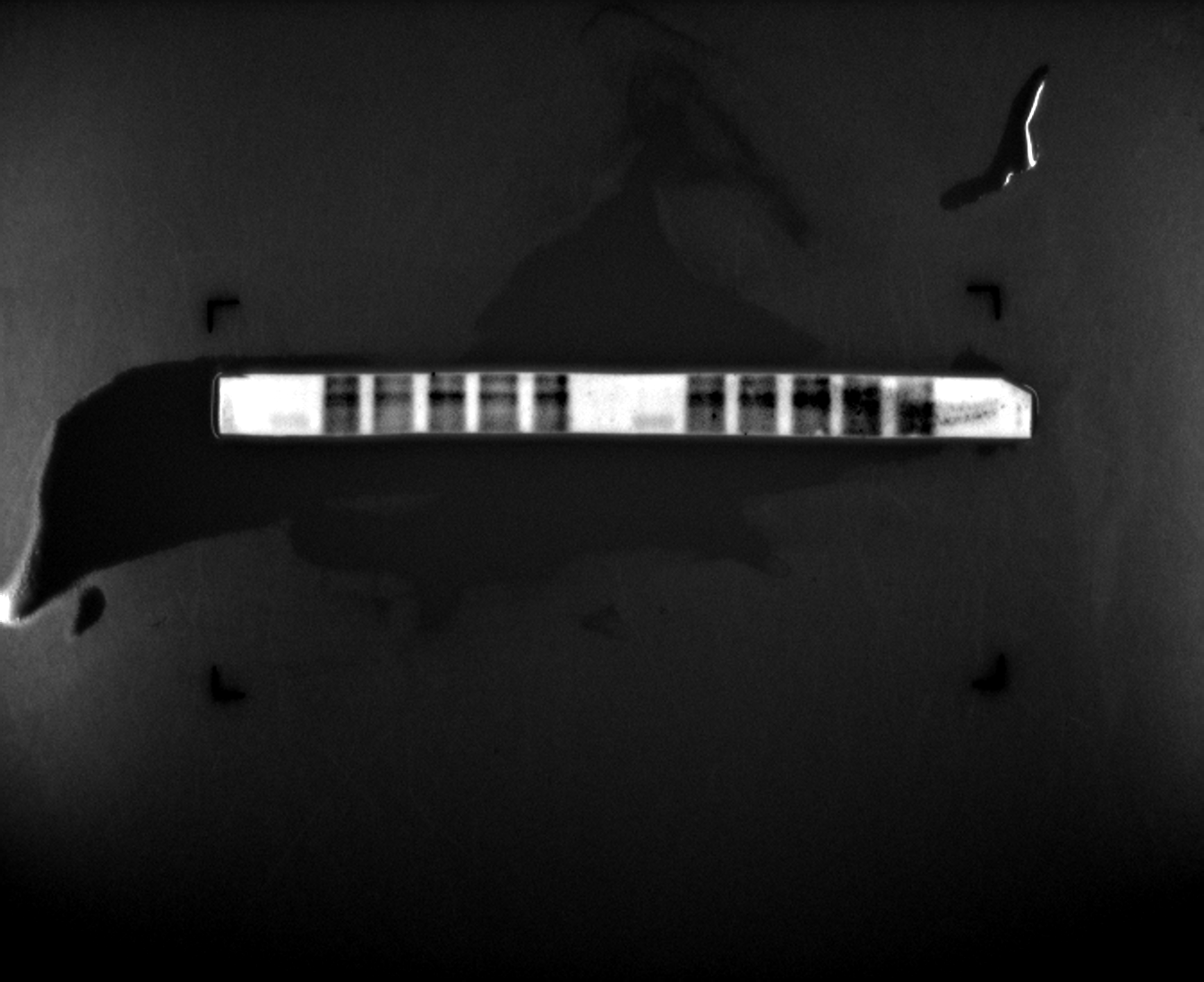


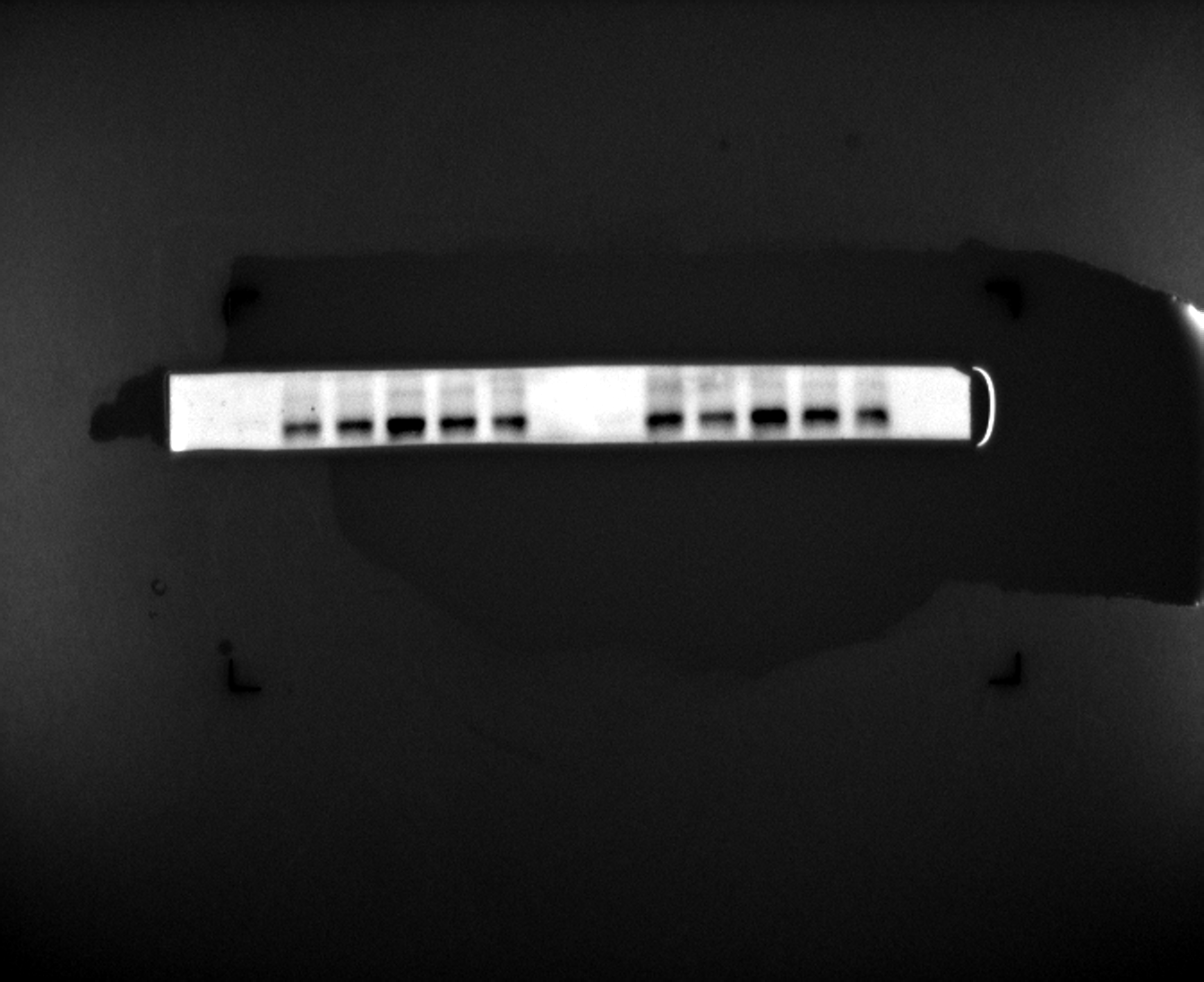


BCL2


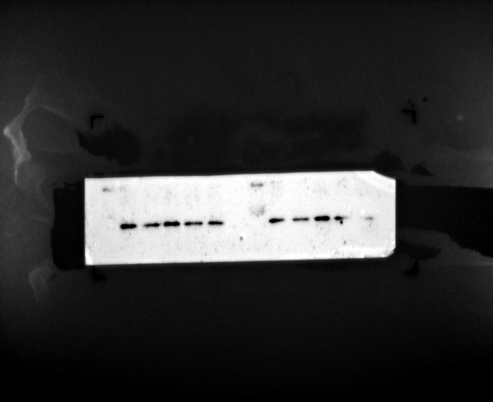

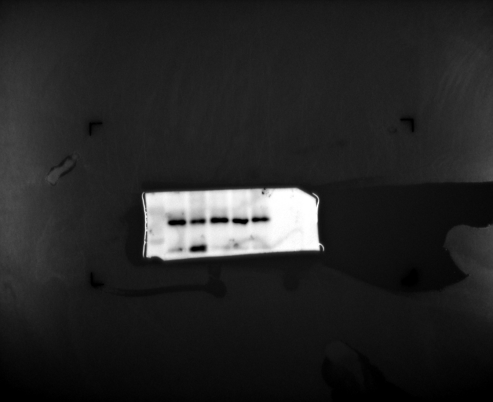


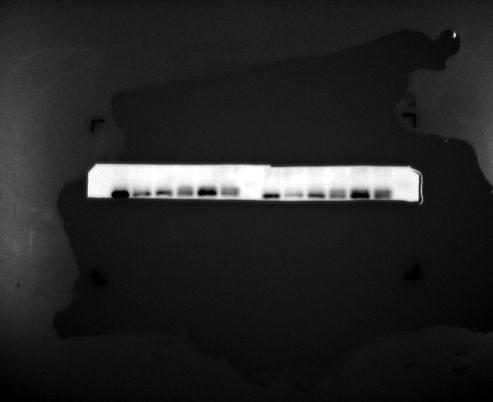


GADPH


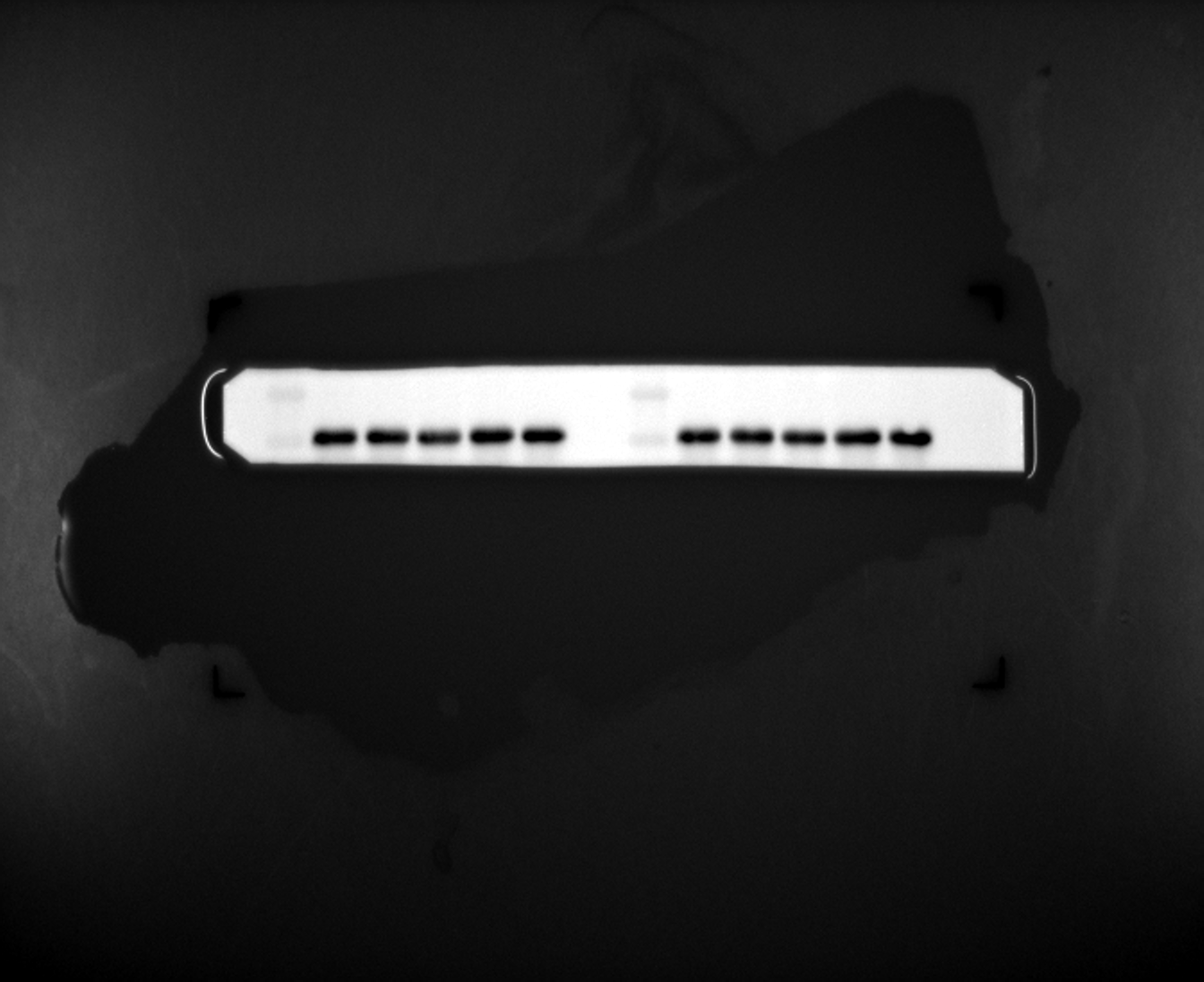

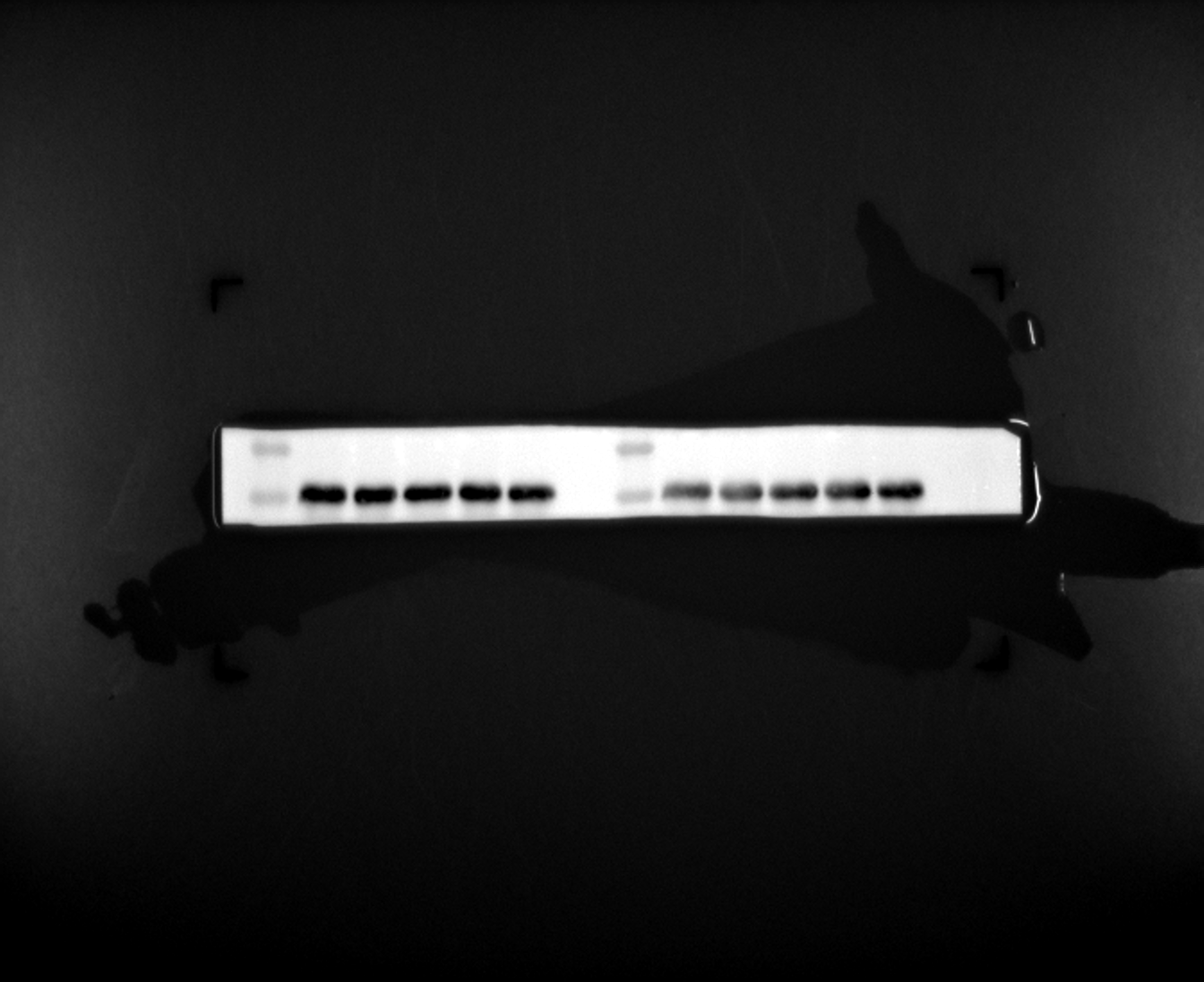


1. Bcl2


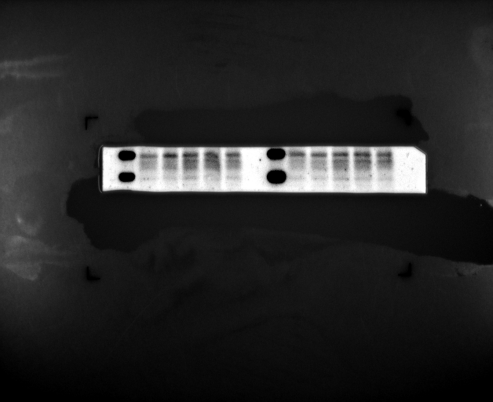

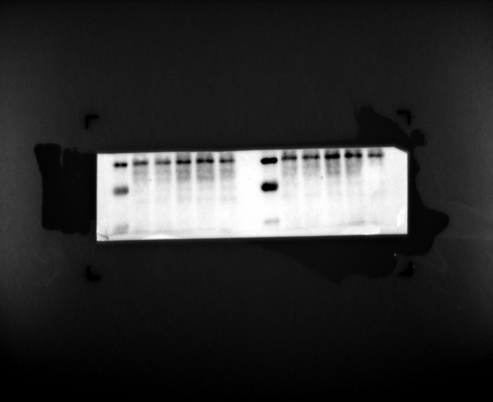


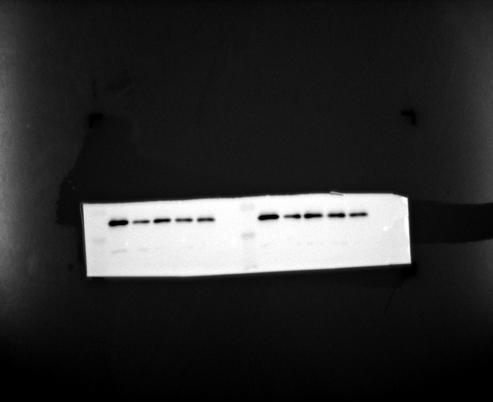


BCL2


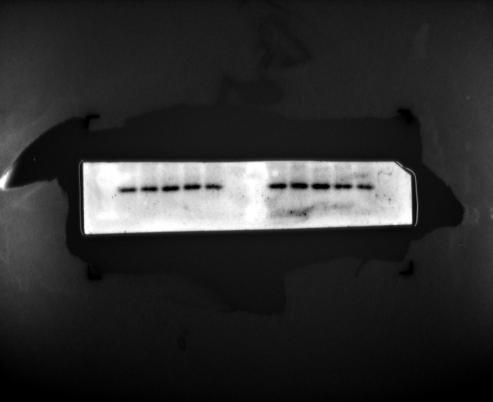

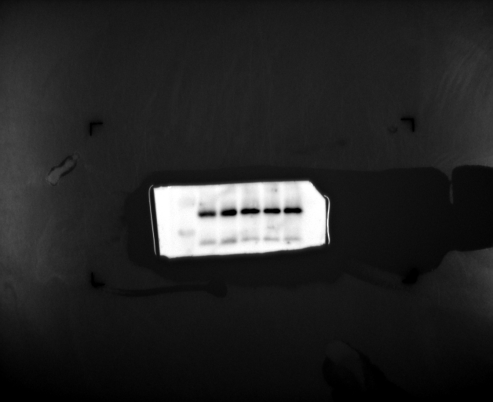


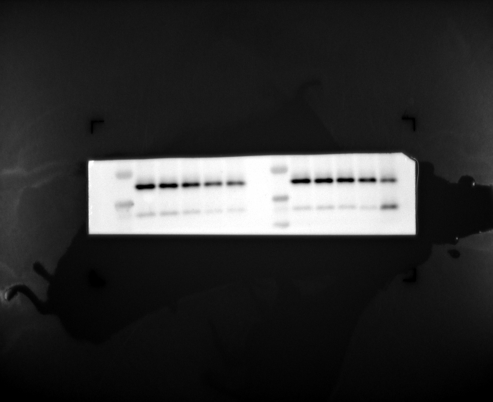


P-STAT5


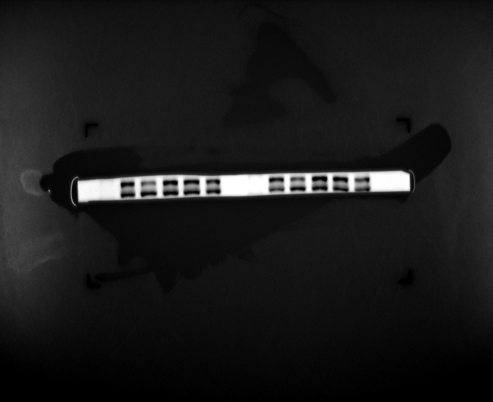

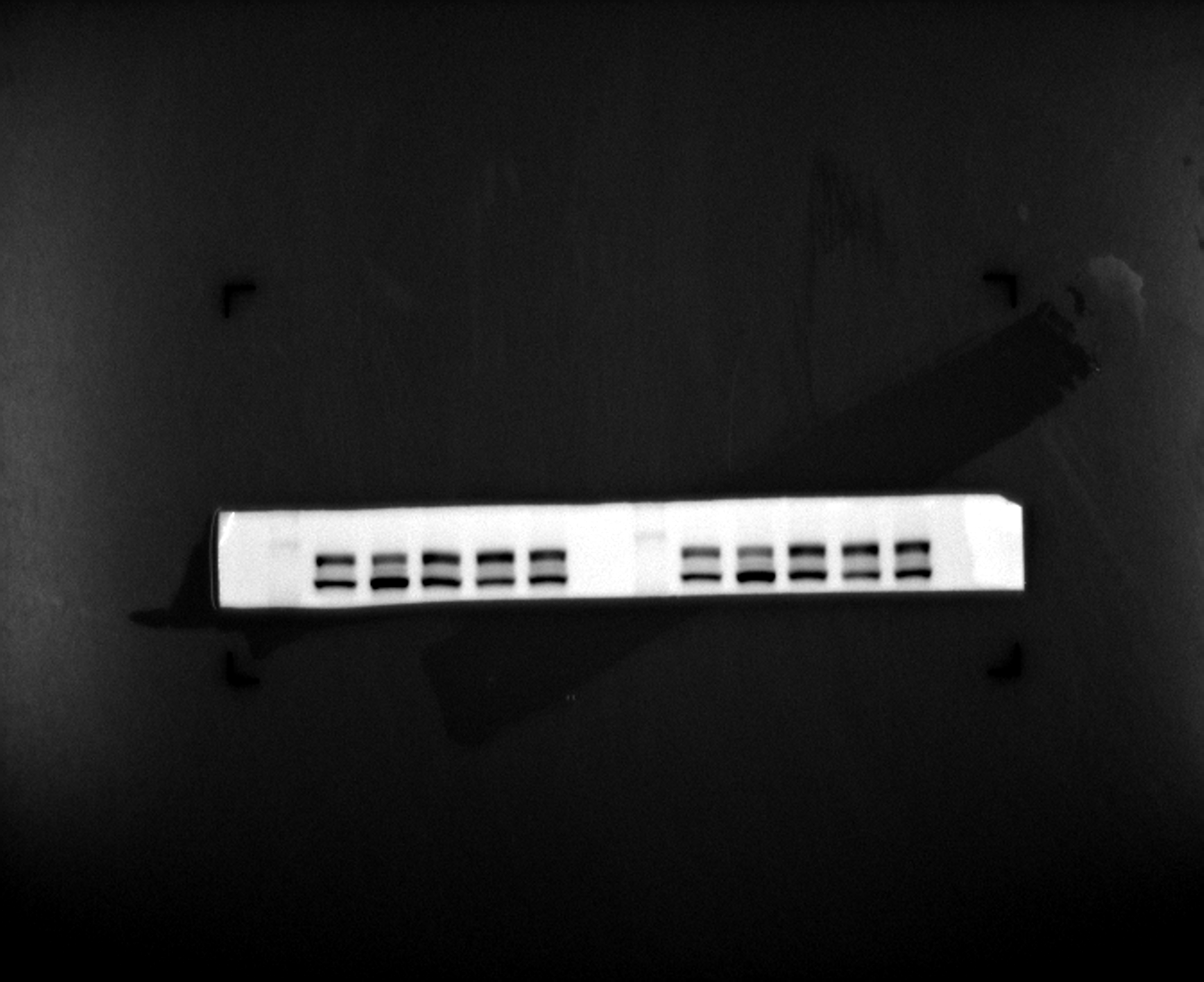


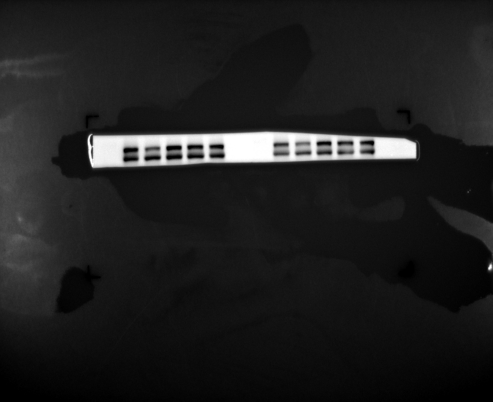


STAT5


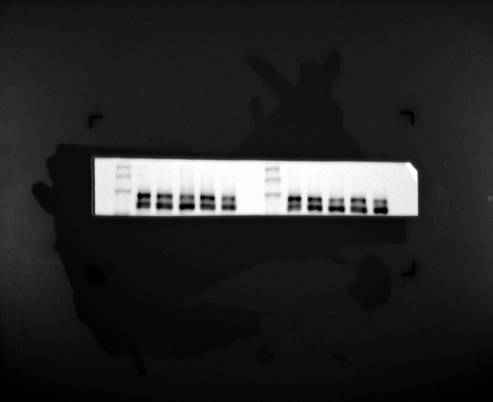

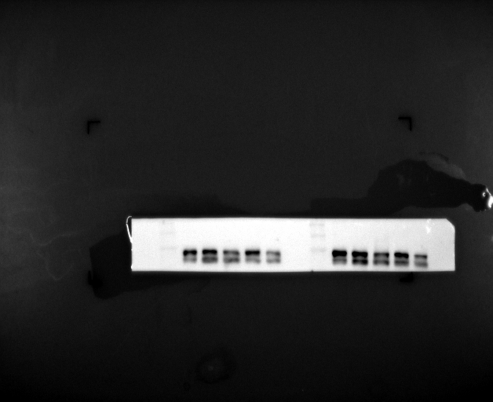


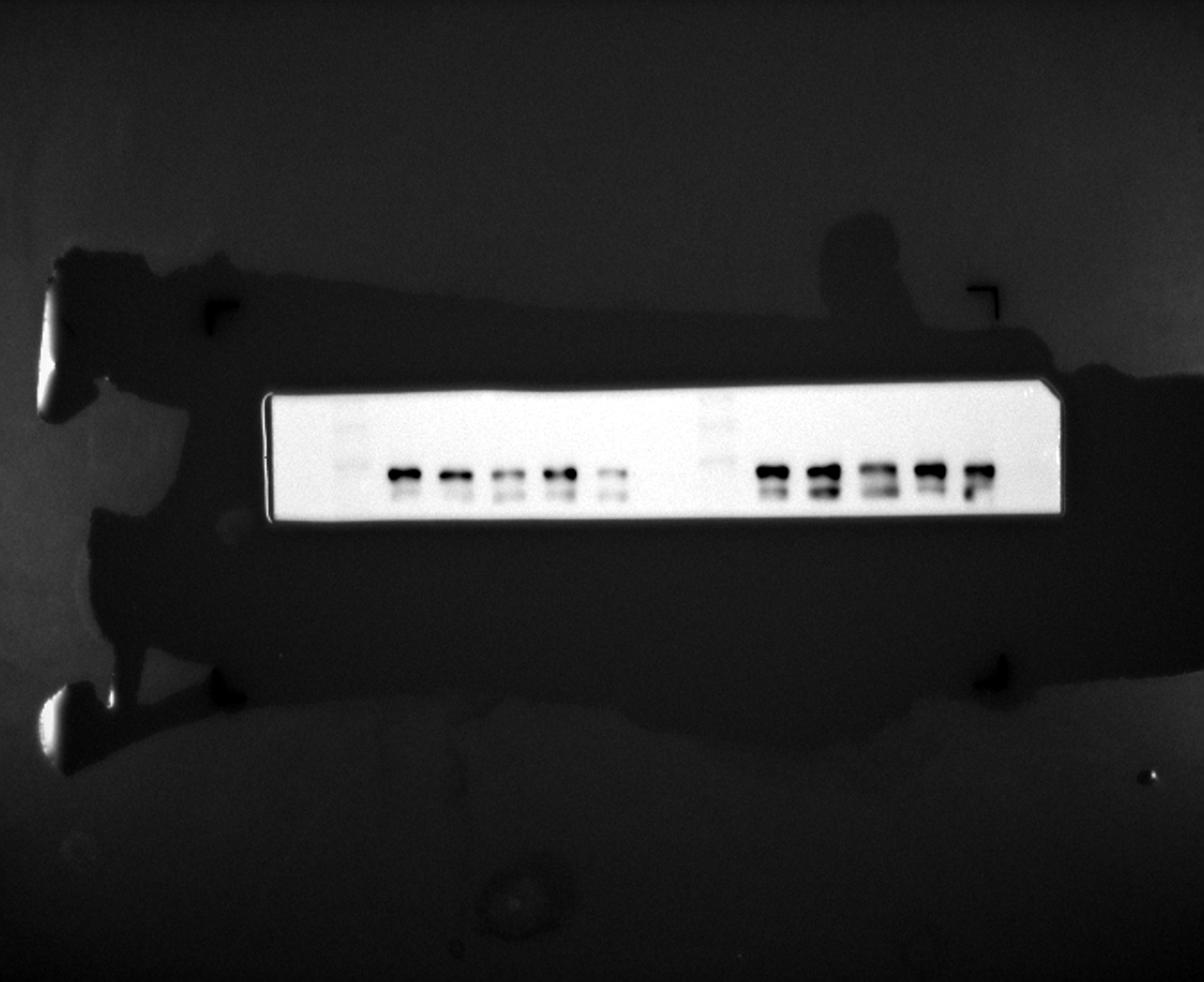


STAT5


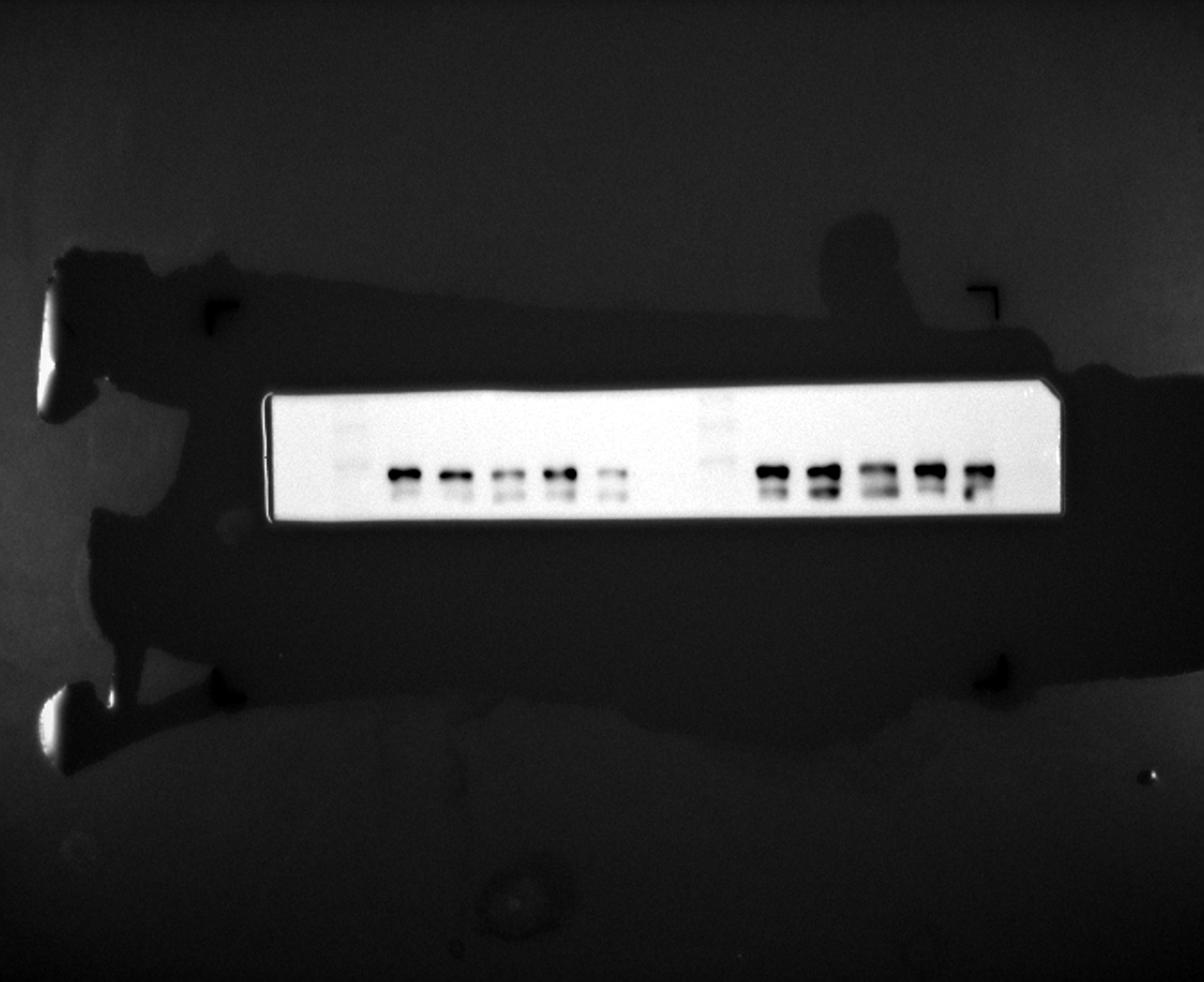

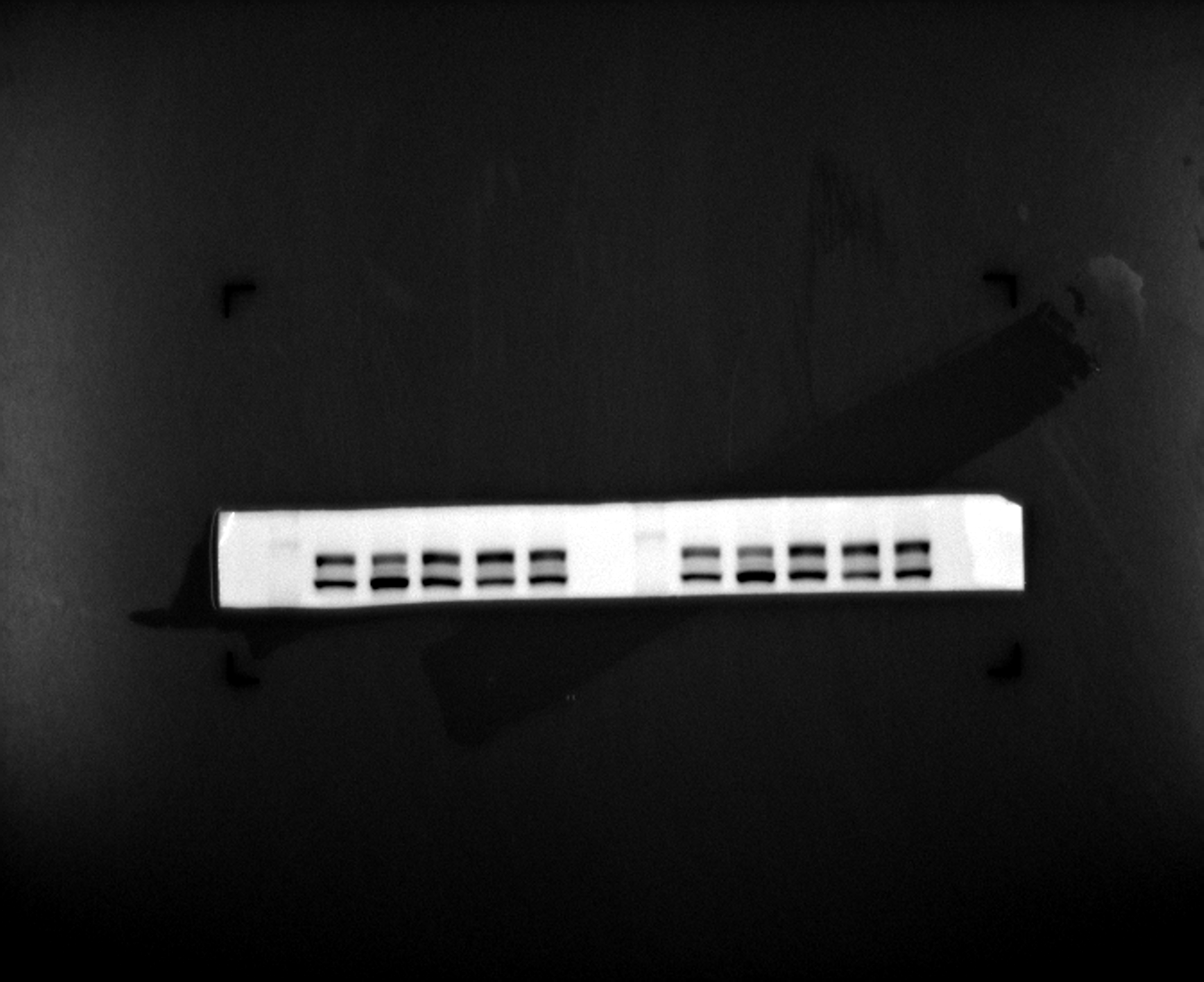


GADPH


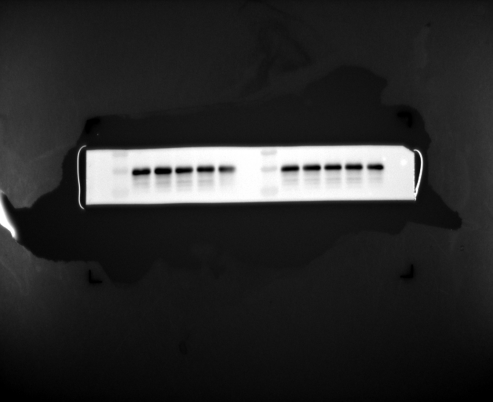

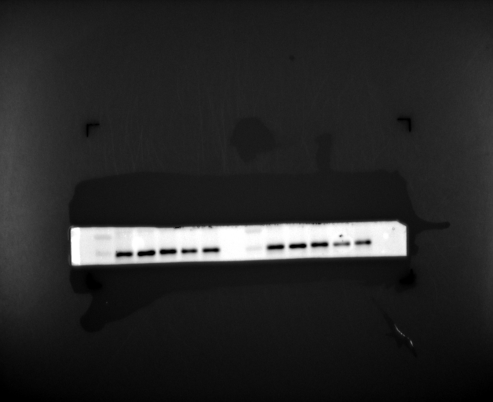


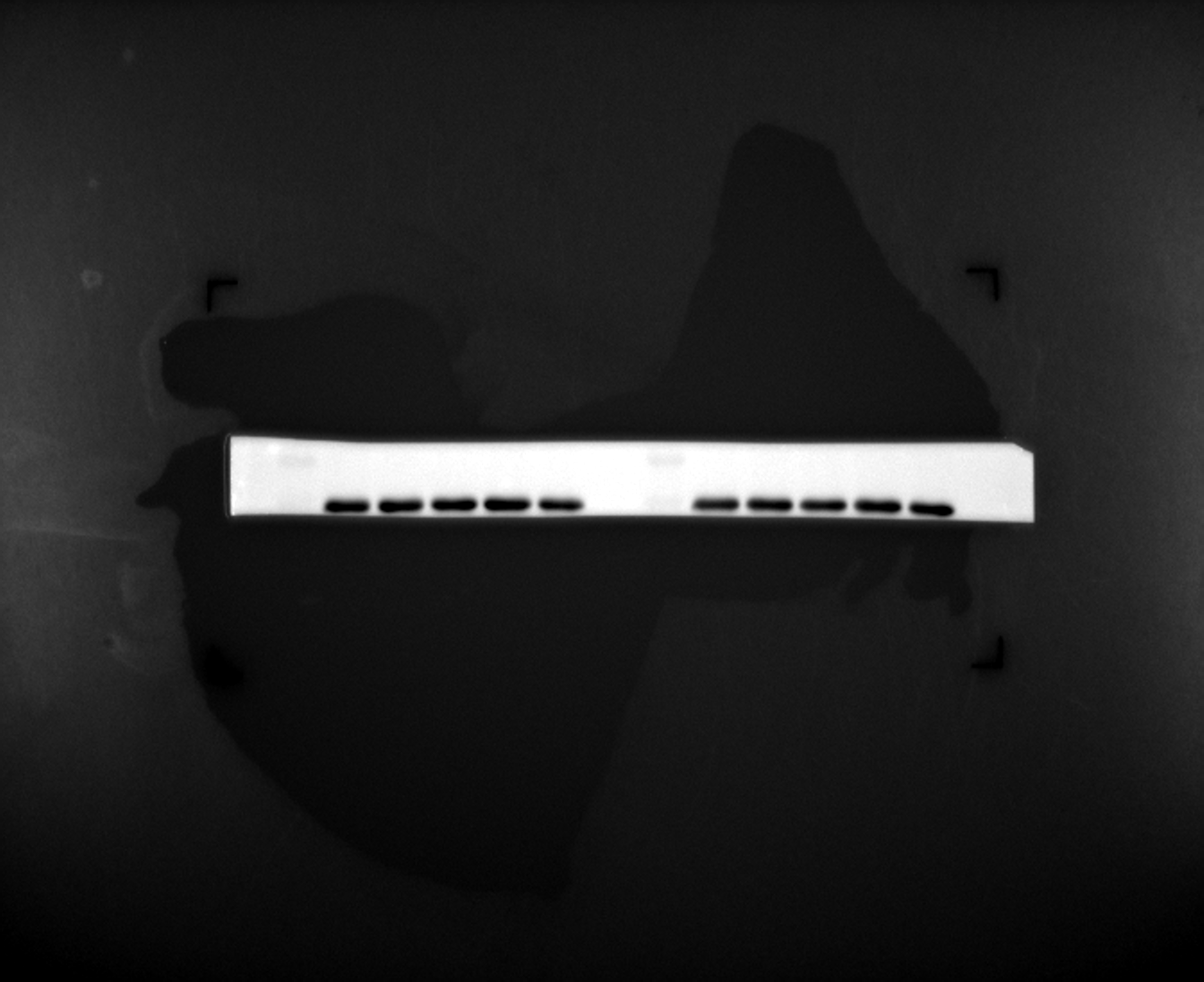

Supplement: Supplementary file 1 [file Table1.docx]
